# Integrated proteomics and metabolomics analysis of sclerosis-related proteins and femoral head necrosis following internal fixation of femoral neck fractures

**DOI:** 10.1038/s41598-024-63837-8

**Published:** 2024-06-08

**Authors:** Yang Liu, Yongsheng Ma, Wenming Yang, Qitai Lin, Yugang Xing, Huifeng Shao, Pengcui Li, Yong He, Wangping Duan, Xiaochun Wei

**Affiliations:** 1 Department of Orthopaedics, Second Hospital of Shanxi Medical University, Shanxi Key Laboratory of Bone and Soft Tissue Injury Repair, No. 382, Wuyi Road, Taiyuan, 030001 Shanxi China; 2 School of Mechanical Engineering, Hangzhou Dianzi University, Hangzhou, 310018 China; 3 Key Laboratory of 3D Printing Process and Equipment of Zhejiang Province, School of Mechanical Engineering, Zhejiang University, No. 866, Yuhang Tang Road, Hangzhou, 310027 Zhejiang China

**Keywords:** Femoral head necrosis, Proteomics, Metabolomics, Sclerosis around screw paths, Metabolomics, Proteomics

## Abstract

Femoral head necrosis (FHN) is a serious complication after femoral neck fractures (FNF), often linked to sclerosis around screw paths. Our study aimed to uncover the proteomic and metabolomic underpinnings of FHN and sclerosis using integrated proteomics and metabolomics analyses. We identified differentially expressed proteins (DEPs) and metabolites (DEMs) among three groups: patients with FNF (Group A), sclerosis (Group B), and FHN (Group C). Using the Kyoto Encyclopedia of Genes and Genomes and Gene Ontology enrichment analyses, we examined the roles of these proteins and metabolites. Our findings highlight the significant differences across the groups, with 218 DEPs and 44 DEMs identified between the sclerosis and FNF groups, 247 DEPs and 31 DEMs between the FHN and sclerosis groups, and a stark 682 DEPs and 94 DEMs between the FHN and FNF groups. Activities related to carbonate dehydratase and hydrolase were similar in the FHN and sclerosis groups, whereas extracellular region and lysosome were prevalent in the FHN and FNF groups. Our study also emphasized the involvement of the PI3K-Akt pathway in sclerosis and FHN. Moreover, the key metabolic pathways were implicated in glycerophospholipid metabolism and retrograde endocannabinoid signaling. Using western blotting, we confirmed the pivotal role of specific genes/proteins such as *ITGB5*, *TNXB*, CA II, and CA III in sclerosis and *acid phosphatase 5* and cathepsin K in FHN. This comprehensive analyses elucidates the molecular mechanisms behind sclerosis and FHN and suggests potential biomarkers and therapeutic targets, paving the way for improved treatment strategies. Further validation of the findings is necessary to strengthen the robustness and reliability of the results.

## Introduction

The accelerated pace of development in contemporary industries, construction, and transportation sectors has led to a discernible surge in the prevalence of hip fractures among the younger population, largely attributed to high-energy injuries^1^. Femoral neck fractures (FNFs) account for approximately 3.58% of all body fractures^2^. The primary treatment modality for young to middle-aged patients with FNFs is closed reduction and internal fixation using cannulated screws^3^. The refinement of imaging and internal fixation techniques has facilitated a fracture healing rate exceeding 90% post-internal fixation with cannulated screws in FNFs^4^. Nonetheless, post-internal fixation incidence of femoral head necrosis (FHN) remains substantial, ranging between 40 and 80% among middle-aged patients^5^. Furthermore, femoral head collapse has been reported in 70–80% of cases within 1–3 years following necrosis^6^.

Our previous research noted a significant degree of sclerosis surrounding the femoral screw path, as observed on computed tomography scans after prolonged retention of the internal fixation implant post-FNF surgery^7^. This densely distributed region is referred to as sclerotic cancellous bone (SCB). The severity of osteosclerosis and the propensity for osteonecrotic complications both augment the duration of implant retention, thereby disrupting regular cancellous bone development. While steroid-induced FHN samples also exhibited a sclerotic trabecular bone, the screw paths and adjacent sclerotic regions comprised approximately 50% of the femoral head volumes with aberrant sclerotic structures.

A prior finite element analysis by our team indicated that the presence of sclerosis poses a risk of femoral head collapse, even in cases of proper FNF healing^8^. The bone trabeculae surrounding the screw paths experience strains ranging from 1000 to 3000 με, which leaves these sites in a responsive osteogenic phase, leading to sclerotic bone formation. Thus, the mechanism underlying the formation of the sclerotic region can be conceptualized as follows: microdamage surrounding the implants triggers bone reconstruction, and the unstable conditions created by the implant's easy displacement facilitate the progressive thickening and expansion of sclerosis. The lower screw of the typical inverted-triangular internal fixation intensifies the stress concentration. Post-internal fixation and alterations in stress location induce adaptive remodeling of the cancellous bone, transforming the trabeculae surrounding the internal fixation into plate-like trabeculae capable of enduring increased stress. A high-density shadow was evident in this study's CT scans. Despite bone cells' responsiveness to mechanical stimuli, the transduction mechanisms accountable for metabolic changes in bone cells post-mechanical stimulation and the subsequent formation of sclerosis are not fully comprehended. Non-invasive loading of various bones in diverse animal species has been demonstrated to induce strain-related modeling responses; however, the molecular biological mechanisms driving these responses and the development of sclerosis remain largely unexplained.

Femoral head necrosis (FHN) unfolds as a complex process governed by an intricate network of key genes, proteins, and signaling pathways^9^. Often, due to nonspecific symptomatology during the initial stages, patients with FHN miss the optimal window for non-surgical intervention^10^. Hence, the timely diagnosis and detection of sclerosis assume paramount importance. An integrative analysis employing both metabolomics and proteomics to decipher the underlying mechanisms of steroid-induced FHN has not been previously undertaken. Currently, FHN is primarily diagnosed through diagnostic imaging and clinical examination. However, gradual progression of the disease frequently results in substantial joint pathology, particularly in the cancellous bone preceding diagnosis^7^. This underscores an urgent necessity to devise sensitive, specific, repeatable, and reproducible diagnostic tests for the early stages of sclerosis formation and FHN, which can augment our understanding of the underlying pathogenesis. High-throughput platforms, encompassing both metabolomics and proteomics, furnish potent methodologies for probing the underlying pathogenesis of diseases^11^. Proteomics entails a systematic, large-scale examination of proteins within biological systems to evaluate their quantities, isoforms, modifications, structures, and functions. Metabolomics deploys a systematic approach to comprehensively identify and quantify the metabolic profiles of biological samples^12^. In this study, we leveraged the state-of-the-art 4D-proteomics platform, timsTOF Pro, which fuses dual trapped ion mobility spectrometry (TIMS) and parallel accumulation–serial fragmentation (PASEF) technologies. The incorporation of an additional ion mobility separation dimension significantly augments the peak capacity and separation of isomeric peptides, thus enabling a broad dynamic range, enhanced coverage depth, precise identification of post-translational modification sites, and accurate quantification. This technique yielded a 50% increase in site quantification as compared to conventional methods^13^. The amalgamation of proteomic and metabolomic profiles has been extensively utilized to identify a multitude of proteins involved in epigenetic metabolic differences and portray the corresponding metabolic profiles, thereby effectively linking proteins to their metabolic functions^14^. Consequently, our goal was to integrate 4D proteomic and metabolomic analyses to gain a comprehensive understanding of the molecular mechanisms that underpin the correlation between proteins and their metabolic profiles across different FHN samples. This study provides persuasive evidence and lays a robust foundation for further investigation and understanding of the mechanisms underlying postoperative avascular necrosis of the femoral neck and osteonecrosis of the femoral head.

## Methods and materials

### Patients and samples

Three patients, comprising two men and one woman with a mean age of 69.33 ± 4.62 years, who underwent hip replacement for FNF during the same period, were chosen as the FNF group (Control Group, A). Three patients, comprising two men and one woman with a mean age of 62.00 ± 9.36 years, who underwent hip replacement surgery for FHN following FNF surgery at the Second Hospital of Shanxi Medical University, were included in the sclerosis group (Group B). The mean duration from internal fixation for FNF to hip replacement for FHN was 47.67 ± 14.22 months. Additionally, three patients with a mean age of 56.33 ± 2.08 years and steroid-induced FHN who underwent total hip replacement (THR) surgery at our hospital were included in the FHN group (Group C). Bone tissues of the femoral head were collected from the sclerotic area of the Group B, the same area in the Group C, and the same area in the Group A during THR. All bone tissues were stored in liquid nitrogen. This study received approval from our hospital’s ethics committee (2023 KY NO. 299), and informed consent was obtained from each participant. The FNF group comprised patients with simple Garden II, III, and IV FNFs or those aged > 60 years. Exclusion criteria encompassed patients with congenital hip dysplasia, femoral head deformities, bone tumors, and a history of internal fixation of the femoral head. Group B consisted of middle-aged patients (age < 65 years) with a history of internal fixation for FNF, and whose fractures had healed. Patients with pathological fractures, hormone usage during internal fixation, and a history of re-trauma were also excluded. Group C included patients aged 50–70 years, who had received steroid treatment prior to the onset of the disease and were diagnosed with FHN using magnetic resonance imaging and radiographs. Exclusion criteria comprised patients with osteonecrosis due to trauma prior to THR, a history of metabolic bone disease, diabetes mellitus, hypertension, coronary artery disease, or a weekly alcohol consumption exceeding 600 mL.

### Proteomic analysis

#### Protein extraction

Frozen samples (approximately 100 mg) were quickly ground into fine and uniform powder in liquid nitrogen and then homogenized in 1 mL phenol extraction buffer. Then, 1 mL saturated phenol with Tris–HCl (pH 7.8) was added. The mixture was incubated at 4 °C for 30 min with intermittent agitation, then centrifuged at 7100*g* for 10 min at 4 °C to collect the phenolic upper layer. Five volumes of pre-chilled 0.1 M (Mol) ammonium acetate–methanol solution were added, and the sample was left to precipitate at − 20 °C overnight. The precipitate was collected using centrifugation at 12,000*g* for 10 min at 4 °C, washed with methanol and acetone, and air-dried at room temperature for 5 min. The precipitate was subsequently dissolved in the lysis buffer (SDS Cracking Solution, Biyuntian, P0013G) and incubated at room temperature for 3 h. The supernatant, the total protein solution, was aliquoted and stored at − 80 °C after protein concentration measurement using bicinchonininc acid (BCA) assay.

#### Trypsin digestion and peptide desalting

Based on the determined protein concentration, a uniform concentration was adjusted using 50 μg of protein and lysis buffer. Dithiothreitol (DTT) was added to a final concentration of 5 mM, and the samples were incubated at 55 °C for 30 min. After cooling, iodoacetamide was added to a final concentration of 10 mM, and the mixture was incubated in the dark at room temperature for 15 min. Proteins were precipitated with acetone at − 20 °C for over four hours and collected by centrifugation at 8000*g* for 10 min at 4 °C. Precipitates were redissolved in Triethylammonium bicarbonate, treated with 1/50 of the sample mass of 1 mg/mL trypsin-TPCK, and digested overnight at 37 °C. The samples, after digestion, underwent lyophilization and were preserved at − 80 °C. After digestion, peptides were purified using a SOLA 96-well SPE plate, incorporating pH normalization. The column preparation involved activation with methanol followed by stabilization with distilled water. Upon loading the sample onto the column, the flow rate was meticulously controlled. A washing step with 5% methanol was performed, followed by peptide elution using methanol, resulting in 450 μL of eluate. This eluate was then subjected to vacuum evaporation. The objective of this meticulous process was to ensure the removal of mineral substances.

#### Sample processing and liquid chromatography-tandem mass spectrometry (LC–MS/MS)

The Proteomic data analysis was performed by Shanghai Luming Biological Technology Co., Ltd. (Shanghai, China). Peptide-labeled proteins were analyzed using an Agilent 1100 high-performance liquid chromatograph and TimsTOF Pro mass spectrometer (Thermo, Bruker, USA)^15^. Chromatographic conditions were set as follows: samples were loaded onto a 25-cm C18 analytical column (RP-C18, IonOpticks) at a flow rate of 300 nL/min, followed by gradient elution. The mobile phase A comprised H_2_O-FA (99.9:0.1, v/v); mobile phase B consisted of acetonitrile (ACN)-H_2_O-FA (80:19.9:0.1, v/v/v). The gradient elution conditions were set as follows: 0–66 min, 3–27% B; 66–73 min, 27–46% B; 73–84 min, 46–100% B; 84–90 min, 100% B^16^. Mass spectrometry conditions were set as follows: the capillary voltage was set at 1.4 kV, drying gas temperature at 180 °C, and drying gas flow rate at 3.0 L/min. The mass spectrum scan range was 100–1700 m/z, ion mobility range was 0.6–1.6 Vs/cm2, and collision energy range was 20–59 eV.

#### Database search

Raw LC–MS/MS files were imported into MaxQuant (version 1.6.17.0) for database search using the Andromeda search engine, and label-free quantification analysis was performed. To prevent peak mismatch, the search conditions were controlled using the false discovery rate (FDR). The UniProt-reviewed _yes+taxonomy_9606.fasta database (January 2021) was used in the present study. The following search parameters were used: trypsin was used as the digestion enzyme with up to 2 missed cleavages, carbamidomethyl (C) as the fixed modification, and oxidation (M) as the variable modification. The precursor ion mass tolerance was set to 20 ppm, and the fragment ion mass tolerance was set to 0.1 Da. The results were filtered based on an FDR ≤ 1% for peptides and proteins.

#### Bioinformatics analysis

Raw data were obtained through a database search, retaining proteins with an expression proportion of ≥ 50% in all sample groups. Proteins with ≤ 50% missing values were expressed as the mean of the same group of samples. Reliable proteins were obtained after median normalization and log2 transformation. Differentially expressed proteins (DEPs) were identified according to the following screening criteria: fold change (FC) = 1.2 times and p-value < 0.05. FC = 0 and FC = inf were considered as “presence/absence” differences between groups. Upon identifying DEPs, principal component analysis (PCA) was performed to verify homogeneity within groups and heterogeneity between them. DEPs were subject to Gene Ontology/Kyoto Encyclopedia of Genes and Genomes database (GO/KEGG) enrichment analyses to determine their functions. Pathway analyses were performed using the KEGG database (http://www.kegg.jp/KEGG/pathway.html). A protein–protein interaction (PPI) network was generated using the STRING database (http://string.embl.de), and the Cytoscape web application (Version 1.0.4, http://www.cytoscape.org) was used to visualize the functional analysis information at four levels: protein folding changes, PPI, KEGG pathway enrichment, and biological process enrichment.

### Metabolomics

#### Metabolite extraction

The metabolomic data analysis was performed by Shanghai Luming Biological Technology Co., Ltd. (Shanghai, China). The tissue sample weighing 30 mg was introduced into a 1.5-mL EP tube, which was then supplemented with 20 μL of an internal standard (l-2-chlorophenylalanine at 0.06 mg/mL in a methanol solution) and 400 μL of a methanol–water mixture (v/v, 4:1)^17^. Following this, two small steel balls were incorporated into the tube. Subsequently, after a 2-min pre-cooling period in a refrigerator set at – 20 °C, the tube was subjected to grinding at 60 Hz for 2 min. The extract was sonicated in an ice-water bath for 10 min and left to stand at − 20 °C for 30 min. The sample was then centrifuged for 10 min (13,000 rpm, 4 °C), and 300 μL of the supernatant was loaded into an LC–MS feed vial and evaporated. It was then resolubilized with 300 μL methanol–water (1:4, v/v) (vortexed for 30 s and sonicated for 3 min) and allowed to stand for 2 h at 20 °C. Then, the sample was centrifuged for 10 min (13,000 rpm, 4 °C), and 150 μL of the supernatant was aspirated using a syringe, filtered using a 0.22 μm organic phase pinhole filter, transferred to an LC injection vial, and stored at − 80 °C until LC–MS analysis was conducted.

#### LC–MS/MS analysis

Nexera ultra-high-performance liquid chromatograph (SHIMADZU, Kyoto, Japan)/Q-Exactive high-resolution tandem mass spectrometer equipped with heated electrospray ionization (ESI) source (Thermo Fisher Scientific, Waltham, MA, USA) was used to analyze the metabolic profiling in both ESI-positive and ESI-negative ion modes. An ACQUITY UPLC HSS T3 column (1.8 μm, 2.1 × 100 mm) was employed in positive and negative modes. The binary gradient elution system consisted of (A) water (containing 0.1% formic acid, v/v) and (B) ACN (containing 0.1% formic acid, v/v) and separation was achieved using the following gradient: 0.01 min, 5% B; 2 min, 5% B; 4 min, 30% B; 8 min, 50% B; 10 min, 80% B; 14 min, 100% B; 15 min, 100% B; 15.1 min, 5% and 16 min, 5% B. The flow rate was 0.35 mL/min, and the column temperature was 45 °C. All the samples were kept at 10 °C during the analysis. The injection volume was 2 μL.

The mass range was from m/z 100 to 1200. The resolution was set at 70,000 for the full MS scans and 17,500 for HCD MS/MS scans. The collision energy was set at 10, 20, and 40 eV. The mass spectrometer operated as follows: spray voltage, 3500 V (+) and 3000 V (−); sheath gas flow rate, 35 arbitrary units; auxiliary gas flow rate, 8 arbitrary units; capillary temperature, 320 °C; Aux gas heater temperature, 350 °C; S-lens RF level, 50.

#### Data processing and bioinformatics analysis

The original LC–MS data were processed by Progenesis QI V2.3 (Nonlinear, Dynamics, Newcastle, UK) for baseline filtering, peak identification, integral, retention time correction, peak alignment, and normalization. The main parameters of 5 ppm precursor tolerance, 10 ppm product tolerance, and 5% product ion threshold were applied. Compound identification was based on the precise mass-to-charge ratio (M/z), secondary fragments, and isotopic distribution using the Human Metabolome Database, Lipidmaps (V2.3), Metlin, and self-built databases. The extracted data were then further processed by removing any peaks with a missing value (ion intensity = 0) in more than 50% of groups, by replacing the zero value with half of the minimum value, and by screening according to the qualitative results of the compound. Compounds with scores below 36 (out of 60) points were deemed inaccurate and removed. A data matrix was combined from the positive and negative ion data^17^.

### Western blot

For bone tissue, specialized steel grinding jars and adapters were utilized. The bone tissue was frozen in liquid nitrogen for 5–10 min, then placed in the grinding jar with steel balls, and ground at a frequency of 70 Hz for 180 s (with cycle grinding set). After grinding, the tissue was transferred to a 2 mL grinding tube, extraction liquid was added, and 2 pieces of 3-mm grinding beads were used to homogenize for 60 s. Tissues were lysed in a RIPA buffer system (Servicebio, Wuhan, China) containing a protein hydrolase inhibitor 50× cocktail (Servicebio) on ice. The supernatant was collected, and the total protein was determined using the BCA protein assay. Equal amounts of protein (5 μg/μl) were loaded onto sodium dodecyl sulfate–polyacrylamide gels. The proteins isolated in the gel were transferred to a PVDF (0.45 um) membrane. The membranes were incubated in 5% skim milk for 30 min. Following blocking, the membranes were incubated overnight at 4 °C with primary antibodies against integrin beta-5 (ITGB5) (1:3000; A04201-1, Boster, Wuhan, China), tenascin-X (TNXB) (1:3000; GB11338, Servicebio, Wuhan, China), acid phosphatase 5 (ACP5) (1:2000, GB12416, Servicebio, Wuhan, China), cathepsin K (CTSK) (1:3000, PB9856, Boster, Wuhan, China), carbonic anhydrase (CA2) (1:3000, GB112426, Servicebio), carbonic anhydrase 3 (CA3) (1:1000, GB113775, Servicebio), and glyceraldehyde 3-phosphate dehydrogenase (GAPDH) (1:2000, GB15002, Servicebio). The membranes were washed with wash buffer (TBST, 0.05% Tween-20). After incubation with horseradish peroxidase-coupled secondary antibodies for 30 min at room temperature, signals were generated using enhanced chemiluminescence reagents (Wuhan, Servicebio) and detected using a ChemiDoc CRS imaging system (Bio-Rad, Hercules, CA, USA)^18^. Western blot data were analyzed using ImageJ software.

### Integrated analysis of proteomics and metabolomics

For this analysis, the top 20 differentially expressed proteins and metabolites (sorted by p-value, or all if fewer than 20) were selected. The Pearson correlation algorithm was applied to their relative abundance data to calculate the interrelations between proteins and metabolites. Correlation heatmaps were generated to visualize the results of the associative analysis between differential proteins and metabolites. KEGG Markup Language (KGML) files, a subset of the KEGG database, encompass both the relationships among graphical objects within KEGG pathways and information on orthologous genes from the KEGG GENES database. This facilitated the elucidation of the network relationships between proteins and metabolites, enabling a more systematic exploration of the interactions between the proteome and metabolome. Networks were drawn based on Pearson correlation analysis for protein and metabolite response intensity data, selecting relationships with pvalue ≤ 0.05 and correlation ≥ 0.95.

### Statistical analysis

Statistical analysis was performed using GraphPad Prism 7.0 (GRAPHPAD SOFTWARE, San Diego, CA, USA), and data were expressed as mean ± standard deviation. Differences between two or more groups were assessed using the Student's t-test or one-way ANOVA, followed by Tukey's post-hoc test. Pearson's correlation analysis confirmed the significance of the correlation between two variables. Statistical significance was set at P < 0.05.

### Ethics approval and consent to participate

The experimental protocol was established, according to the ethical guidelines of the Helsinki Declaration and was approved by the Human Ethics Committee of the Second Hospital of Shanxi Medical University.

## Result

### Identified DEPs

**Figure 1:**
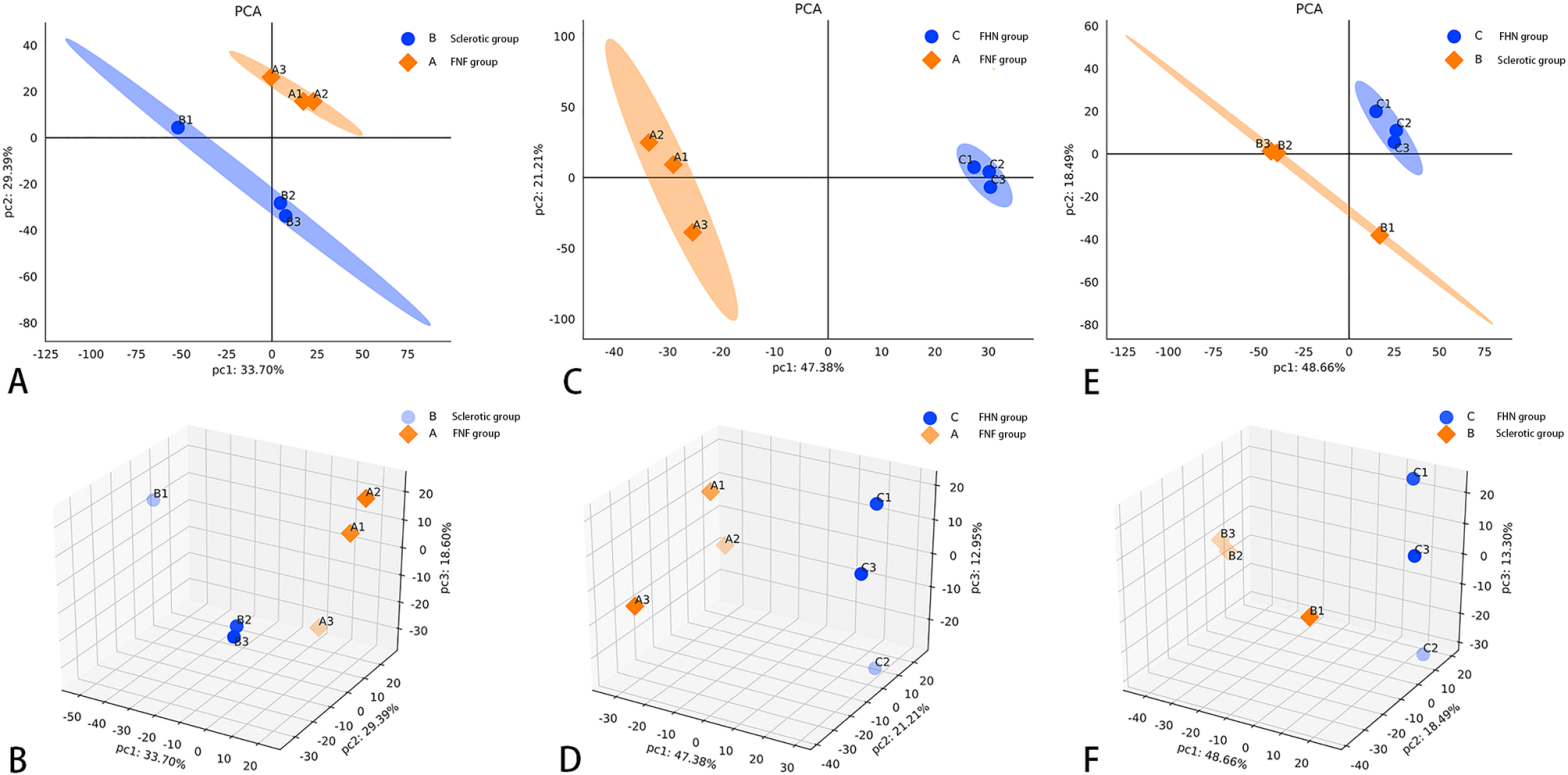
Principal component analysis (PCA) of credible protein expression levels. Visualizing the relationship between samples from different dimensions. AB: Sclerosis group (B group) vs. FNF group (C group); CD: FHN group (C group) vs. FNF group (B group); EF: C group vs. B group. Each point in the figure represents a replicate from a group experiment, with different colors representing different groups. If the difference between two samples is significant, the coordinate points on the score plot will be relatively far apart.

**Figure 2:**
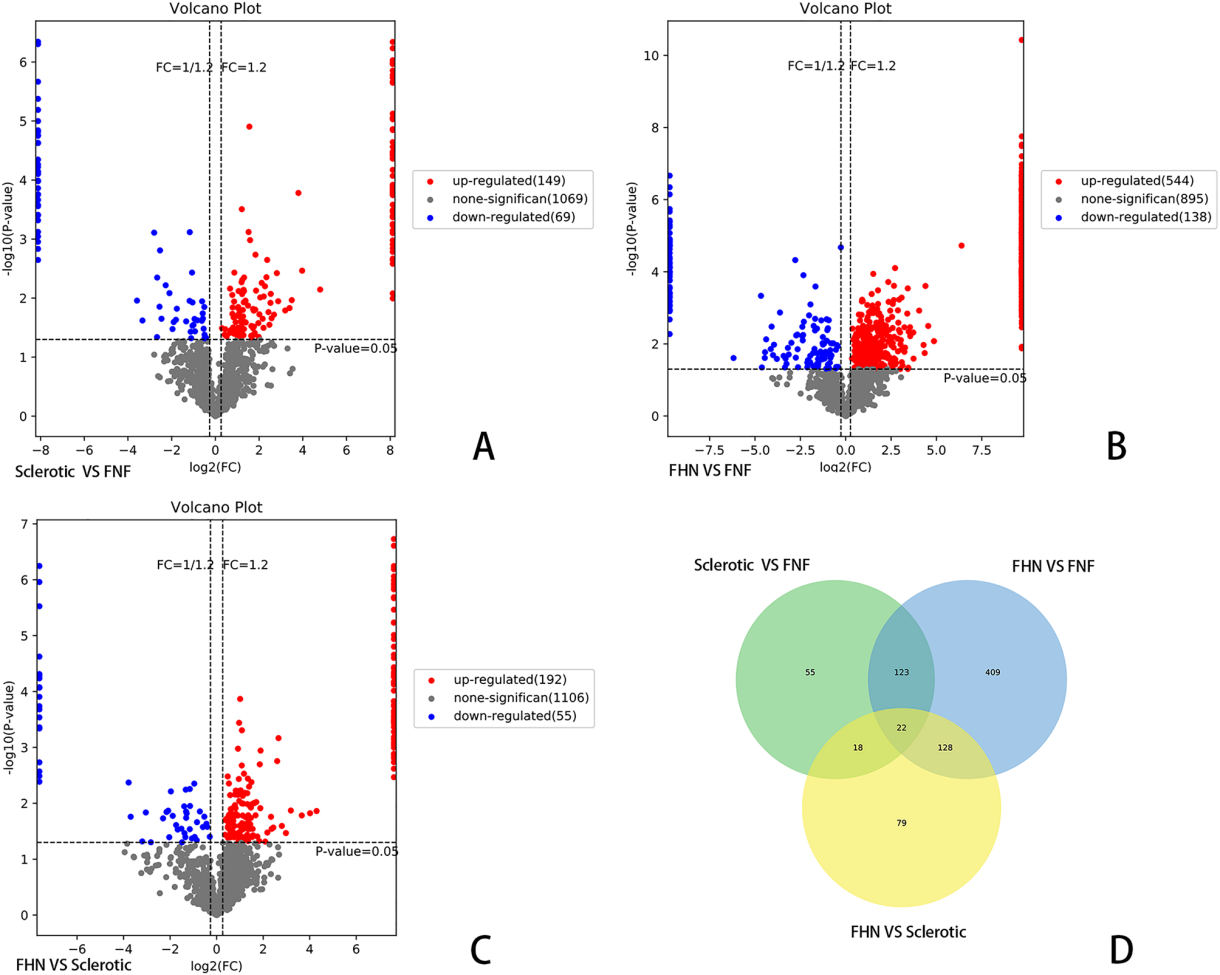
Volcano plots and Venn diagrams of differentially expressed proteins. (**A**–**C**) Volcano plots of differentially expressed proteins between the FNF (Group A), sclerosis (Group B), and FHN groups (Group C). The x-axis represents log2(FC), with larger values indicating greater differences. The right side represents upregulation, and the left side represents downregulation. The y-axis represents –log10(p-value), with larger values indicating greater differences. Blue points represent downregulated differentially expressed proteins, red points represent upregulated differentially expressed proteins, and gray points represent non-significant differentially expressed proteins. (**D**) Venn diagram of the three groups. Venn analysis was performed to assess the characteristics and commonalities of differentially expressed proteins among the groups, facilitating subsequent experimental design and selection of research directions. Different colors represent different groups, and the numbers in the figure represent the number of protein intersections and the number of proteins unique to each group.

Raw data were obtained using database search, and proteins with ≥ 50% of expression values in any group of samples were retained. Proteins with missing values ≤ 50% were filled with the mean value of the same group of samples, and the plausible proteins were obtained by median normalization and log2 logarithmic transformation. The expression levels of reliable proteins were used to perform PCA, which demonstrated relationships between samples from different dimensions (Fig. 1), and the covariance data of PCA are shown in Table S1. In this study, 2562 proteins and 21,305 unique peptides were identified in the three types of cancellous bone. An FC of |log2|> 1.2 and a p-value < 0.05 were set as the screening thresholds to distinguish differences between the hardening and control groups. We identified 149 upregulated and 69 downregulated proteins between the hardening and FNF groups (Table S2), 544 upregulated and 138 downregulated proteins between the necrosis and fracture groups (Table S3), and 192 upregulated and 55 downregulated proteins between the necrosis and sclerosis groups (Table S4). The top 20 upregulated and downregulated DEPs in the three groups are listed in Tables 1, 2, 3, 4, 5, 6. We generated volcano plots to display the DEPs in each group (Fig. 2A–C). Based on the expression profiles, the co-expression data were grouped, and unsupervised hierarchical clustering was performed using the R software. A heat map of the clustered analysis of different comparison groups is shown in Fig. S1. The Pearson algorithm was used to calculate the correlation between the DEPs. A correlation coefficient close to 1 indicates a high similarity in expression patterns between the proteins. Correlation analysis of the top 50 significantly different proteins (ranked by p-values) is shown in Fig. S2. A Venn diagram showed that 22 DEPs were detected at the protein level across all three groups (Fig. 2D).

**Table 1:** TOP20 upregulation in differentially expressed proteins in sclerotic group versus samples from FNF group.

Accession	Protein name	Gene name	P value
A0A0C4DH38	Immunoglobulin heavy variable 5–51	IGHV5-51	0.00007
O00391	Sulfhydryl oxidase 1	QSOX1	0.00003
O14498	Immunoglobulin superfamily containing leucine-rich repeat protein	ISLR	0.00226
O15460	Prolyl 4-hydroxylase subunit alpha-2	P4HA2	0.00026
O94925	Glutaminase kidney isoform, mitochondrial	GLS	0.00000
P01817	Immunoglobulin heavy variable 2–5	IGHV2-5	0.00012
P05154	Plasma serine protease inhibitor	SERPINA5	0.00123
P12107	Collagen alpha-1(XI) chain	COL11A1	0.00055
P13686	Tartrate-resistant acid phosphatase type 5	ACP5	0.00014
P14207	Folate receptor beta	FOLR2	0.00001
P16278	Beta-galactosidase	GLB1	0.00223
P18084	Integrin beta-5	ITGB5	0.00003
P22105	Tenascin-X	TNXB	0.00018
P22692	Insulin-like growth factor-binding protein 4	IGFBP4	0.00017
P22891	Vitamin K-dependent protein Z	PROZ	0.00004
P23083	Immunoglobulin heavy variable 1–2	IGHV1-2	0.00012
P26927	Immunoglobulin heavy variable 1–2	MST1	0.00001
P29373	Cellular retinoic acid-binding protein 2	CRABP2	0.00000
P32455	Guanylate-binding protein 1	GBP1	0.01012
P35442	Thrombospondin-2	THBS2	0.00832

FC, fold change; P/C, protein abundance in patient/protein abundance in control.

**Table 2:** TOP20 downregulation in differentially expressed proteins in sclerotic group versus samples from FNF group.

Accession	Protein name	Gene name	P value
O00194	Ras-related protein Rab-27B	RAB27B	0.00002
O00602	Ficolin-1	FCN1	0.00002
O15067	Phosphoribosylformylglycinamidine synthase	PFAS	0.00010
O75594	Peptidoglycan recognition protein 1	PGLYRP1	0.00018
O94903	Pyridoxal phosphate homeostasis protein	PLPBP	0.00076
O94919	Endonuclease domain-containing 1 protein	ENDOD1	0.00017
O95777	U6 snRNA-associated Sm-like protein LSm8	LSM8	0.00090
P01116	GTPase KRas	KRAS	0.00002
P05089	Arginase-1	ARG1	0.00002
P10153	Non-secretory ribonuclease	RNASE2	0.00000
P11279	Lysosome-associated membrane glycoprotein 1	LAMP1	0.00111
P12829	Myosin light chain 4	MYL4	0.00048
P16403	Histone H1.2	H1-2	0.00040
P21815	Bone sialoprotein 2	IBSP	0.00001
P25815	Protein S100-P	S100P	0.00022
P26583	High mobility group protein B2	HMGB2	0.00225
P31997	Carcinoembryonic antigen-related cell adhesion molecule 8	CEACAM8	0.00008
P32320	Cytidine deaminase	CDA	0.00001
P39748	Flap endonuclease 1	FEN1	0.00007
P50148	Guanine nucleotide-binding protein G(q) subunit alpha	GNAQ	0.00004

FC, fold change; P/C, protein abundance in patient/protein abundance in control.

**Table 3:** TOP20 upregulation in differentially expressed proteins in FHN group versus samples from FNF group.

Accession	Protein name	Gene name	P value
P01614	Immunoglobulin kappa variable 2D-40	IGKV2D-40	0.00003
A0A0A0MT36	Immunoglobulin kappa variable 6D-21	IGKV6D-21	0.00002
A0A0B4J1V2	Immunoglobulin heavy variable 2–26	IGHV2-26	0.00015
A0A0B4J1V6	Immunoglobulin heavy variable 3–73	IGHV3-73	0.00001
A0A0B4J1X8	Immunoglobulin heavy variable 3–43	IGHV3-43	0.00001
A0A0C4DH36	Probable non-functional immunoglobulin heavy variable 3–38	IGHV3-38	0.00001
A0A0C4DH38	Immunoglobulin heavy variable 5–51	IGHV5-51	0.00000
A0AVT1	Ubiquitin-like modifier-activating enzyme 6	UBA6	0.00115
O00391	Sulfhydryl oxidase 1	QSOX1	0.00008
O00534	von Willebrand factor A domain-containing protein 5A	VWA5A	0.00001
O00567	Nucleolar protein 56	NOP56	0.00000
O14498	Immunoglobulin superfamily containing leucine-rich repeat protein	ISLR	0.00172
O15162	Phospholipid scramblase 1	PLSCR1	0.00001
O15305	Phosphomannomutase 2	PMM2	0.00000
O15460	Prolyl 4-hydroxylase subunit alpha-2	P4HA2	0.00011
O43237	Cytoplasmic dynein 1 light intermediate chain 2	DYNC1LI2	0.00010
O43252	Bifunctional 3′-phosphoadenosine 5′-phosphosulfate synthase 1	PAPSS1	0.00001
Q96HN2	Adenosylhomocysteinase 3	AHCYL2	0.00000
O60271	C-Jun-amino-terminal kinase-interacting protein 4	SPAG9	0.00001
O60488	Long-chain-fatty-acid–CoA ligase 4	ACSL4	0.00000

FC, fold change; P/C, protein abundance in patient/protein abundance in control.

**Table 4:** TOP20 downregulation in differentially expressed proteins in FHN group versus samples from FNF group.

Accession	Protein name	Gene name	P value
A0A0B4J1Y8	Immunoglobulin lambda variable 9–49	IGLV9-49	0.00000
B2RUZ4	Small integral membrane protein 1	SMIM1	0.00125
O00194	Ras-related protein Rab-27B	RAB27B	0.00002
O00602	Ficolin-1	FCN1	0.00002
O94919	Endonuclease domain-containing 1 protein	ENDOD1	0.00017
O95777	U6 snRNA-associated Sm-like protein LSm8	LSM8	0.00090
P02775	Platelet basic protein	PPBP	0.00210
P05089	Arginase-1	ARG1	0.00002
P07451	Carbonic anhydrase 3	CA3	0.00118
P08833	Insulin-like growth factor-binding protein 1	IGFBP1	0.00004
P09105	Hemoglobin subunit theta-1	HBQ1	0.00005
P10153	Non-secretory ribonuclease	RNASE2	0.00000
P11279	Lysosome-associated membrane glycoprotein 1	LAMP1	0.00111
P11678	Eosinophil peroxidase	EPX	0.00001
P12829	Myosin light chain 4	MYL4	0.00048
P14555	Phospholipase A2, membrane associated	PLA2G2A	0.00010
P20742	Pregnancy zone protein	PZP	0.00052
P28289	Tropomodulin-1	TMOD1	0.00017
P29972	Aquaporin-1	AQP1	0.00000
P30613	Pyruvate kinase PKLR	PKLR	0.00001

FC, fold change; P/C, protein abundance in patient/protein abundance in control.

**Table 5:** TOP20 upregulation in differentially expressed proteins in sclerosis group versus samples from FHN group.

Accession	Protein name	Gene name	P value
P01614	Immunoglobulin kappa variable 2D-40	IGKV2D-40	0.00005
A0A0A0MT36	Immunoglobulin kappa variable 6D-21	IGKV6D-21	0.00004
A0A0B4J1V2	Immunoglobulin heavy variable 2–26	IGHV2-26	0.00032
A0A0B4J1V6	Immunoglobulin heavy variable 3–73	IGHV3-73	0.00001
A0A0C4DH36	Probable non-functional immunoglobulin heavy variable 3–38	IGHV3-38	0.00002
O14879	Interferon-induced protein with tetratricopeptide repeats 3	IFIT3	0.00037
O15162	Phospholipid scramblase 1	PLSCR1	0.00002
O75165	DnaJ homolog subfamily C member 13	DNAJC13	0.00034
O75594	Peptidoglycan recognition protein 1	PGLYRP1	0.00024
O75923	Dysferlin	DYSF	0.00082
O94903	Pyridoxal phosphate homeostasis protein	PLPBP	0.00005
O95232	Luc7-like protein 3	LUC7L3	0.00000
O95466	Formin-like protein 1	FMNL1	0.00004
P00973	2′-5′-oligoadenylate synthase 1	OAS1	0.00071
P01703	Immunoglobulin lambda variable 1–40	IGLV1-40	0.00001
P01742	Immunoglobulin heavy variable 1–69	IGHV1-69	0.00000
P06280	Alpha-galactosidase A	GLA	0.00005
P07093	Glia-derived nexin	SERPINE2	0.00022
P08575	Receptor-type tyrosine-protein phosphatase C	PTPRC	0.00034
P08631	Tyrosine-protein kinase HCK	HCK	0.00000

FC, fold change; P/C, protein abundance in patient/protein abundance in control.

**Table 6:** TOP20 downregulation in differentially expressed proteins in sclerosis group versus samples from FHN group.

Accession	Protein name	Gene name	P value
A0A0B4J1Y8	Immunoglobulin lambda variable 9–49	IGLV9-49	0.00008
P01344	Insulin-like growth factor II	IGF2	0.00046
P02775	Platelet basic protein	PPBP	0.00325
P05154	Plasma serine protease inhibitor	SERPINA5	0.00269
P07451	Carbonic anhydrase 3	CA3	0.00012
P14555	Phospholipase A2, membrane associated	PLA2G2A	0.00000
P20742	Pregnancy zone protein	PZP	0.00411
P22891	Vitamin K-dependent protein Z	PROZ	0.00009
P28289	Tropomodulin-1	TMOD1	0.00005
P30613	Pyruvate kinase PKLR	PKLR	0.00021
P30711	Glutathione S-transferase theta-1	GSTT1	0.00029
P55290	Cadherin-13	CDH13	0.00185
P62318	Small nuclear ribonucleoprotein Sm D3	SNRPD3	0.00006
P79483	HLA class II histocompatibility antigen, DR beta 3 chain	HLA-DRB3	0.00000
Q5ZPR3	CD276 antigen	CD276	0.00002
Q92905	COP9 signalosome complex subunit 5	COPS5	0.00044
Q99627	COP9 signalosome complex subunit 8	COPS8	0.00000
Q9H479	Fructosamine-3-kinase	FN3K	0.00018
Q9HBI1	Beta-parvin	PARVB	0.00005
P22105	Tenascin-X	TNXB	0.00425

FC, fold change; P/C, protein abundance in patient/protein abundance in control.

### GO function enrichment analysis

**Figure 3:**
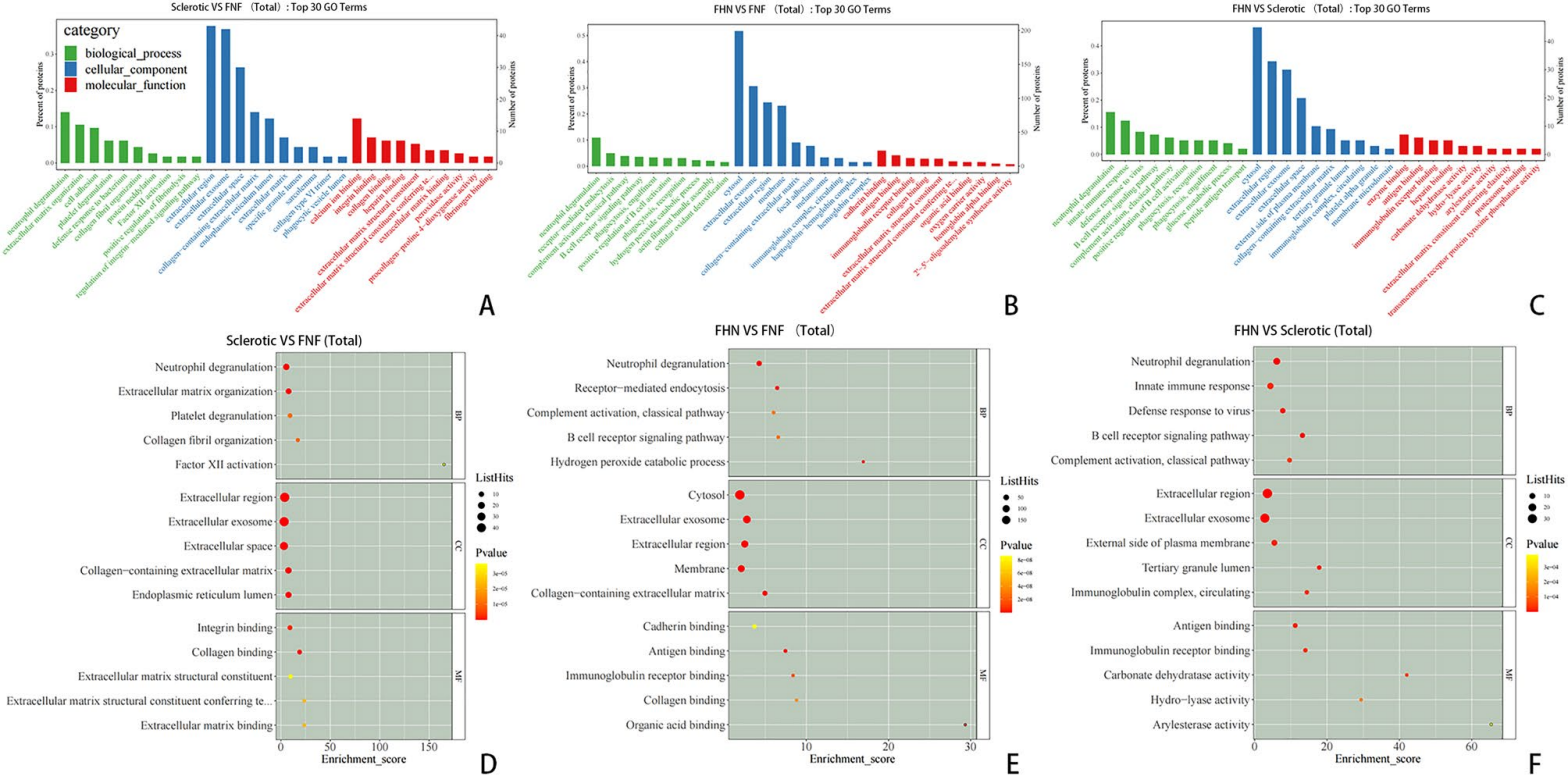
Top Gene Ontology (GO) terms identified in pairwise comparisons between sclerotic, FNF, and FHN samples. (**A**–**C**) Bar charts of the three groups. The top 10 GO terms with a differential protein count greater than 1 in each category were selected and sorted in descending order of − log10P-value. The x-axis represents the GO term name, and the y-axis represents the protein count and its percentage. (**D**–**F**) Bubble plots of the three groups. The top 5 GO terms with a differential protein count greater than 1 in each category were selected and sorted in descending order of − log10P-value. The x-axis represents the enrichment score, and the y-axis represents the top 5 term information for BP/CC/MF, respectively. Larger bubbles indicate a greater number of proteins, and the color of the bubbles changes from yellow to red, indicating a smaller p-value and greater significance.

GO functional enrichment analysis between the sclerotic and FNF groups showed that most proteins were present in the extracellular region (Fig. 3). The top five biological processes identified included extracellular matrix organization, cell adhesion, collagen fibril organization, receptor-mediated endocytosis, and positive regulation of B cell activation. The main molecular functions suggested that the DEPs were involved in calcium ion, collagen, and integrin binding, and extracellular matrix structural formation. GO functional enrichment analysis of the FHN and FNF groups showed that most proteins were enriched in the cytosol. The analysis identified the top five biological processes involved in neutrophil degranulation, proteolysis, receptor-mediated endocytosis, complement activation, classical pathways, and defense responses to viruses. The main molecular functions suggested that the DEPs were related to identical proteins, ATP, cadherin, antigen, and immunoglobulin receptor binding. GO functional enrichment analysis of the sclerotic and FHN groups also showed that most proteins were present in the cytosol. This analysis identified the top five biological processes that were involved in bicarbonate transport, one-carbon metabolic processes, post-translational protein modification, protein deneddylation, nucleotide excision repair, and DNA damage recognition. The main molecular functions suggested that the DEPs were related to carbonate dehydratase, hydrolase, arylesterase, kinase, and serine-type endopeptidase inhibitor activity. For each comparison group, we selected DEPs with counts greater than three and less than 50. The top six entries were sorted according to their corresponding −log10 P-value. The relationships between the selected GO terms and corresponding list of DEPs were visualized using a chord diagram for GO enrichment analysis (Figs. S3–5).

### KEGG pathway enrichment and PPI analysis

**Figure 4:**
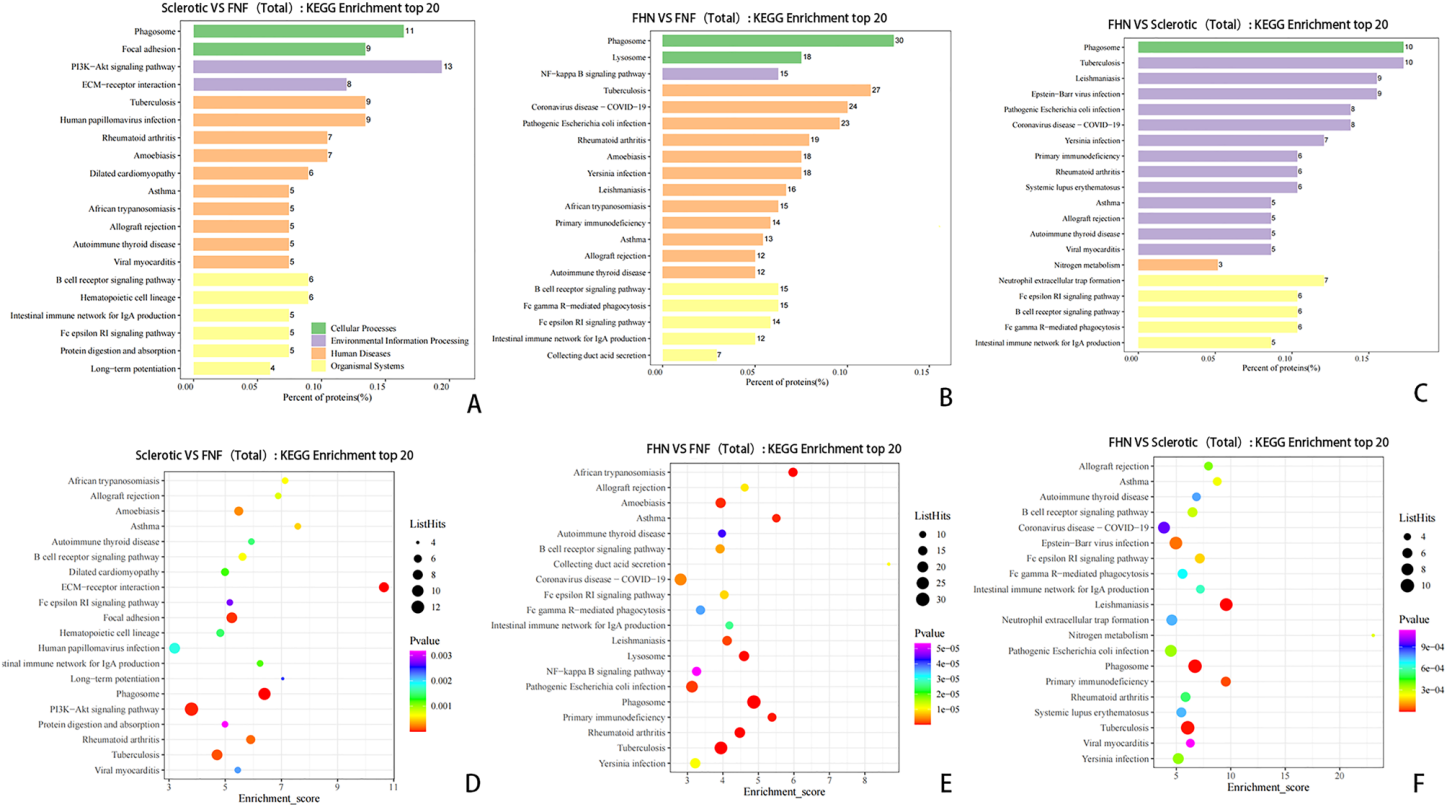
Top KEGG pathways identified in pairwise comparisons between sclerotic, FNF, and FHN samples. (**A**–**C**) Horizontal distribution plots of differential proteins in KEGG pathway enrichment analysis. The x-axis represents the ratio (%) of differentially expressed proteins annotated to each metabolic pathway compared to the total number of differentially expressed proteins annotated to KEGG pathways. The y-axis represents the pathway names, and the numbers on the right side of the bars represent the number of differentially expressed proteins annotated to each pathway. Different colors of the bars represent different information. (**D**–**F**) Bubble plots of differential proteins in KEGG pathway enrichment analysis. The x-axis represents the enrichment score, and the y-axis represents the top 20 pathway information. Larger bubbles indicate a greater number of differential proteins, and the color of the bubbles changes from red to green to blue to purple, indicating a smaller p-value and greater significance.

KEGG pathway enrichment analysis indicated that numerous co-expression pathways were significantly associated with perinatal sclerosis and femoral head collapse. The top three pathways for protein and metabolite expression between the sclerosis and FNF groups were the phosphatidylinositol 3-kinase (PI3K)-Akt signaling pathway, phagosome, tuberculosis, glycerophospholipid metabolism, choline metabolism in cancer, and retrograde endocannabinoid signaling. The top three pathways for protein and metabolite expression in the FHN and FNF groups were phagosomes, tuberculosis, pathogenic *Escherichia coli* infection, glycerophospholipid metabolism, choline metabolism in cancer, and retrograde endocannabinoid signaling. The top three pathways for protein and metabolite expression in the sclerosis and FHN groups were nitrogen metabolism, focal adhesion, adhesion of cell molecules, choline metabolism in cancer, retrograde endocannabinoid signaling, and linoleic acid metabolism (Fig. 4)^19^.

**Figure 5:**
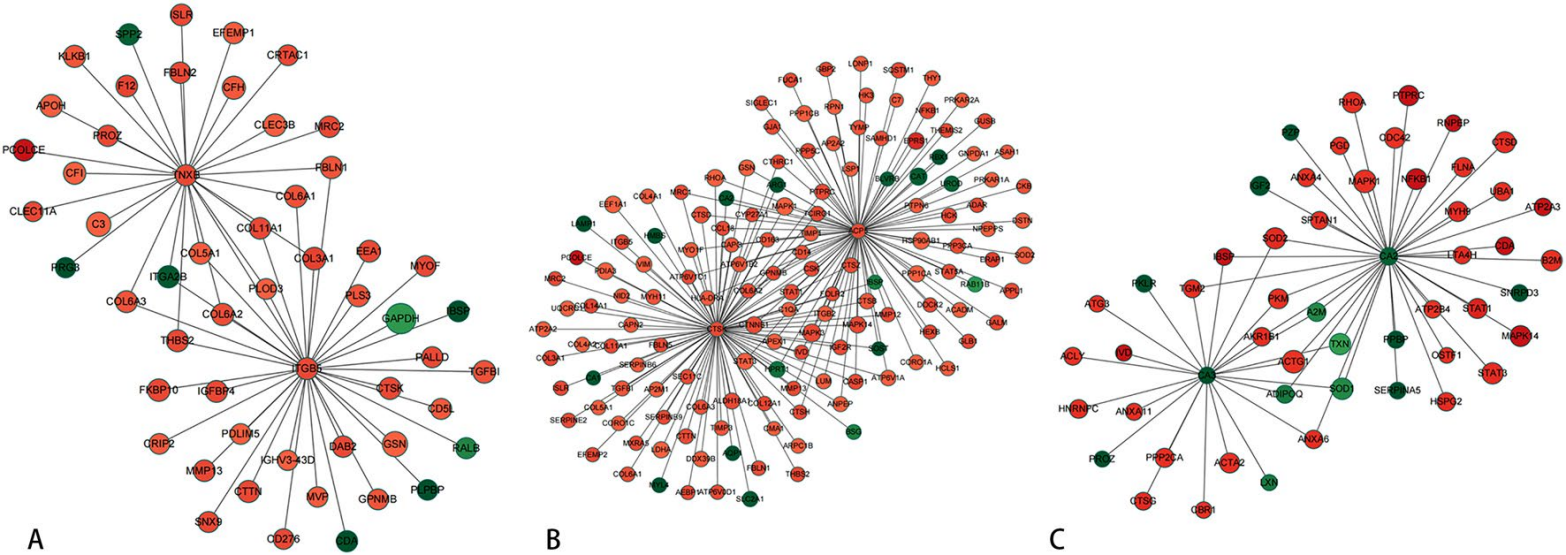
PPI networks of key proteins in pairwise comparisons between the three groups. Visualization of key proteins with high connectivity and clinical significance. The circles represent differentially expressed proteins/genes, with red indicating upregulated proteins/genes and green indicating downregulated proteins/genes. The size of the circles represents the degree of connectivity, with larger circles indicating higher connectivity.

PPI network analysis was conducted using the STRING database. As depicted in Fig. 5, the DEPs were closely interconnected, with integrin beta-5 (P18084), tenascin-X (P22105), tartrate-resistant acid phosphatase type 5 (P13686), cathepsin K (P43235), carbonic anhydrase 2 (P00918), and carbonic anhydrase 3 (P07451) playing pivotal roles in regulating differential protein expression.

### Western blot

**Figure 6:**
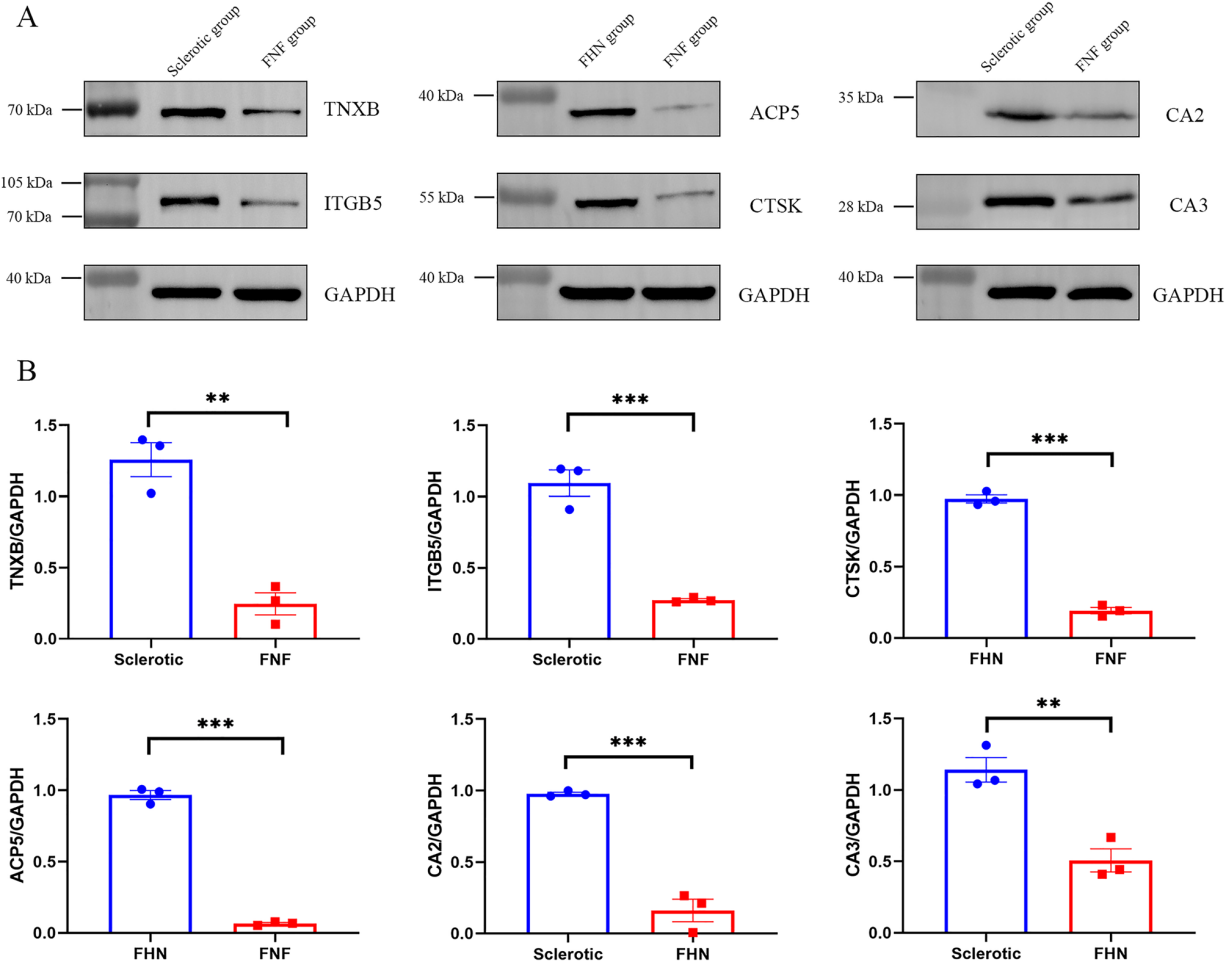
Western blot validation of proteomic data. (**A**) Elevated levels of *ITGB5, TNXB*, CA II, CA III were observed in the peri-implant sclerosis samples, while elevated levels of *ACP5* and CTSK were observed in femoral head necrosis samples. (**B**) The ratio of TIGAR/GAPDH intensities in Western blot. GAPDH was used as a loading control, quantified and normalized to GAPDH using ImageJ. All experiments were performed in triplicate, * indicates significant expression level change compared to the control group, *P < 0.05, **P < 0.01. ***P < 0.001.

Based on the identified DEPs, six key proteins were selected through log-FC values and relevant literature review for western blot analysis to verify their expression levels across the three groups. Western blotting was conducted to confirm the expression of the selected proteins. The results of the western blot showed that compared with the FNF group, the proteins *TNXB* and *ITGB5* exhibited increased expression in sclerosis around screw paths samples; compared with FNF group, the proteins ACP5 and CTSK were upregulated in FHN samples; and compared with the FHN group, and the protein expressions of CA II and CA III were upregulated in sclerosis around screw path samples (Fig. 6). Western blot data generally agreed with the proteomic data on the three groups. These results underscored the importance and applicability of the selected hub proteins in predicting sclerosis around the screw path and FHN in clinical processes.

### Metabolomic profile analysis

**Figure 7:**
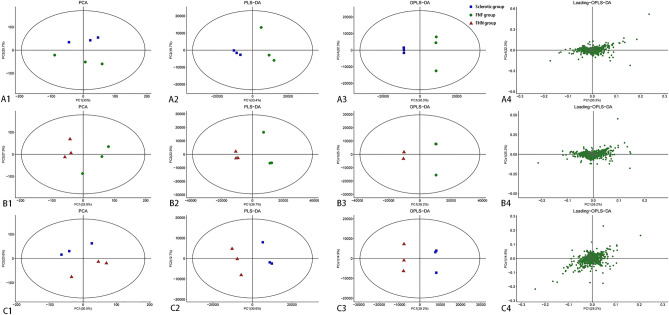
Multivariate statistical analysis of pairwise metabolite comparisons between the three groups. (**A1**–**C1**) Principal component analysis (PCA). If the difference between two samples is significant, the coordinate points on the score plot will be relatively far apart, and vice versa. The ellipse region represents a 95% confidence interval. (**A2**–**C2**) Partial least squares-discriminant analysis (PLS-DA). R2Y (cum) and Q2 (cum) represent the explanatory and predictive abilities of the PLS-DA model, respectively. Values closer to 1 indicate better model performance in explaining and predicting differences between the two groups. (**A3**–**C3**) Orthogonal partial least squares-discriminant analysis (OPLS-DA). The t1 component reflects the maximum inter-group differences, allowing direct differentiation of inter-group variations, while the orthogonal components reflect intra-group variations. (**A4**–**C4**) Loadings plots to identify the influence strength of metabolites on the comparative groups. The loadings range can be − 1 to 1. Loadings close to − 1 or 1 indicate a strong influence of the variable on the component, while loadings close to 0 indicate a weak influence of the variable on the component.

In total, 2972 metabolites were detected using LC–MS. As depicted in Tables S5–7, between the sclerosis and FNF groups, 28 upregulated and 16 downregulated metabolites were identified. Between the FHN and FNF groups, 51 upregulated and 43 downregulated metabolites were identified. Furthermore, 14 upregulated and 17 downregulated metabolites were identified in the FHN and sclerosis groups, respectively. PCA of the metabolites from the three groups was performed, and loading plots were constructed to assess the potential variation among the samples (Figs. 7, S6). To illustrate the relationship between samples and variation in the expression of metabolites among samples, hierarchical clustering was conducted on significantly different metabolite expression levels. The results are shown in Figs. S7–9. Volcano plots were used to visualize the p-values, variable importance in projection, and FC values, aiding in the selection of differentially expressed metabolites, as depicted in Fig. S10. Correlation analysis was conducted to assess the close relationship between significantly different metabolites and gain further insight into the interactions among metabolites during biological state changes. The results of the correlation analysis are shown in Fig. S11.

**Figure 8:**
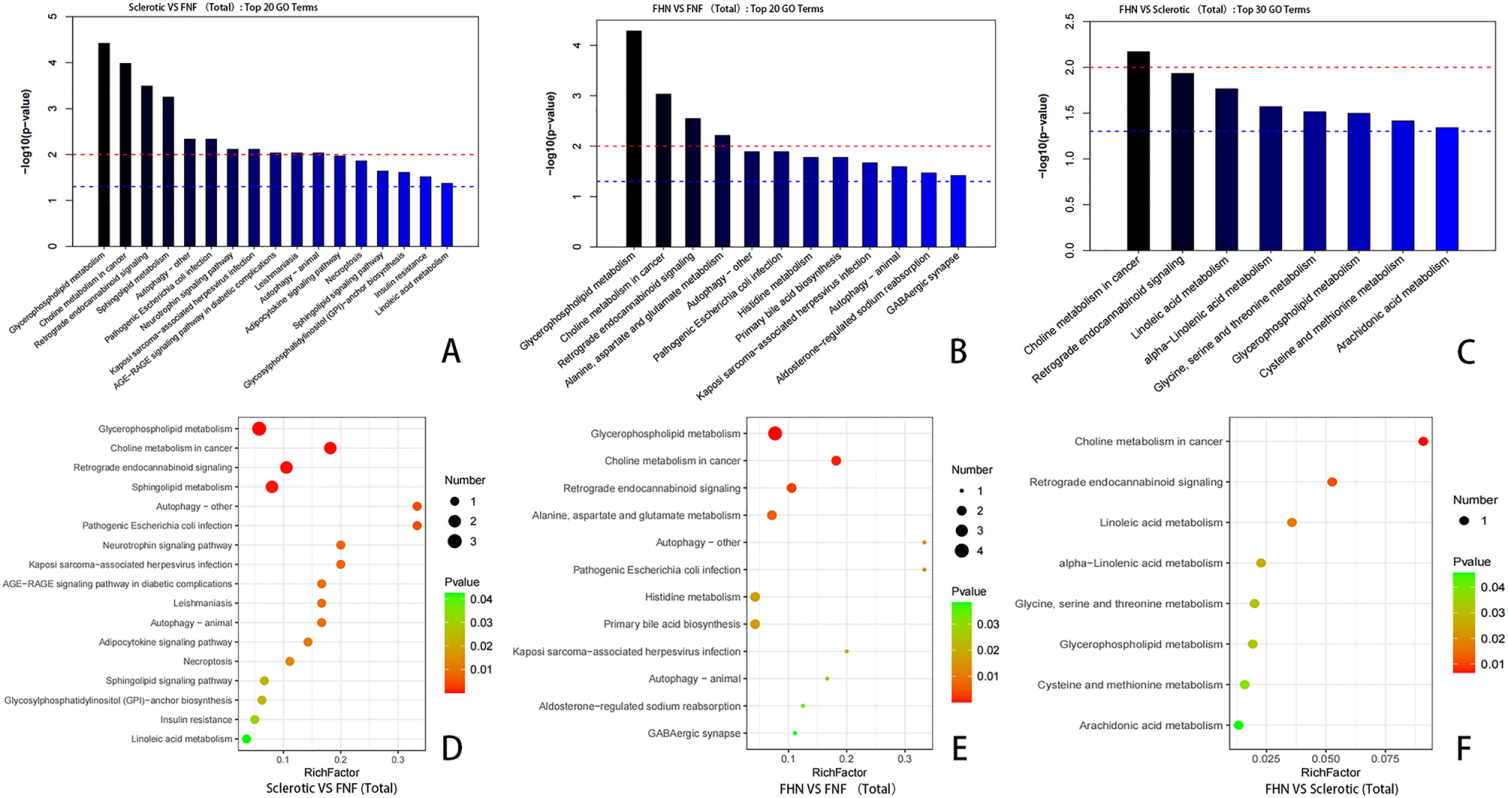
KEGG pathway enrichment analysis of metabolites between the three groups. (**A**–**C**) Top enriched metabolic pathway plots. The p-value in each metabolic pathway indicates the significance of pathway enrichment. The red line indicates a p-value of 0.01, and the blue line indicates a p-value of 0.05. When the height of the bar exceeds the blue line, the signaling pathway represented by the bar is considered significant. (**D**–**F**) Bubble plots of enriched pathways. The y-axis represents the pathway names, and the x-axis represents the enrichment factor (Rich factor = number of significantly different metabolites/total number of metabolites in that pathway). A larger Rich factor indicates a greater degree of enrichment. The color of the bubbles changes from green to red, indicating a decreasing p-value. The size of the bubbles indicates the number of metabolites enriched in the pathway.

Metabolic pathway enrichment analysis of differential metabolites was conducted using the KEGG. Significantly enriched pathways were selected to generate the bubble plots. The comparison between the sclerosis and FNF groups included 17 key metabolic pathways, with the major metabolites involved in glycerophospholipid metabolism, choline metabolism in cancer, and retrograde endocannabinoid signaling. The analysis of the necrosis and fracture groups included 12 key metabolic pathways, with major metabolites involved in glycerophospholipid metabolism, choline metabolism in cancer, and alanine, aspartate, and glutamate metabolism. Analysis of the FHN and sclerosis groups included eight key metabolic pathways, with major metabolites involved in linoleic acid metabolism, alpha-linolenic acid metabolism, and glycine, serine, and threonine metabolism (Fig. 8).

## Discussion

The formation of sclerosis around screw paths is a complex process regulated by proteins, metabolites, and signaling pathways. Using a comprehensive analysis of the quantitative proteome and metabolome, we detected 2563 proteins and 2972 metabolites. KGML network analysis was conducted on metabolites and proteins, and DEPs associated with DEMs were mapped onto the KEGG pathway database. Upon obtaining their common pathway information, correlation heatmaps (Fig. S12) and associative network diagrams (Figs. S13–15) indicated that the proteins and metabolites correlated across both omics did not map to the pathways associated with osteogenesis and necrosis of the femoral head. This study’s approach to understanding the significance of glycerophospholipid and choline metabolism in bone health and femoral head necrosis, along with its potential impact on therapeutic interventions, marks a significant contribution to our comprehension of femoral head diseases. Moreover, perinatal sclerosis and aseptic necrosis of the femoral head induced differential expression of numerous genes and proteins. These DEPs and DEMs may serve as key biomarkers of sclerosis.

Integrins are pivotal in mediating the response of bone cells to mechanical loading, closely interacting with the PI3K-Akt signaling pathway. The process begins when mechanical strain facilitates the assembly of integrin-associated focal adhesion molecules. This assembly activates the focal adhesion kinase and Src pathways, which in turn initiates the PI3K and mitogen-activated protein kinase pathways^20^. Composed of α and β dimers, integrins experience conformational changes in the β subunit under fluid flow shear stress (FFSS), triggering a cascade of downstream signaling events^21,22^. Research by Kim et al. highlighted the role of integrin αV/β5 as a receptor for irisin in bone cells, paving the way for further exploration of downstream signaling pathways and stress responses near screw paths^23^. This interplay between integrin receptors and extracellular matrix proteins forms a crucial bridge, translating skeletal load into cellular and biochemical activities within bone cells.

Furthermore, cyclic mechanical stimulation of integrins induces alterations in the membrane potential of human bone cells, leading to cytoskeletal disruption and inhibition of tyrosine kinase activity^24^. Membrane depolarization, which involves tetrodotoxin-sensitive sodium channels, can be modulated by antibodies targeting αV, β1, and β5 integrins^25^. Recent research underscores the significance of integrins as essential mechanosensors and modulators of Insulin-like growth factor 1 (IGF-I) signaling in osteoblasts, with calcium and Cyclic adenosine monophosphate (cAMP) signaling pathways serving as vital mediators^26,27^. In both hardening and fractured groups, pathways related to calcium-binding and cAMP were notably enriched, with IGFBP4 identified as one of the DEPs. These insights further highlight the critical roles of integrins and the PI3K-Akt signaling pathway in orchestrating the complex response of bone cells to mechanical stresses, emphasizing their importance in bone health and injury recovery.

Recent investigations have highlighted the pivotal roles of carbonic anhydrase II (CA II), regulated by SOST/sclerostin, in maintaining mineral homeostasis^28^. CA II expression escalates in tandem with the accumulation of calcium deposits, underscoring its vital role in the crucial stages of bone tissue formation, development, remodeling, and repair^29^. Situated on the cell membrane as a cytoplasmic enzyme, CA II sees an upregulation in expression when exposed to bicarbonate^30,31^. In our proteomic analysis focusing on the hardening and necrosis groups, carbonate dehydratase activity emerged as the most significantly enriched molecular function in the GO enrichment analysis. Cells that express CA II are proficient in synthesizing precipitates enriched with calcium ions, a process that can be inhibited by the specific CA II inhibitor, acetazolamide. Although traditionally associated with bone resorption, CA, especially CAII, has also been implicated in bone formation according to recent studies^32^. Shifting our attention to carbonic anhydrase III (CA III), utilizing single-cell cloning technology, we identified the gene encoding CA III, associated with high SOST/sclerostin expression, as a novel marker for differentiated osteoblasts. CAIII expression in osteoblasts proliferates as osteoblasts/osteocytes differentiate. It is regulated by parathyroid hormone in vitro and in vivo, and it confers protection to osteocytes from oxidative stress and death induced by hydrogen peroxide^33^.

CA III is highly abundant in the slow skeletal muscle (10%), adipocytes (24%), and liver (8%)^33^. Despite its prevalence in these tissues, the precise physiological roles of CA III are not fully understood. The activities attributed to CA III include maintaining intracellular pH hydration enzyme activity, safeguarding muscles from oxidative stress, antioxidative activity in oxidative phosphorylation, and playing a role in energy metabolism^34^. We hypothesize that as osteocytes become embedded in mineralized bone, they face increasingly hypoxic conditions, which is counteracted by the augmented expression of CA III. CA II and CAIII are integral to the functioning of osteocytes by regulating mineral balance and providing protection during osteocyte differentiation. CA II engages in bone formation and resorption, whereas CA III shields osteocytes from oxidative stress. These proteins are crucial to the physiological processes of osteocytes, unveiling new directions for future research into bone metabolism and treatments for bone diseases.

In this study, a proteomics-based KEGG enrichment analysis underscored the notable enrichment pathways, including phagosome, lysosome, NF-kappa B signaling pathway, and osteoclast differentiation, particularly pronounced in the FHN group compared to the FNF group. Within these enriched pathways, DEPs such as *acid phosphatase 5 (ACP5)* and cathepsin K (CTSK) were identified as playing pivotal roles. CTSK, recognized as a cysteine protease secreted by osteoclasts, plays a critical role in the bone resorption process^35^. The functioning of osteoclasts is closely linked to the levels of tartrate-resistant acid phosphatase 5b, which can serve as a direct indicator of their activity^36^. In an exploratory study, Chen et al. delved into the fluctuations in reactive oxygen species levels and their impact on osteoclast behavior in both patients with hormonal FHN and a rat model for osteonecrosis of the femoral head (ONFH). Their findings revealed a pronounced upregulation of osteoclast-specific genes, including CTSK and *ACP5*, within the necrotic zones as opposed to the healthy tissue areas^37^. Subsequent in vivo investigations noted increased CTSK expression within the rabbit group affected by steroid-associated FHN. Counteracting the upsurge of CTSK expression in the necrotic femoral heads was shown to effectively curb receptor activator of nuclear factor kappa-B ligand (RANKL)-induced osteoclast differentiation while simultaneously fostering osteoblast formation^38^. This highlights the significant contribution of proteins such as CTSK and *ACP5* in osteoclasts differentiation and bone resorption, offering insightful avenues for therapeutic intervention strategies. Biomarkers in patients with femoral neck fractures (FNF) are rarely detected through proteomic analysis. Liu et al. analyzed the serum proteome of 32 patients with femoral head necrosis (FHN) and 24 healthy controls, identifying fibrinogen alpha chain (FGA) as a novel potential biomarker highly expressed in FHN patients^39^. Yang et al.'s proteomic analysis revealed a significant reduction in Type V collagen alpha 2 chain (COL5A2) in the necrotic regions of FHN patients^40^. However, combining transcriptomic and proteomic approaches, Yang et al. examined six patients with steroid-induced FHN and six with femoral neck fractures, identifying LRG1, Serpine2, STMN1, COL14A1, SLC37A2, and MMP2 as key genes/proteins in FHN^41^. Consequently, this study demonstrates that the targets and conclusions derived from the combined use of proteomics and metabolomics in analyzing FHN patient samples vary significantly.

Lipid metabolism, notably in the realms of glycerophospholipid and choline, emerges as a pivotal metabolic pathway linked with ovariectomy-induced postmenopausal osteoporosis^42^. Observations reveal that metabolites within these pathways orchestrate a complex network of interactions, culminating in the suppression of amino acids and glycerophospholipids synthesis. This suppression potentially disrupts the equilibrium between osteoblasts and osteoclasts, consequently impairing bone health^43,44^. Moreover, an association has been noted between the cellular content of glycerophospholipids and osteoclastogenesis, with an uptick observed during osteoclast differentiation. These findings highlight the crucial role glycerophospholipid metabolism plays in preserving skeletal integrity^45^. In the context of femoral head osteonecrosis, metabolomic investigations have pinpointed glycerophospholipid metabolism as a significant pathway implicated in the disease process^46^. Statin medications have shown efficacy in treating steroid-induced ischemic necrosis of the femoral head, potentially by ameliorating dysregulated glycerophospholipid metabolism^47,48^. However, the significance of choline in bone metabolism warrants attention. Phosphocholine, a specific substrate of PHOSPHO1, is crucial for generating inorganic phosphate essential for bone mineralization^49^. A reduction in choline kinase activity could diminish the mineralization efficacy of inorganic phosphate, suggesting choline kinase’s dual regulatory impact on osteoclasts and osteoblasts^50^. Although CDP-choline supplementation did not fully restore bone mass in vivo, it mitigated osteoclast-mediated effects in Flp/Flp mice. Considering the approval of CDP-choline for human use, this discovery opens avenues for investigating the influence of choline on bone microstructure and necessitates further research^7^. The metabolomics of this study findings indicate that glycerophospholipid and choline metabolism are the most significantly enriched pathways in comparisons between the sclerosis and fracture groups, as well as the necrosis and fracture groups, highlighting their essential roles in bone metabolism and femoral head osteonecrosis. This provides evidence for a potential link between sclerosis around screw paths and FHN, aligning with our prior research^51^. Nevertheless, additional studies are essential to decode the exact mechanisms through which these metabolic pathways impact bone metabolism and femoral head osteonecrosis and to identify viable therapeutic targets.

This study has its not without limitations. First of all, the ages of the participants in the three groups did not match exactly, and a larger sample analysis with age matching is needed in the future to address solve this problem. Obtaining femoral head samples required informed consent from eligible patients undergoing total hip arthroplasty. Owing to the nature of fractures and challenges in sampling, it was not feasible to collect normal hip joint femoral specimens from adults, making precise age matching between the two groups difficult to achieve. Second, the impact of FNF samples on protein expression may have affected the study’s accuracy. To mitigate this potential effect, all control samples were collected from the patients within 24 h after traumatic fractures. However, to achieve this clinical goal, appropriate in vitro and in vivo experiments, as well as the analysis of sufficient clinical samples, are still required before there is a sufficient biological basis to promote the use of relevant targets and circulating markers in clinical practice.

## Conclusion

In conclusion, this study represents a comprehensive and extensive comparative analysis of the proteomic and metabolic expression profiles associated with femoral head samples in cases of sclerosis around screws, FNFs, and avascular necrosis of the femoral head. Our investigation has identified pathways involving DEPs and DEMs associated with ONFH and sclerosis around the screw. These findings hold promise to expand and deepen our understanding of novel predictive biomarkers and potentially effective therapeutic strategies for hormone- and stress-induced avascular necrosis of the femoral head. Additionally, by elucidating these pathways, our study adds to the understanding of femoral head diseases, providing the potential for interventions.

### Supplementary Information

Supplementary Figure S1.

Supplementary Figure S2.

Supplementary Figure S3.

Supplementary Figure S4.

Supplementary Figure S5.

Supplementary Figure S6.

Supplementary Figure S7.

Supplementary Figure S8.

Supplementary Figure S9.

Supplementary Figure S10.

Supplementary Figure S11.

Supplementary Figure S12.

Supplementary Figure S13.

Supplementary Figure S14.

Supplementary Figure S15.

Supplementary Legends.

Supplementary Information.

Supplementary Tables.

## Data Availability

The datasets used and/or analyzed during the current study available from the corresponding author or public database. The experimental data of metabolomics has been uploaded to the GSA database (Project ID: PRJCA025591; Submission ID: subPRO037930, https://ngdc.cncb.ac.cn/omix/preview/LBvGaxqN). The experimental data of proteomics has been uploaded to the iProX database (Project ID: IPX0008705000; ProteomeXchange ID: PXD051808).

## References

[1] Rajfer RA, Carlson BA, Johnson JP (2024). High-energy femoral neck fractures in young patients. J. Am. Acad. Orthop. Surg..

[2] Tian F, Zhang L, Zhao H, Liang C, Zhang N, Song H (2014). An increase in the incidence of hip fractures in Tangshan, China. Osteoporos. Int..

[3] Yang J, Lin L, Chao K, Chuang S, Wu C, Yeh T, Lian Y (2013). Risk factors for nonunion in patients with intracapsular femoral neck fractures treated with three cannulated screws placed in either a triangle or an inverted triangle configuration. J. Bone Joint Surg..

[4] Bhandari M, Swiontkowski M (2017). Management of acute hip fracture. N. Engl. J. Med..

[5] Patterson J, Ishii K, Tornetta P, Leighton R, Friess D, Jones C, Levine A, Maclean J, Miclau T, Mullis B, Obremskey W, Ostrum R, Reid J, Ruder J, Saleh A, Schmidt A, Teague D, Tsismenakis A, Westberg J, Morshed S (2020). Open reduction is associated with greater hazard of early reoperation after internal fixation of displaced femoral neck fractures in adults 18–65 years. J. Orthop. Trauma.

[6] Nyholm A, Palm H, Sandholdt H, Troelsen A, Gromov K (2020). Risk of reoperation within 12 months following osteosynthesis of a displaced femoral neck fracture is linked mainly to initial fracture displacement while risk of death may be linked to bone quality: A cohort study from Danish Fracture Database. Acta Orthop..

[7] Liu Y, Liang H, Zhou X, Song W, Shao H, He Y, Yang Y, Guo L, Li P, Wei X, Duan W (2022). Micro-computed tomography analysis of femoral head necrosis after long-term internal fixation for femoral neck fracture. Orthop. Surg..

[8] Liu Y, Song W, Liang H, Li C, Niu W, Shao H, Wang Y, Yang Z, Li P, Wu X, He Y, Wei X, Duan W (2022). Comparison of femoral mechanics before and after internal fixation removal and the effect of sclerosis on femoral stress: A finite element analysis. BMC Musculoskelet. Disord..

[9] Konarski W, Poboży T, Konarska K, Śliwczyński A, Kotela I, Hordowicz M, Krakowiak J (2023). Osteonecrosis related to steroid and alcohol use-an update on pathogenesis. Healthcare.

[10] Mont M, Cherian J, Sierra R, Jones L, Lieberman J (2015). Nontraumatic osteonecrosis of the femoral head: Where do we stand today? A ten-year update. J. Bone Joint Surg..

[11] Xu Y, Ritchie SC, Liang Y, Timmers PRHJ (2023). An atlas of genetic scores to predict multi-omic traits. Nature.

[12] Luo L, Ma W, Liang K, Wang Y, Su J, Liu R, Liu T (2023). Spatial metabolomics reveals skeletal myofiber subtypes. Sci. Adv..

[13] Demichev V, Szyrwiel L, Yu F, Teo GC, Rosenberger G, Niewienda A, Ludwig D, Decker J, Kaspar-Schoenefeld S, Lilley KS, Mülleder M (2022). dia-PASEF data analysis using FragPipe and DIA-NN for deep proteomics of low sample amounts. Nat. Commun..

[14] Zhao D, Jiang L, Wang L, Wu Z, Li Z, Shi W, Li P, Jiang Y, Li H (2018). Integrated metabolomics and proteomics approach to identify metabolic abnormalities in rats with *Dioscorea bulbifera* rhizome-induced hepatotoxicity. Chem. Res. Toxicol..

[15] Wei X, He Y, Wan H, Yin J, Lin B, Ding Z, Yang J, Zhou H (2023). Integrated transcriptomics, proteomics and metabolomics to identify biomarkers of astragaloside IV against cerebral ischemic injury in rats. Food Funct..

[16] Hynek R, Michalus I, Cejnar P, Šantrůček J, Seidlová S, Kučková Š, Sázelová P, Kašička V (2021). In-bone protein digestion followed by LC-MS/MS peptide analysis as a new way towards the routine proteomic characterization of human maxillary and mandibular bone tissue in oral surgery. Electrophoresis.

[17] Xu S, Zhong A, Zhang Y, Zhao L, Guo Y, Bai X, Yin P, Hua S (2024). Bone marrow mesenchymal stem cells therapy regulates sphingolipid and glycerophospholipid metabolism to promote neurological recovery in stroke rats: A metabolomics analysis. Exp. Neurol..

[18] Li J, Sun Z, Luo G, Wang S, Cui H, Yao Z, Xiong H, He Y, Qian Y, Fan C (2021). Quercetin attenuates trauma-induced heterotopic ossification by tuning immune cell infiltration and related inflammatory insult. Front. Immunol..

[19] Kanehisa M, Furumichi M, Sato Y, Kawashima M, Ishiguro-Watanabe M (2023). KEGG for taxonomy-based analysis of pathways and genomes. Nucleic Acids Res..

[20] Marie P, Haÿ E, Saidak Z (2014). Integrin and cadherin signaling in bone: Role and potential therapeutic targets. Trends Endocrinol. Metab..

[21] Weyts F, Li Y, van Leeuwen J, Weinans H, Chien S (2002). ERK activation and alpha v beta 3 integrin signaling through Shc recruitment in response to mechanical stimulation in human osteoblasts. J. Cell. Biochem..

[22] Yavropoulou M, Yovos J (2016). The molecular basis of bone mechanotransduction. J. Musculoskelet. Neuronal Interact..

[23] Kim H, Wrann C, Jedrychowski M, Vidoni S, Kitase Y, Nagano K, Zhou C, Chou J, Parkman V, Novick S, Strutzenberg T, Pascal B, Le P, Brooks D, Roche A, Gerber K, Mattheis L, Chen W, Tu H, Bouxsein M, Griffin P, Baron R, Rosen C, Bonewald L, Spiegelman B (2019). Irisin mediates effects on bone and fat via αV integrin receptors. Cell.

[24] Clark E, Brugge J (1995). Integrins and signal transduction pathways: The road taken. Science.

[25] Salter D, Robb J, Wright M (1997). Electrophysiological responses of human bone cells to mechanical stimulation: Evidence for specific integrin function in mechanotransduction. J. Bone Miner. Res..

[26] Uda Y, Azab E, Sun N, Shi C, Pajevic P (2017). Osteocyte mechanobiology. Curr. Osteoporos. Rep..

[27] Long R, Nishida S, Kubota T, Wang Y, Sakata T, Elalieh H, Halloran B, Bikle D (2011). Skeletal unloading-induced insulin-like growth factor 1 (IGF-1) nonresponsiveness is not shared by platelet-derived growth factor: The selective role of integrins in IGF-1 signaling. J. Bone Miner. Res..

[28] Kogawa M, Wijenayaka A, Ormsby R, Thomas G, Anderson P, Bonewald L, Findlay D, Atkins G (2013). Sclerostin regulates release of bone mineral by osteocytes by induction of carbonic anhydrase 2. J. Bone Miner. Res..

[29] Huitema L, Vaandrager A (2007). What triggers cell-mediated mineralization?. Front. Biosci..

[30] Alvarez L, Fanjul M, Carter N, Hollande E (2001). Carbonic anhydrase II associated with plasma membrane in a human pancreatic duct cell line (CAPAN-1). J. Histochem. Cytochem..

[31] Mahieu I, Becq F, Wolfensberger T, Gola M, Carter N, Hollande E (1994). The expression of carbonic anhydrases II and IV in the human pancreatic cancer cell line (Capan 1) is associated with bicarbonate ion channels. Biol. Cell.

[32] Chang X, Zheng Y, Yang Q, Wang L, Pan J, Xia Y, Yan X, Han J (2012). Carbonic anhydrase I (CA1) is involved in the process of bone formation and is susceptible to ankylosing spondylitis. Arthritis Res. Ther..

[33] Shi C, Uda Y, Dedic C, Azab E, Sun N, Hussein A, Petty C, Fulzele K, Mitterberger-Vogt M, Zwerschke W, Pereira R, Wang K, Pajevic P (2018). Carbonic anhydrase III protects osteocytes from oxidative stress. FASEB J..

[34] Roy P, Reavey E, Rayne M, Roy S, Abed El Baky M, Ishii Y, Bartholomew C (2010). Enhanced sensitivity to hydrogen peroxide-induced apoptosis in Evi1 transformed Rat1 fibroblasts due to repression of carbonic anhydrase III. FEBS J..

[35] Saftig P, Hunziker E, Wehmeyer O, Jones S, Boyde A, Rommerskirch W, Moritz J, Schu P, von Figura K (1998). Impaired osteoclastic bone resorption leads to osteopetrosis in cathepsin-K-deficient mice. Proc. Natl. Acad. Sci. USA.

[36] Moghaddam A, Müller U, Roth H, Wentzensen A, Grützner P, Zimmermann G (2011). TRACP 5b and CTX as osteological markers of delayed fracture healing. Injury.

[37] Chen K, Liu Y, He J, Pavlos N, Wang C, Kenny J, Yuan J, Zhang Q, Xu J, He W (2020). Steroid-induced osteonecrosis of the femoral head reveals enhanced reactive oxygen species and hyperactive osteoclasts. Int. J. Biol. Sci..

[38] Zhang P, Xu H, Wang P, Dong R, Xia C, Shi Z, Xu R, Fang L, Zou Z, Ge Q, Tong P, Jin H (2019). Yougui pills exert osteoprotective effects on rabbit steroid-related osteonecrosis of the femoral head by activating β-catenin. Biomed. Pharmacother..

[39] Liying L, Jidong S, Jun L, Na H, Juan Y, Shugang H, Rui M, Wei W (2020). Isoform 1 of fibrinogen alpha chain precursor is a potential biomarker for steroid-induced osteonecrosis of the femoral head. Proteomics Clin. Appl..

[40] Fan Y, Pengbo L, Hao D, Changqing Z, Zhenhong Z (2018). Collagen type V a2 (COL5A2) is decreased in steroid-induced necrosis of the femoral head. Am. J. Transl. Res..

[41] Ning Y, Hongzhi W, Weicheng Z, Houyi S, Meng L, Yaozeng X, Lixin H, Dechun G (2021). Integrated analysis of transcriptome and proteome to explore the genes related to steroid-induced femoral head necrosis. Exp. Cell Res..

[42] Wang J, Dong X, Ma F, Li C, Bu R, Lu J, Gao J, Xue P (2020). Metabolomics profiling reveals Echinops latifolius Tausch improves the trabecular micro-architecture of ovariectomized rats mainly via intervening amino acids and glycerophospholipids metabolism. J. Ethnopharmacol..

[43] Wang J, Yan D, Zhao A, Hou X, Zheng X, Chen P, Bao Y, Jia W, Hu C, Zhang Z, Jia W (2019). Discovery of potential biomarkers for osteoporosis using LC-MS/MS metabolomic methods. Osteoporos. Int..

[44] Zaitseva O, Shandrenko S, Veliky M (2015). Biochemical markers of bone collagen type I metabolism. Ukrainian Biochem. J..

[45] Swank K, Furness J, Baker E, Gehrke C, Biebelhausen S, Baker K (2020). Metabolomic profiling in the characterization of degenerative bone and joint diseases. Metabolites.

[46] Ren X, Shao Z, Fan W, Wang Z, Chen K, Yu X (2020). Untargeted metabolomics reveals the effect of lovastatin on steroid-induced necrosis of the femoral head in rabbits. J. Orthop. Surg. Res..

[47] Yin H, Yuan Z, Wang D (2016). Multiple drilling combined with simvastatin versus multiple drilling alone for the treatment of avascular osteonecrosis of the femoral head: 3-year follow-up study. BMC Musculoskelet. Disord..

[48] Nozaki Y, Kumagai K, Miyata N, Niwa M (2012). Pravastatin reduces steroid-induced osteonecrosis of the femoral head in SHRSP rats. Acta Orthop..

[49] Roberts S, Stewart A, Sadler P, Farquharson C (2004). Human PHOSPHO1 exhibits high specific phosphoethanolamine and phosphocholine phosphatase activities. Biochem. J..

[50] Yadav M, Simão A, Narisawa S, Huesa C, McKee M, Farquharson C, Millán J (2011). Loss of skeletal mineralization by the simultaneous ablation of PHOSPHO1 and alkaline phosphatase function: A unified model of the mechanisms of initiation of skeletal calcification. J. Bone Miner. Res..

[51] Liu Y, Ren Z, Shao H, Wang X, Ma Y, Song W, Wu X, Zhang X, Li P, He Y, Wei X, Duan W (2023). Titanium alloy cannulated screws and biodegrade ceramic nails for treatment of femoral neck fractures: A finite element analysis. Injury.

