# Supplementary figures and images for: Integrated proteomics and metabolomics analysis of sclerosis-related proteins and femoral head necrosis following internal fixation of femoral neck fractures
Source: Sci Rep. 2024 Jun 8;14:13207. doi: 10.1038/s41598-024-63837-8 (PMC11162501; doi:10.1038/s41598-024-63837-8)

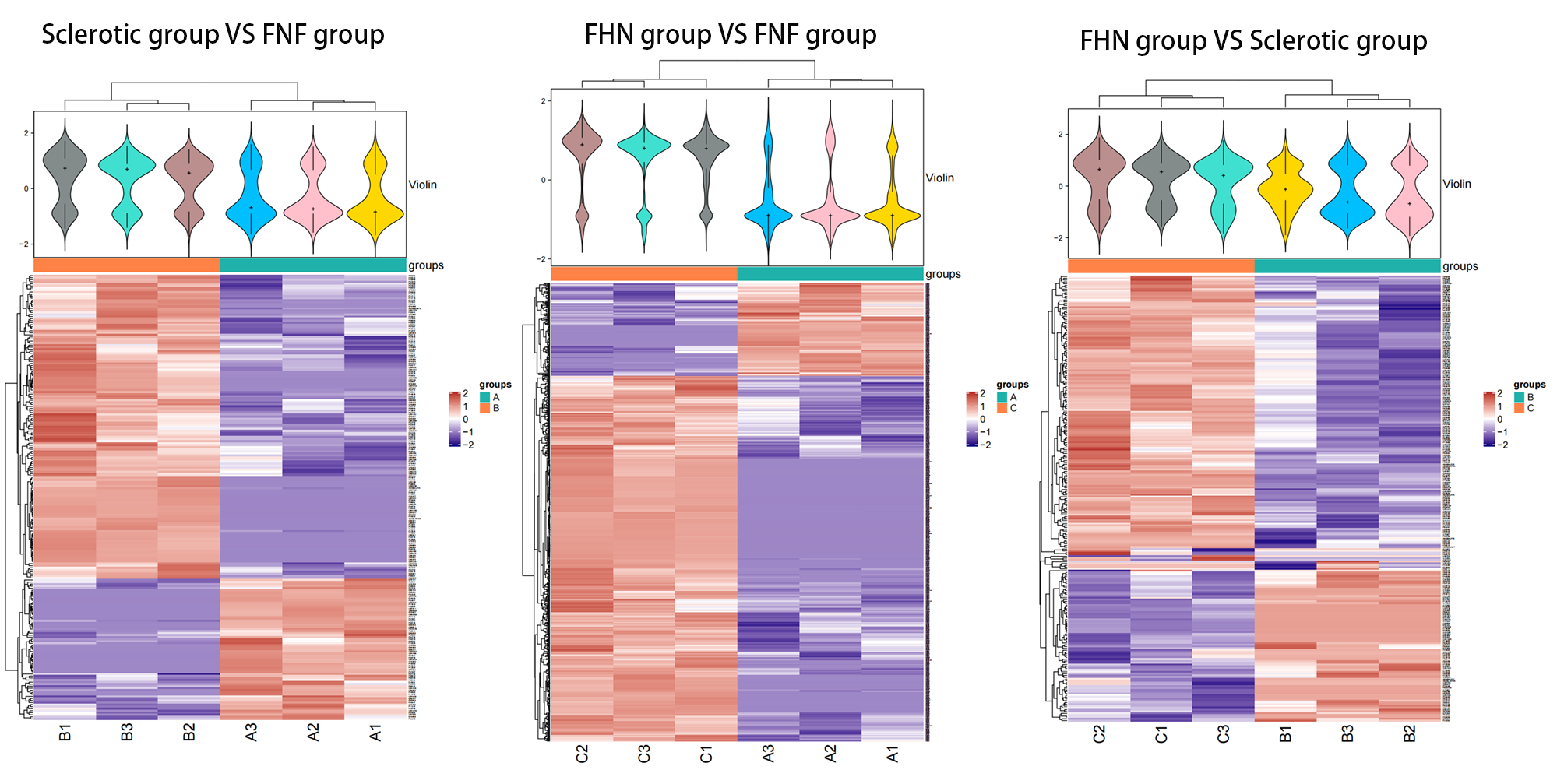

Supplement: Supplementary file 1 — Supplementary Figure S1. [file 41598_2024_63837_MOESM1_ESM.tif]

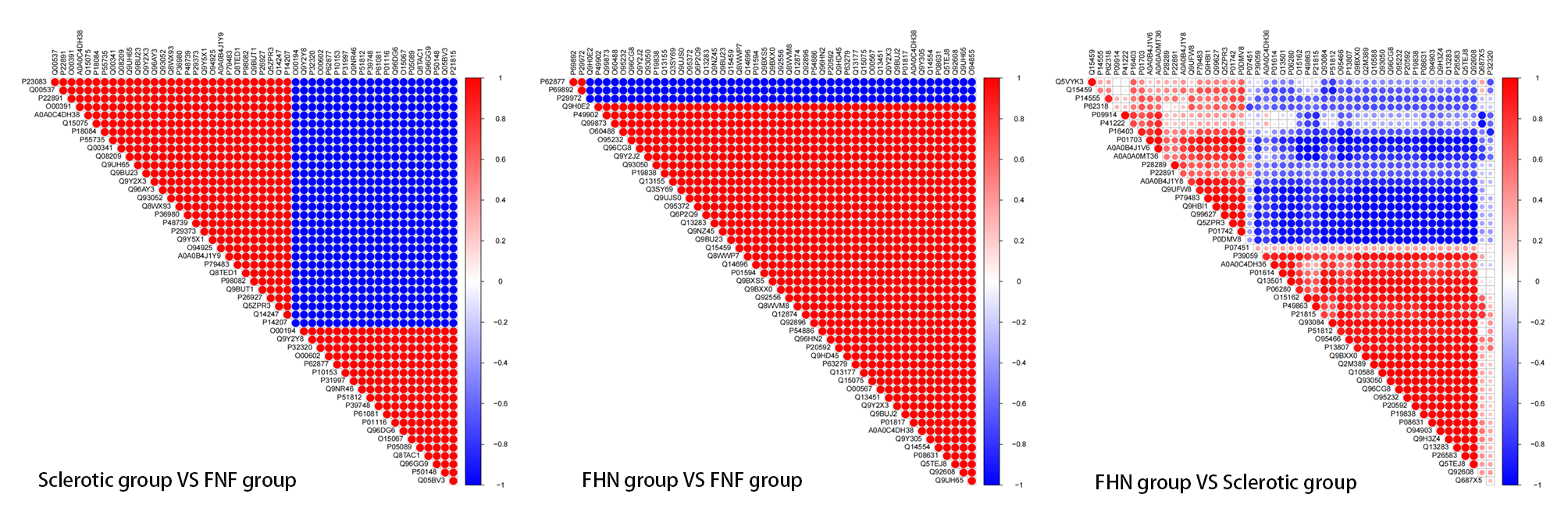

Supplement: Supplementary file 2 — Supplementary Figure S2. [file 41598_2024_63837_MOESM2_ESM.tif]

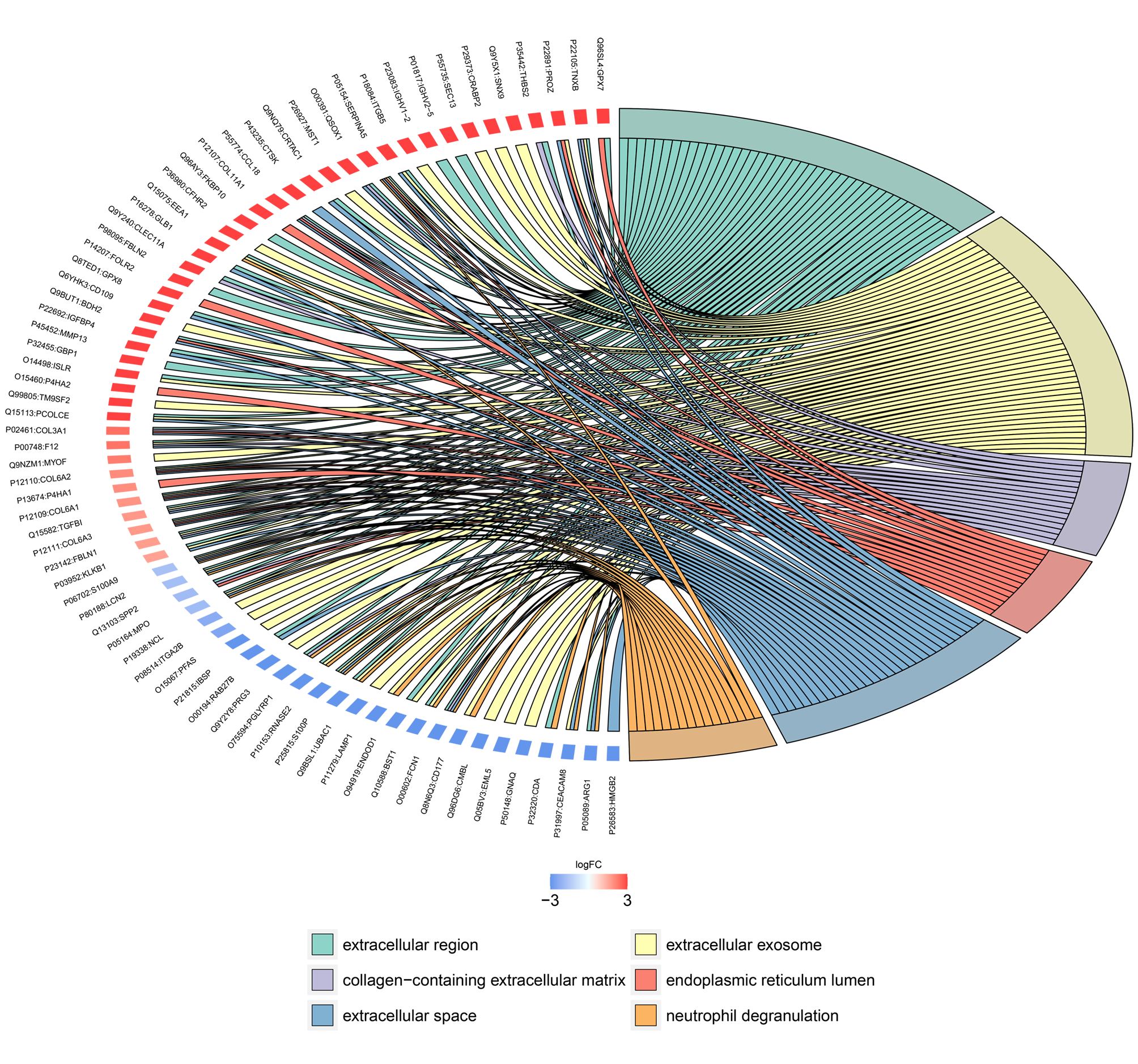

Supplement: Supplementary file 3 — Supplementary Figure S3. [file 41598_2024_63837_MOESM3_ESM.tif]

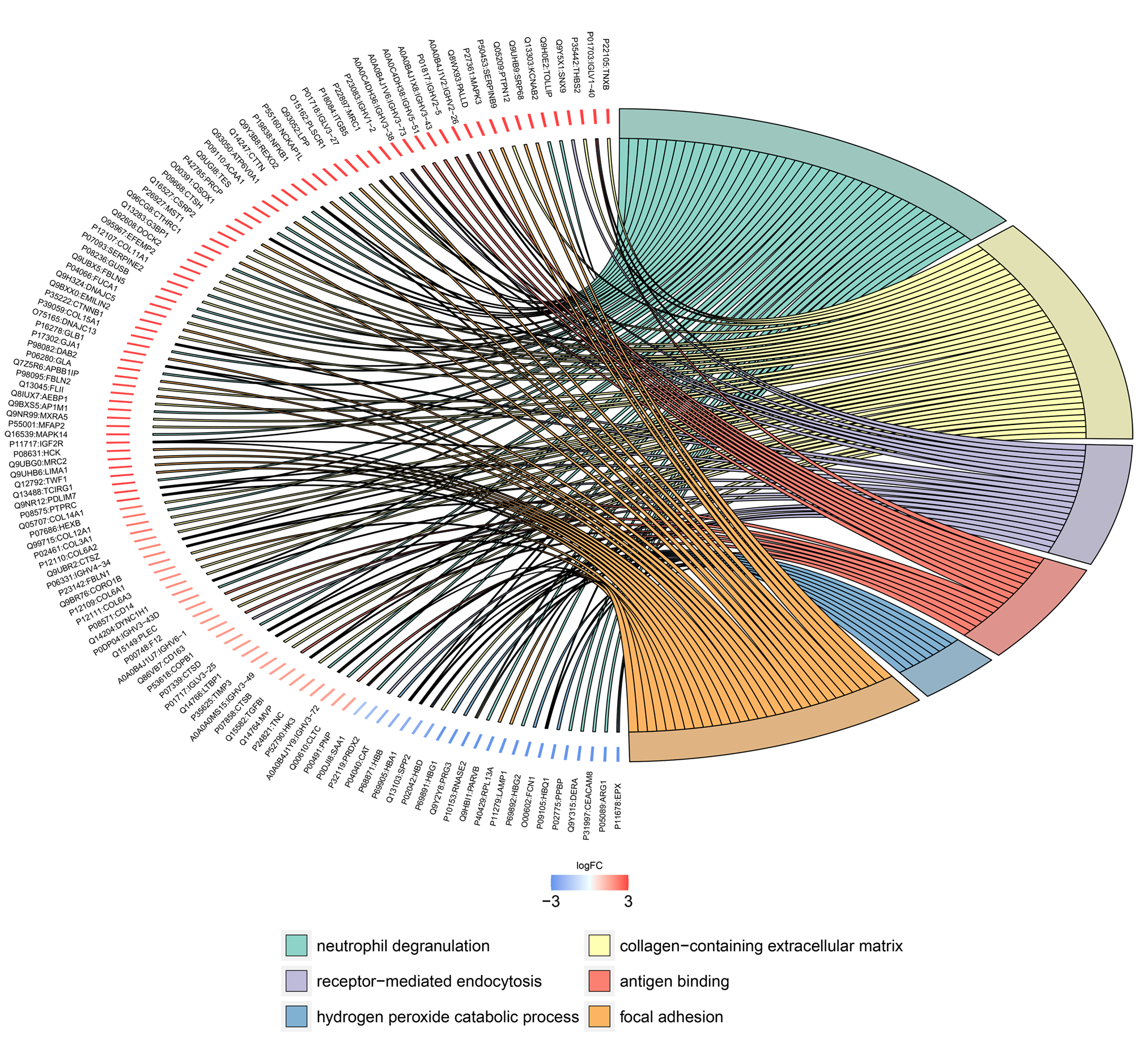

Supplement: Supplementary file 4 — Supplementary Figure S4. [file 41598_2024_63837_MOESM4_ESM.tif]

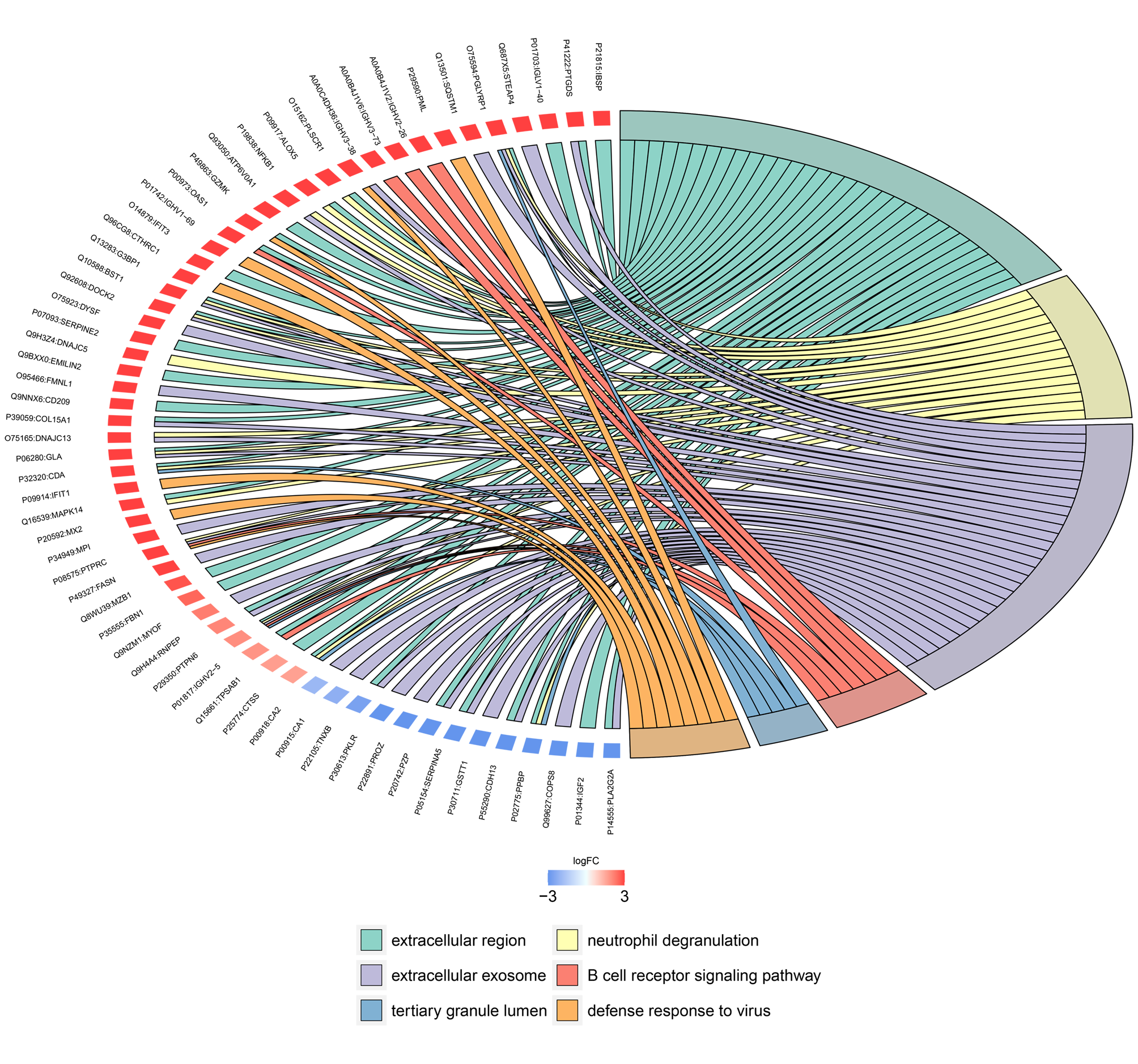

Supplement: Supplementary file 5 — Supplementary Figure S5. [file 41598_2024_63837_MOESM5_ESM.tif]

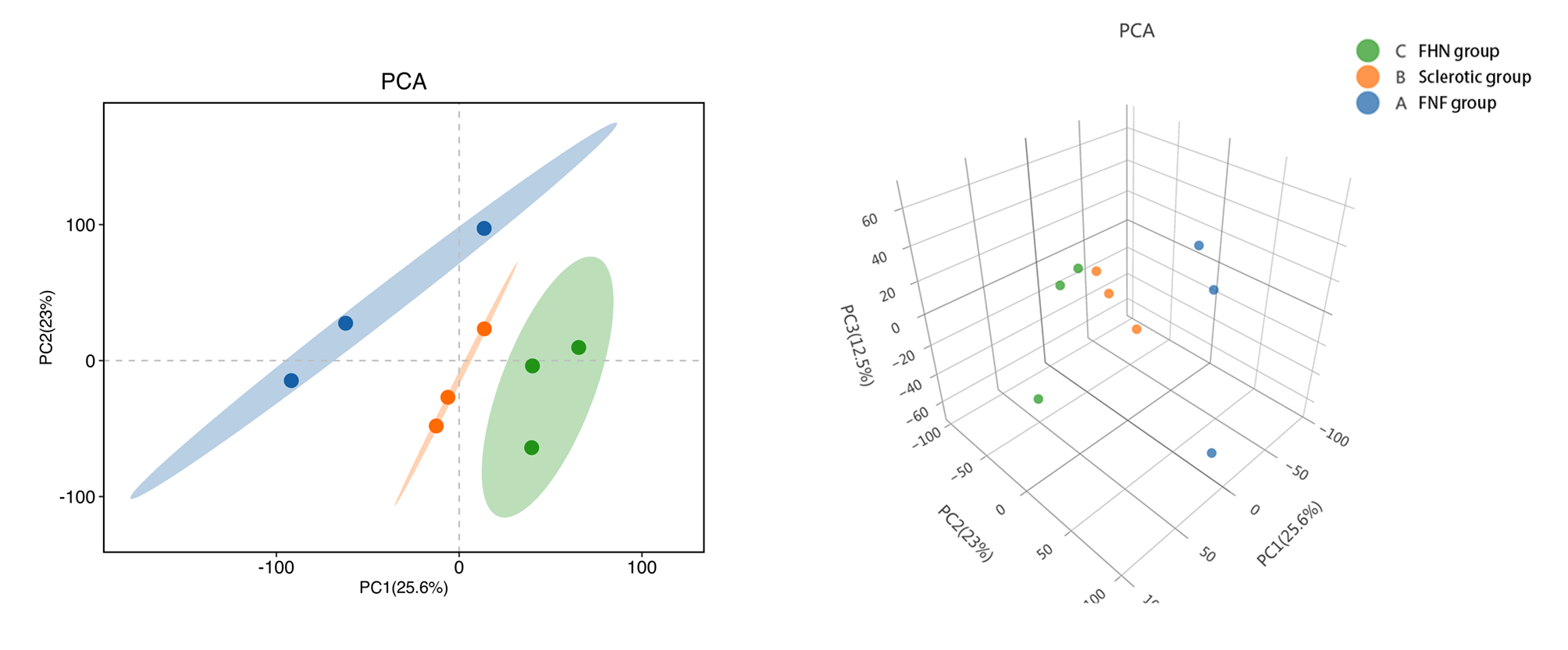

Supplement: Supplementary file 6 — Supplementary Figure S6. [file 41598_2024_63837_MOESM6_ESM.tif]

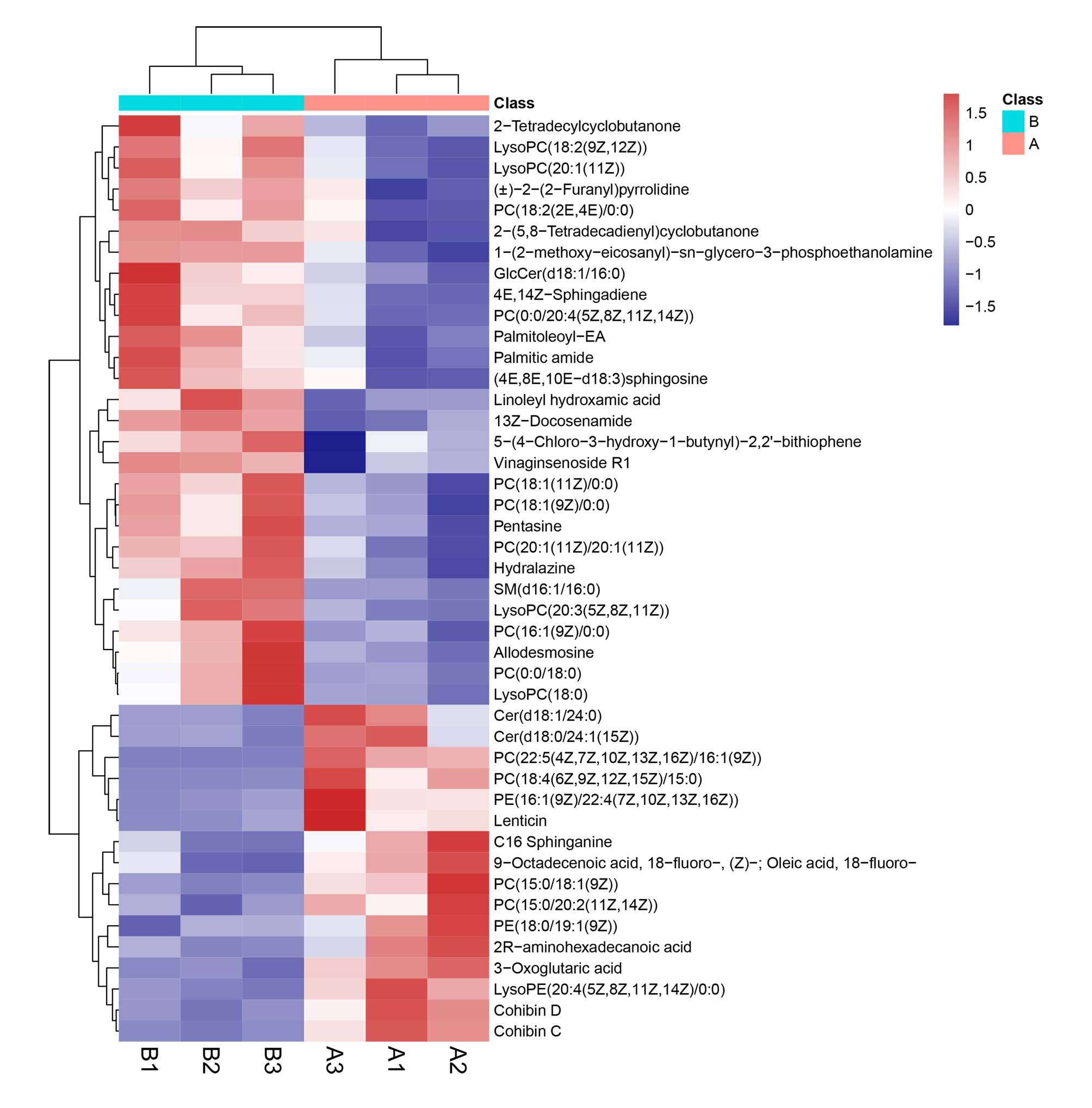

Supplement: Supplementary file 7 — Supplementary Figure S7. [file 41598_2024_63837_MOESM7_ESM.tif]

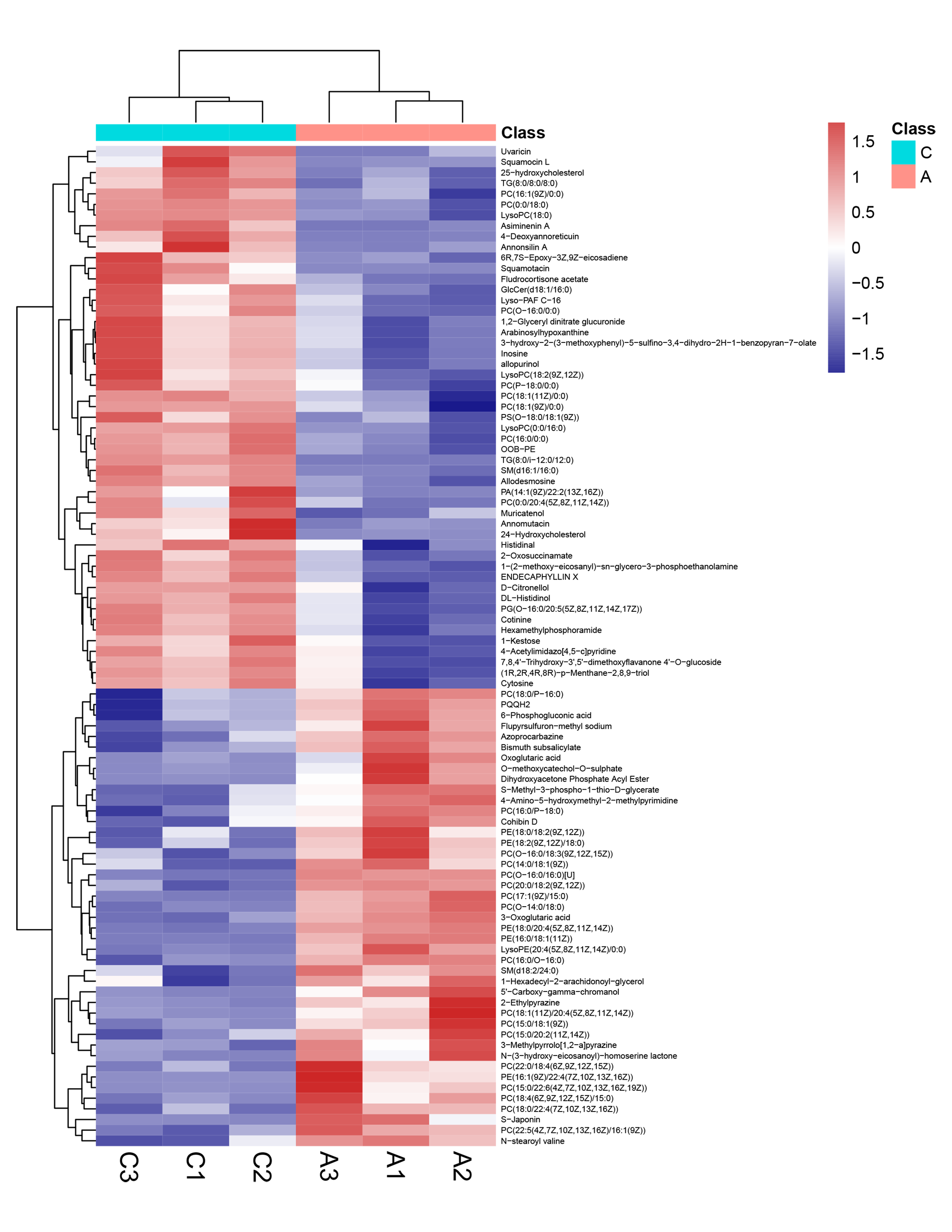

Supplement: Supplementary file 8 — Supplementary Figure S8. [file 41598_2024_63837_MOESM8_ESM.tif]

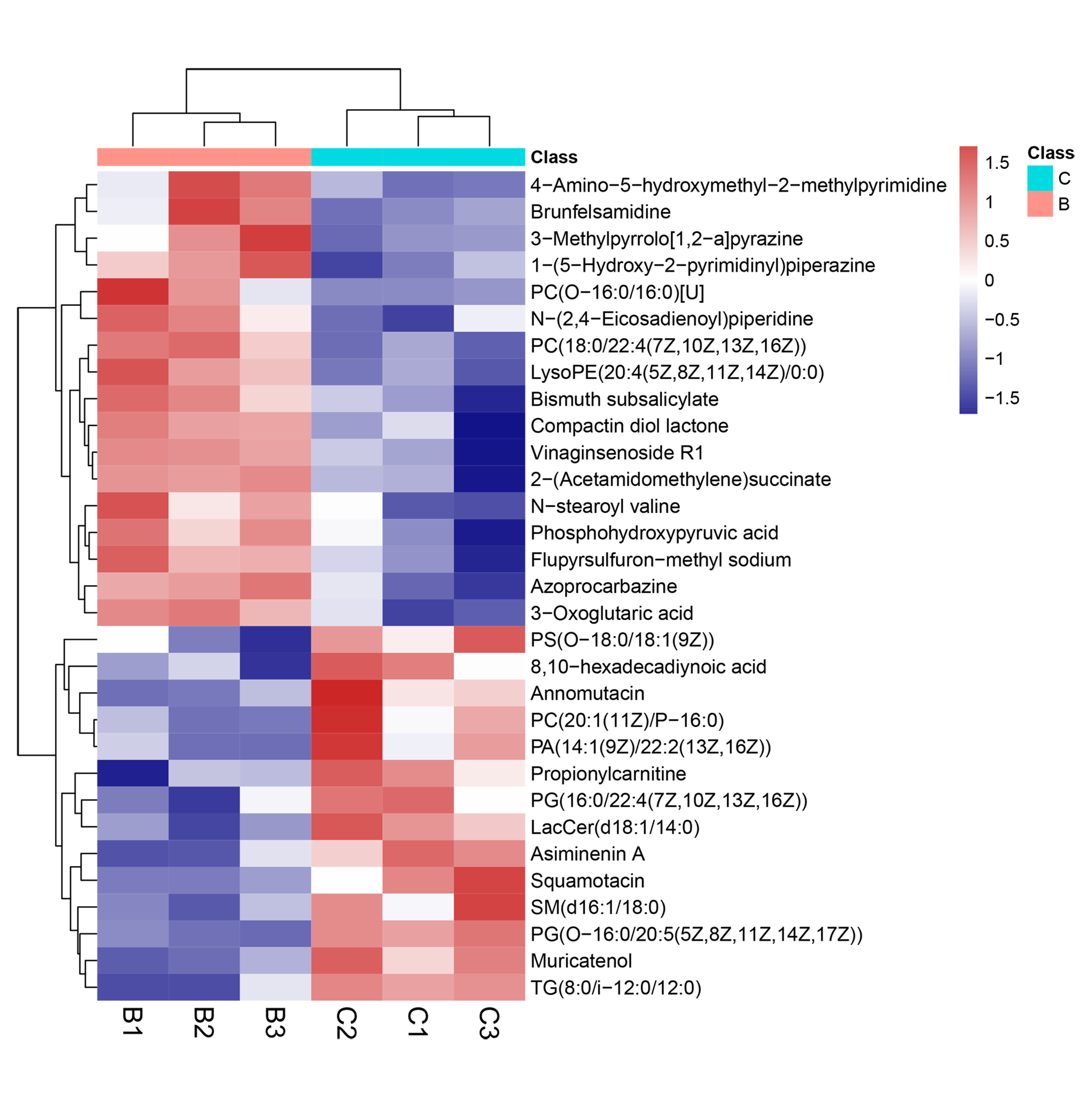

Supplement: Supplementary file 9 — Supplementary Figure S9. [file 41598_2024_63837_MOESM9_ESM.tif]

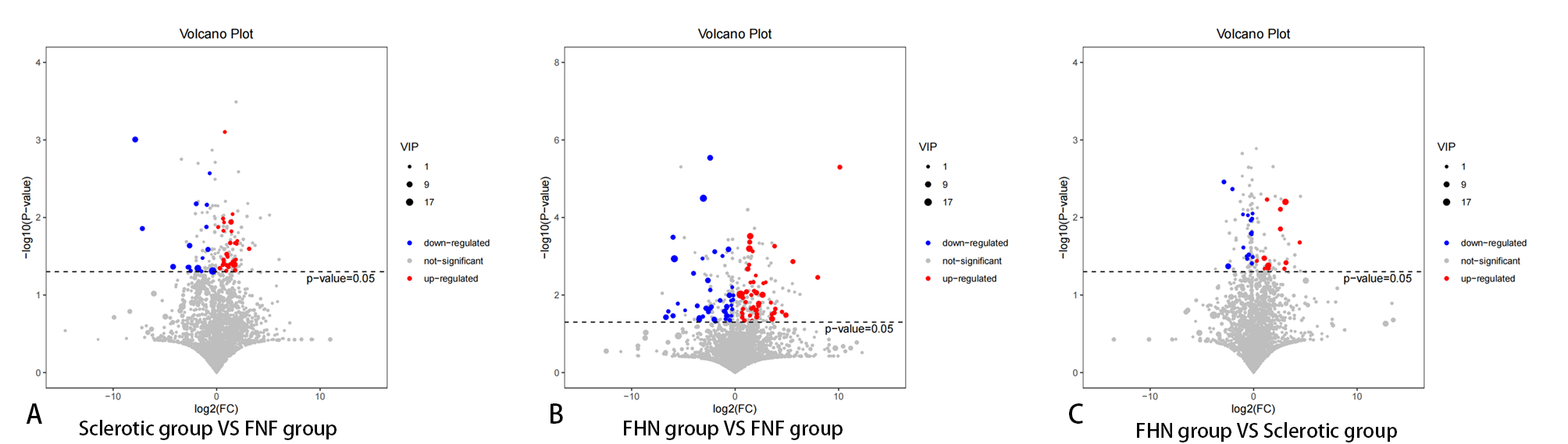

Supplement: Supplementary file 10 — Supplementary Figure S10. [file 41598_2024_63837_MOESM10_ESM.tif]

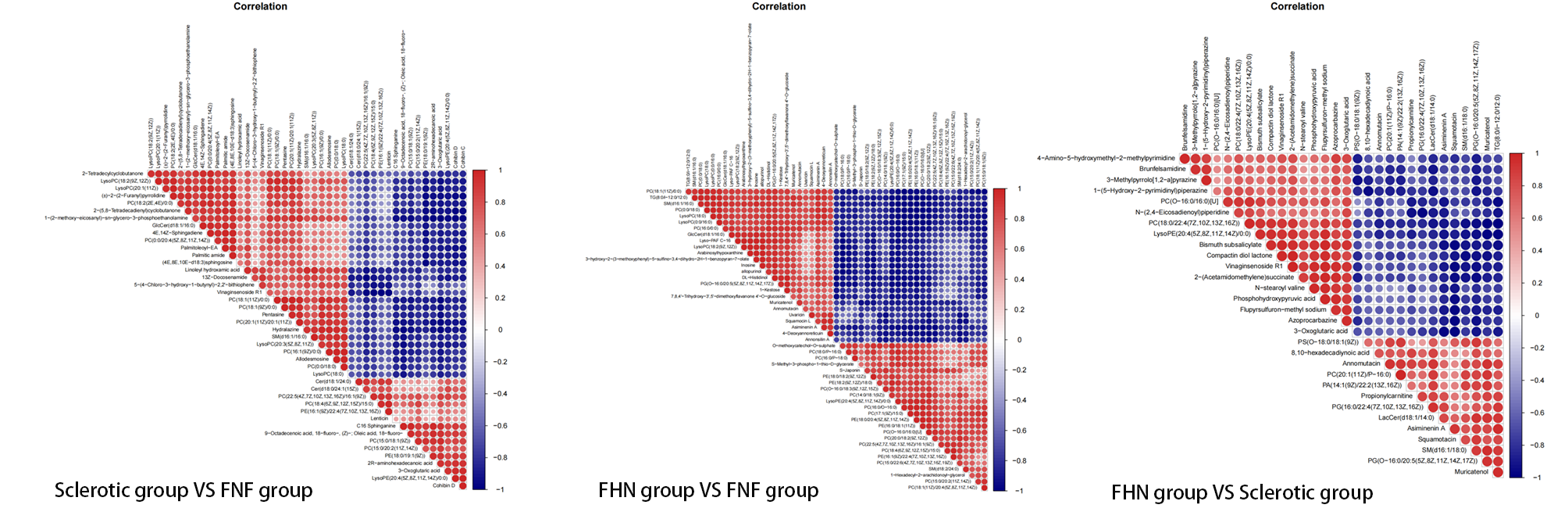

Supplement: Supplementary file 11 — Supplementary Figure S11. [file 41598_2024_63837_MOESM11_ESM.tif]

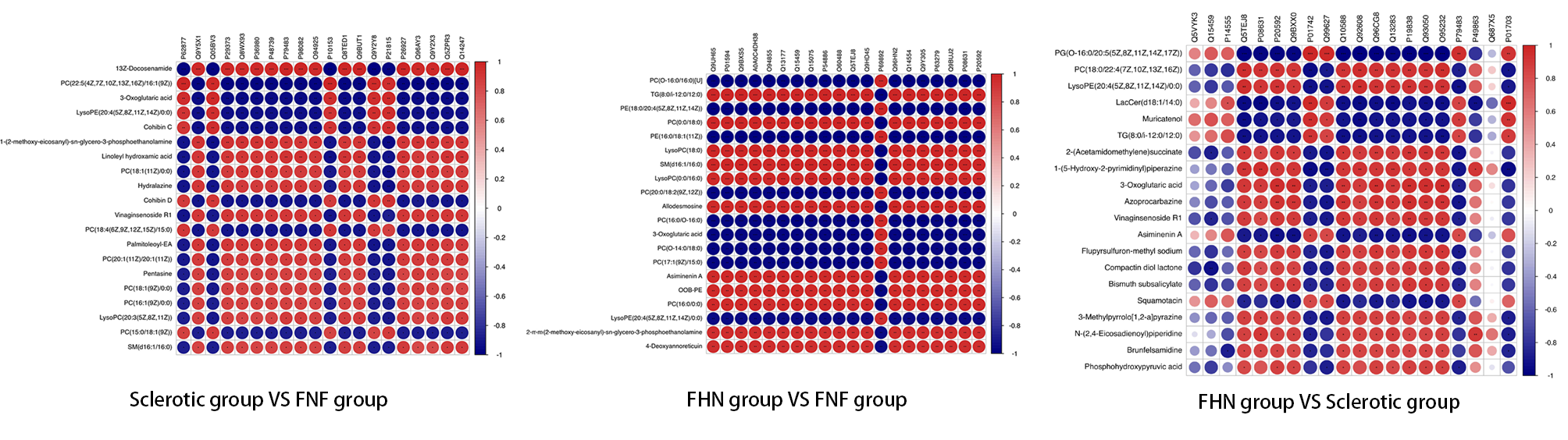

Supplement: Supplementary file 12 — Supplementary Figure S12. [file 41598_2024_63837_MOESM12_ESM.tif]

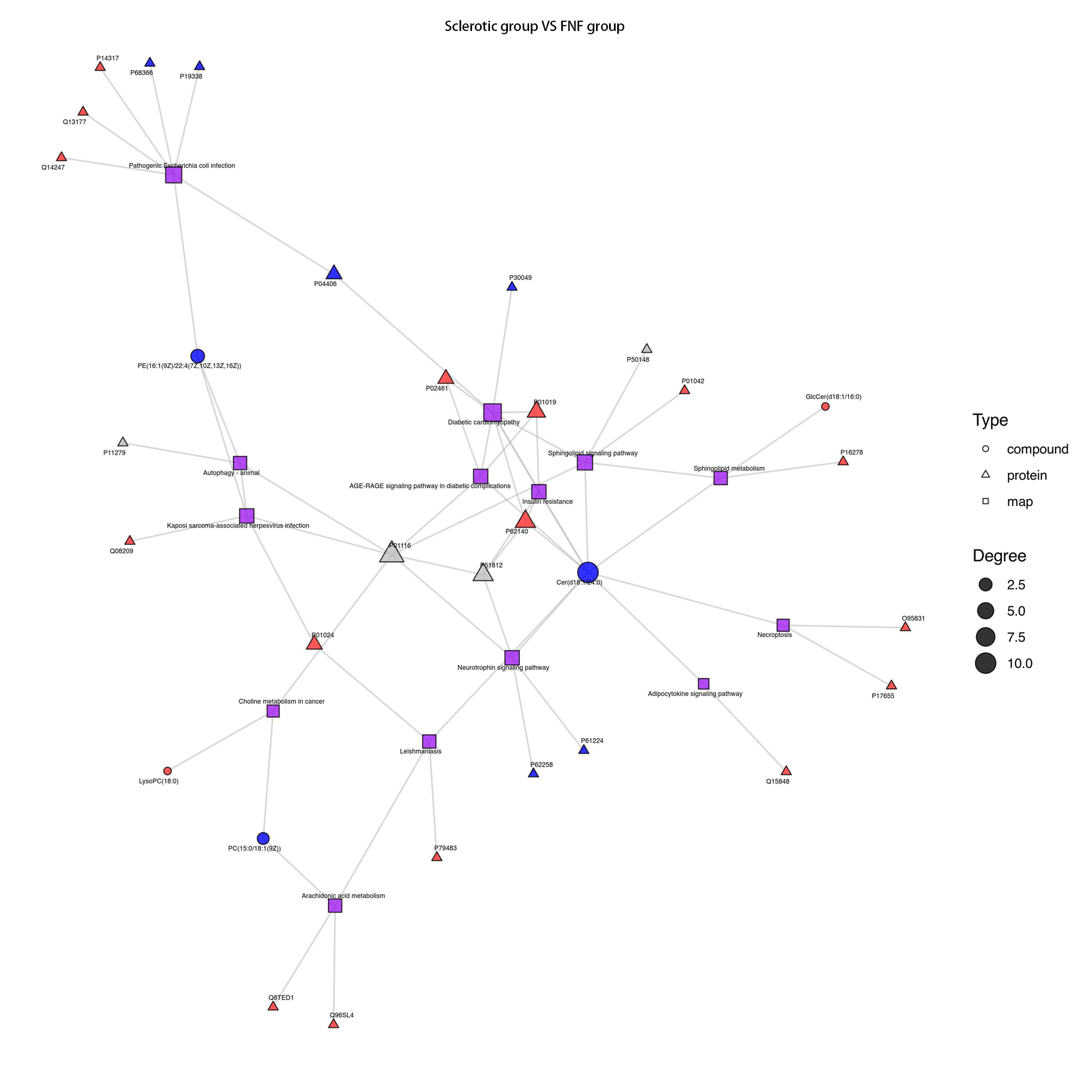

Supplement: Supplementary file 13 — Supplementary Figure S13. [file 41598_2024_63837_MOESM13_ESM.tif]

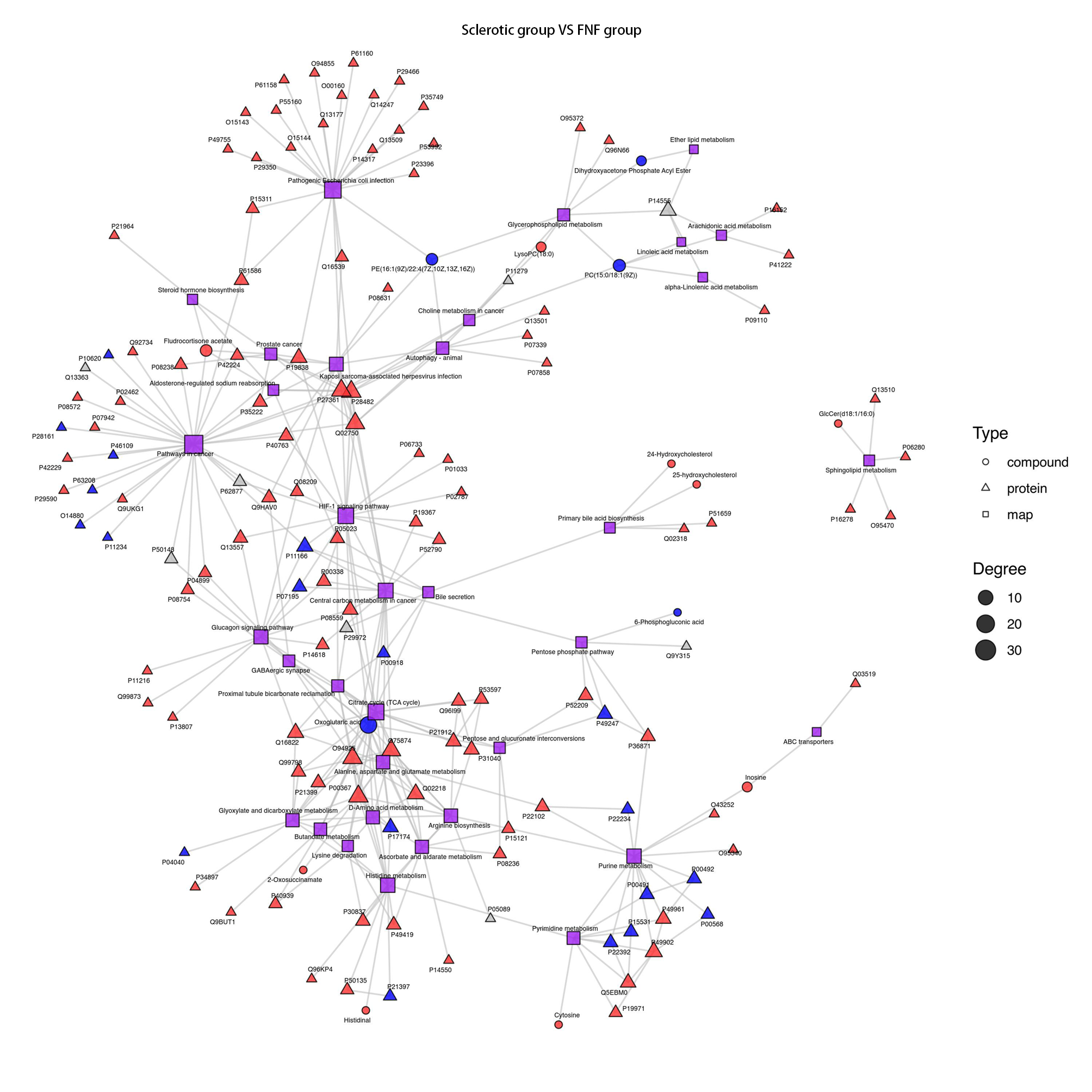

Supplement: Supplementary file 14 — Supplementary Figure S14. [file 41598_2024_63837_MOESM14_ESM.tif]

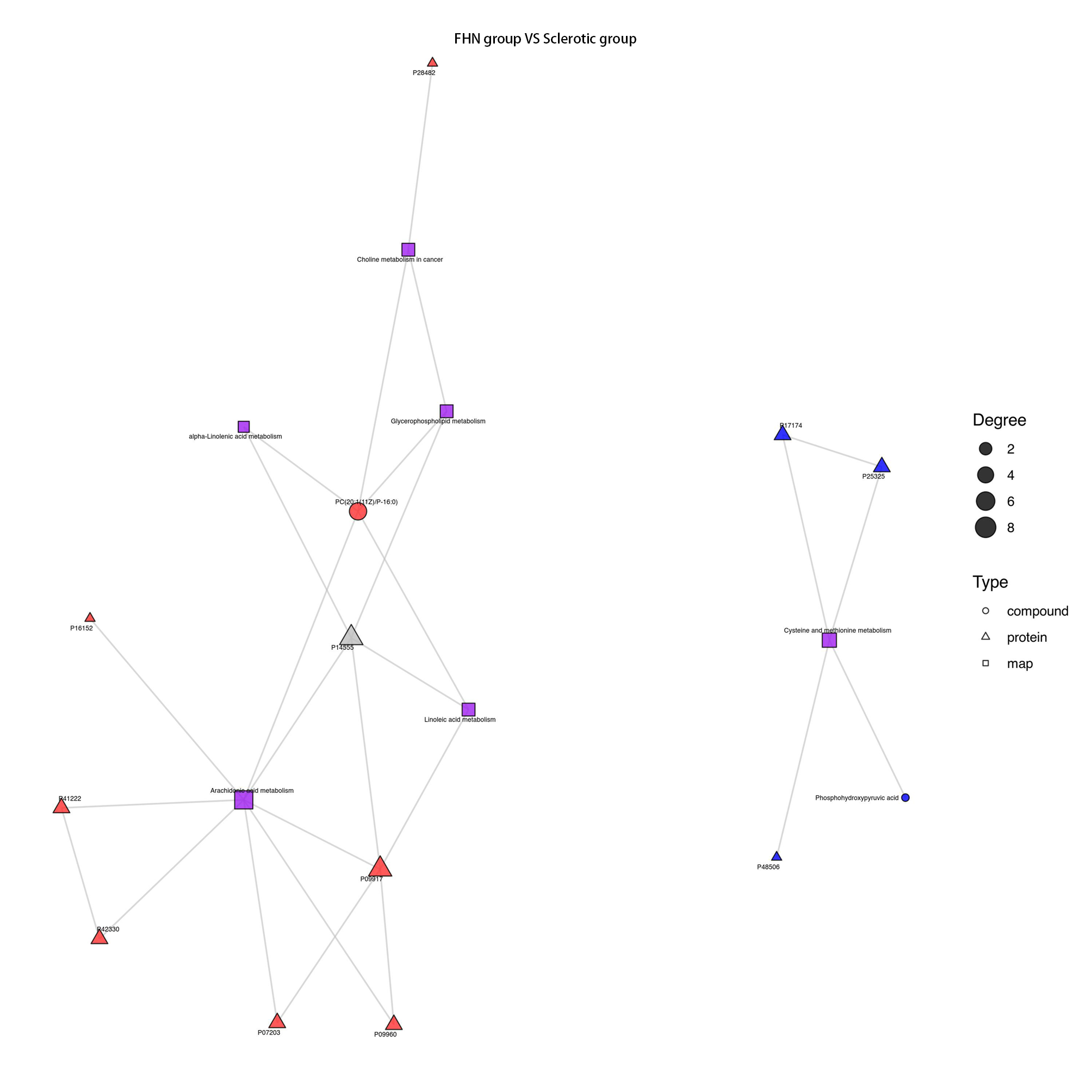

Supplement: Supplementary file 15 — Supplementary Figure S15. [file 41598_2024_63837_MOESM15_ESM.tif]

TNXB

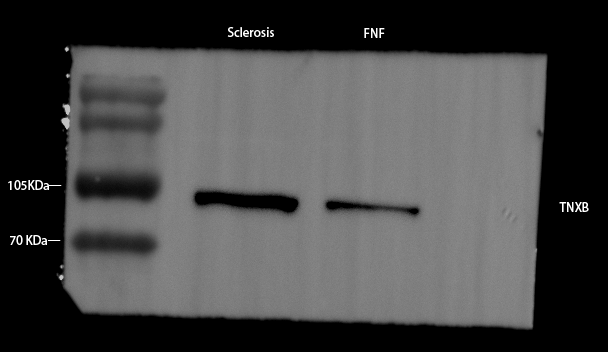

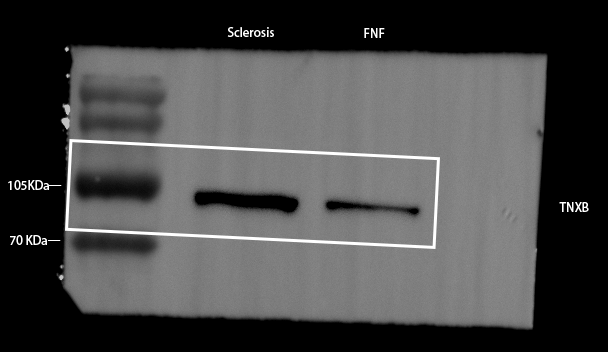


ITGB5


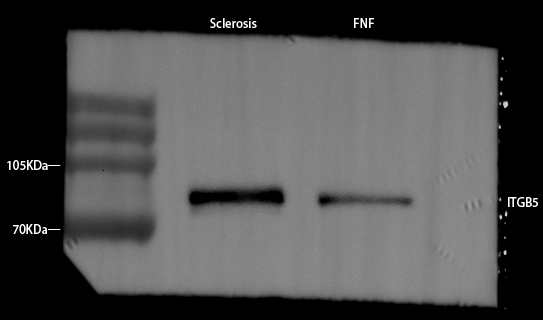

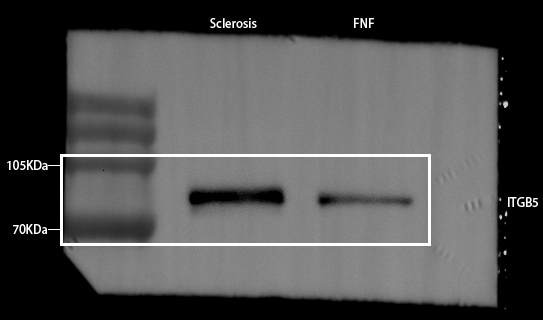


GAPDH


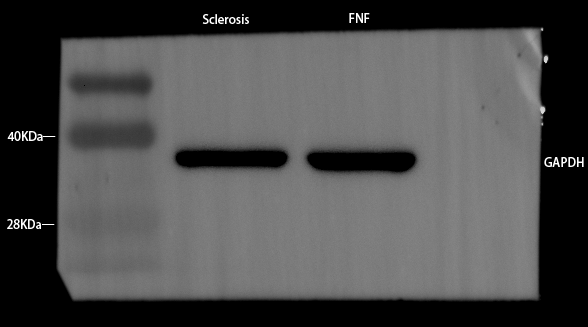

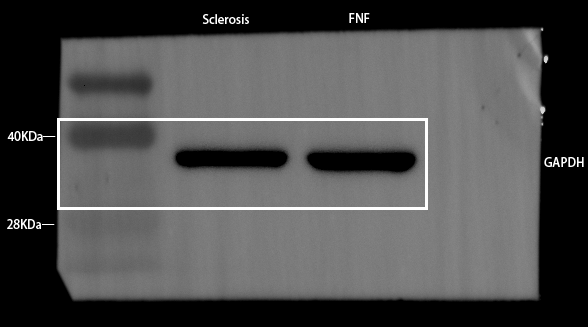


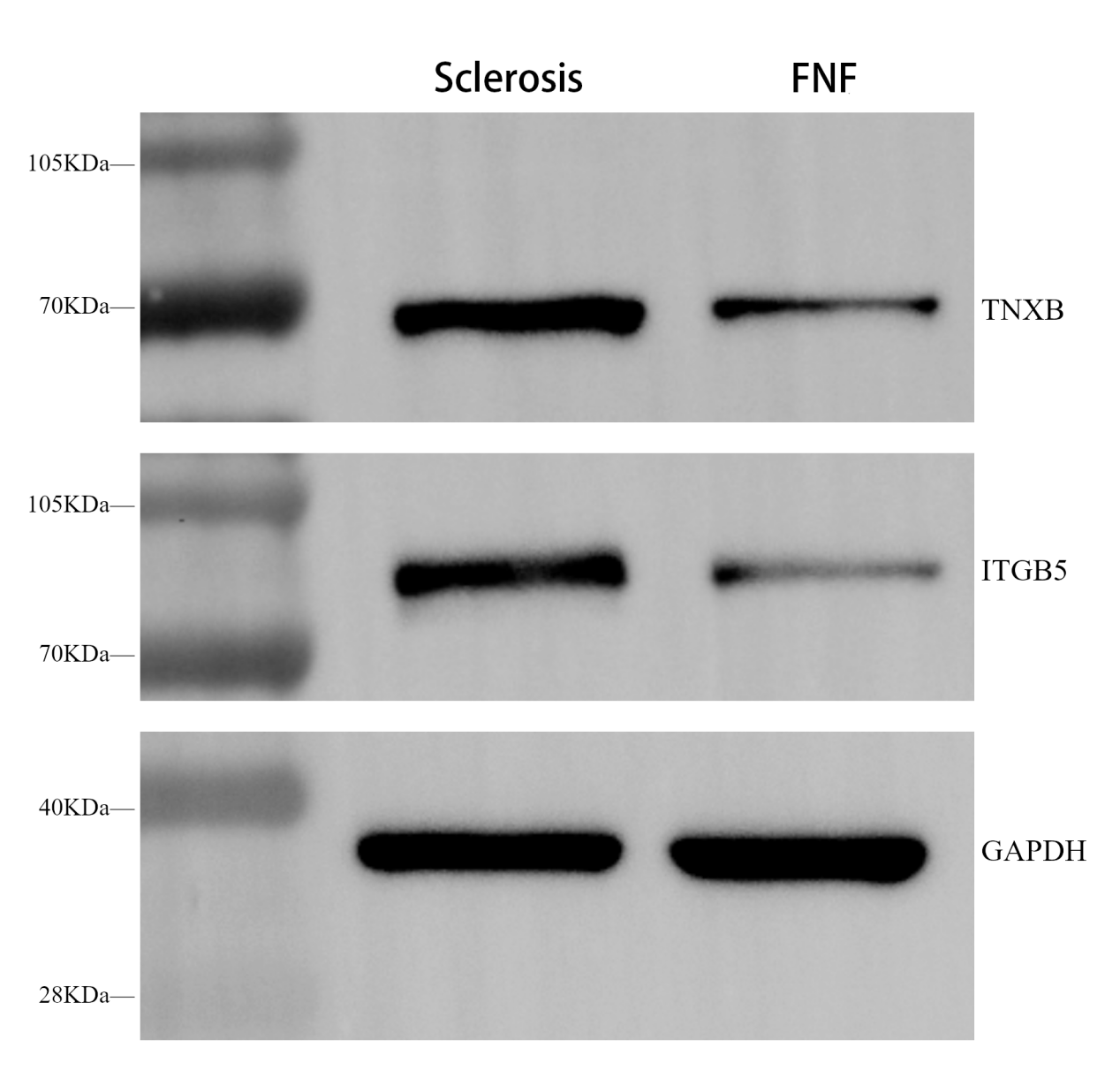


ACP5


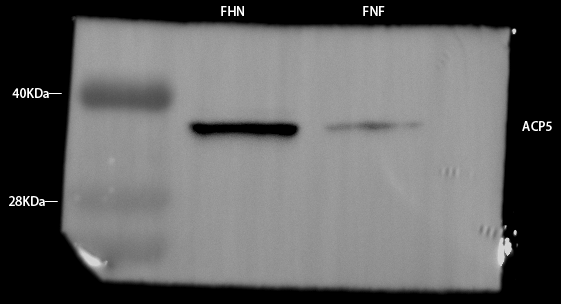

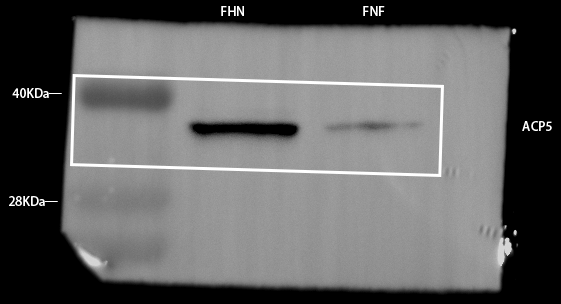


CTSK


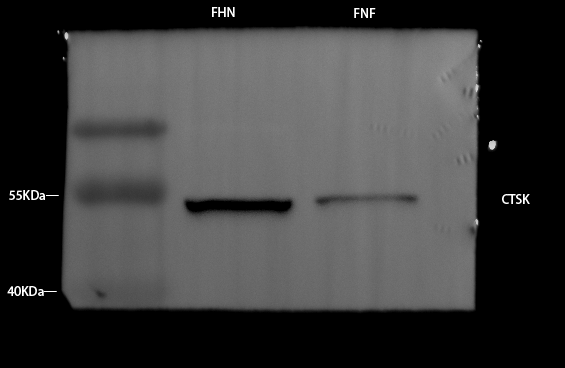

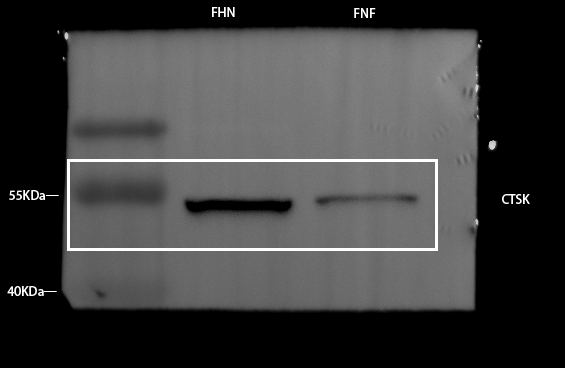


GAPDH


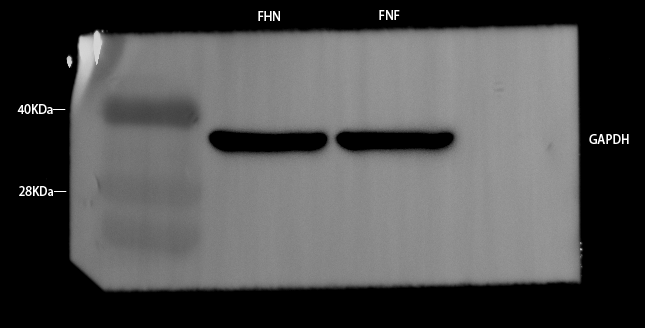

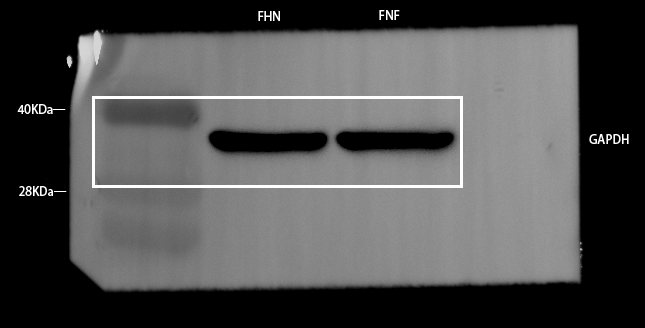


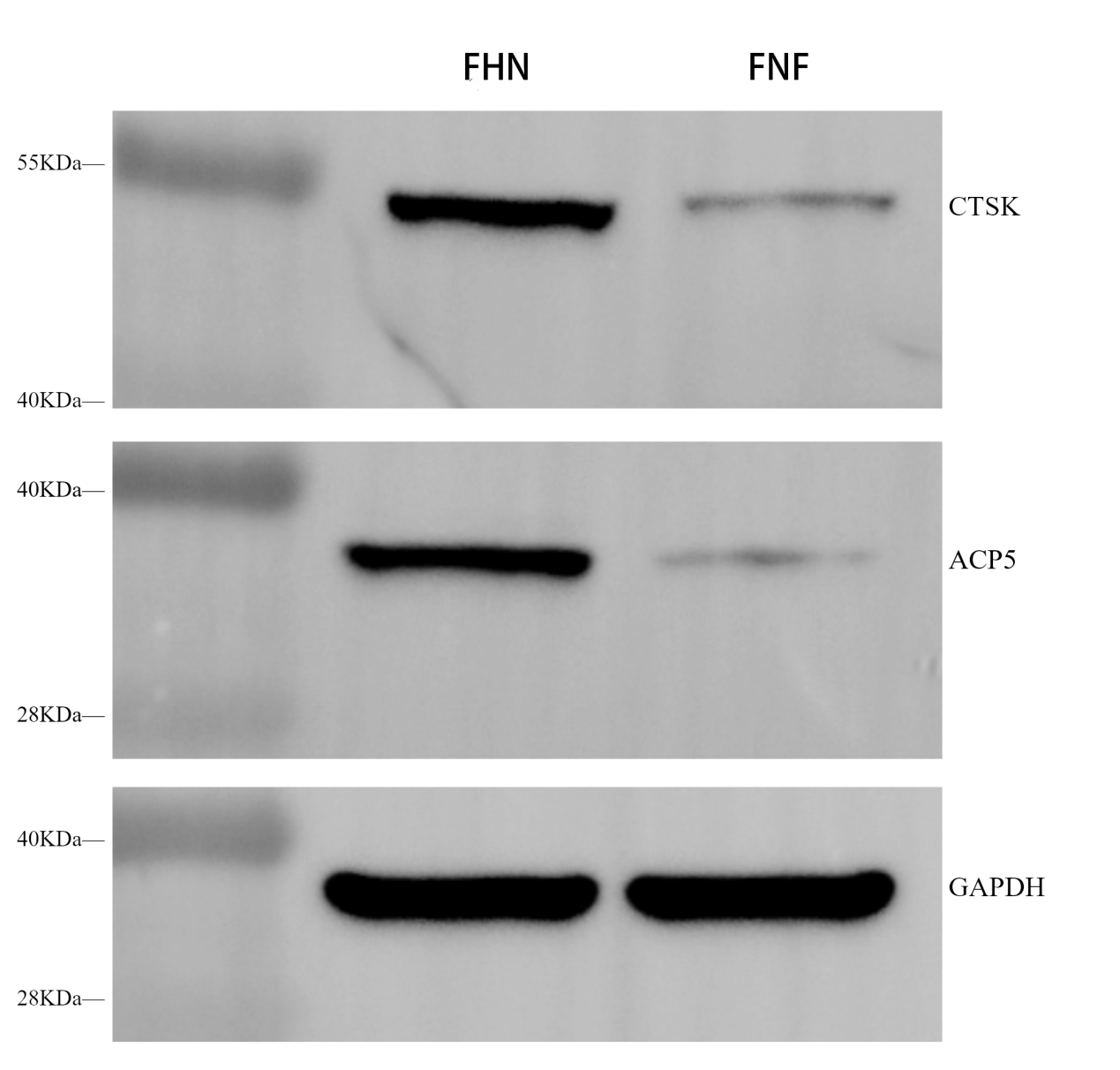


CA2


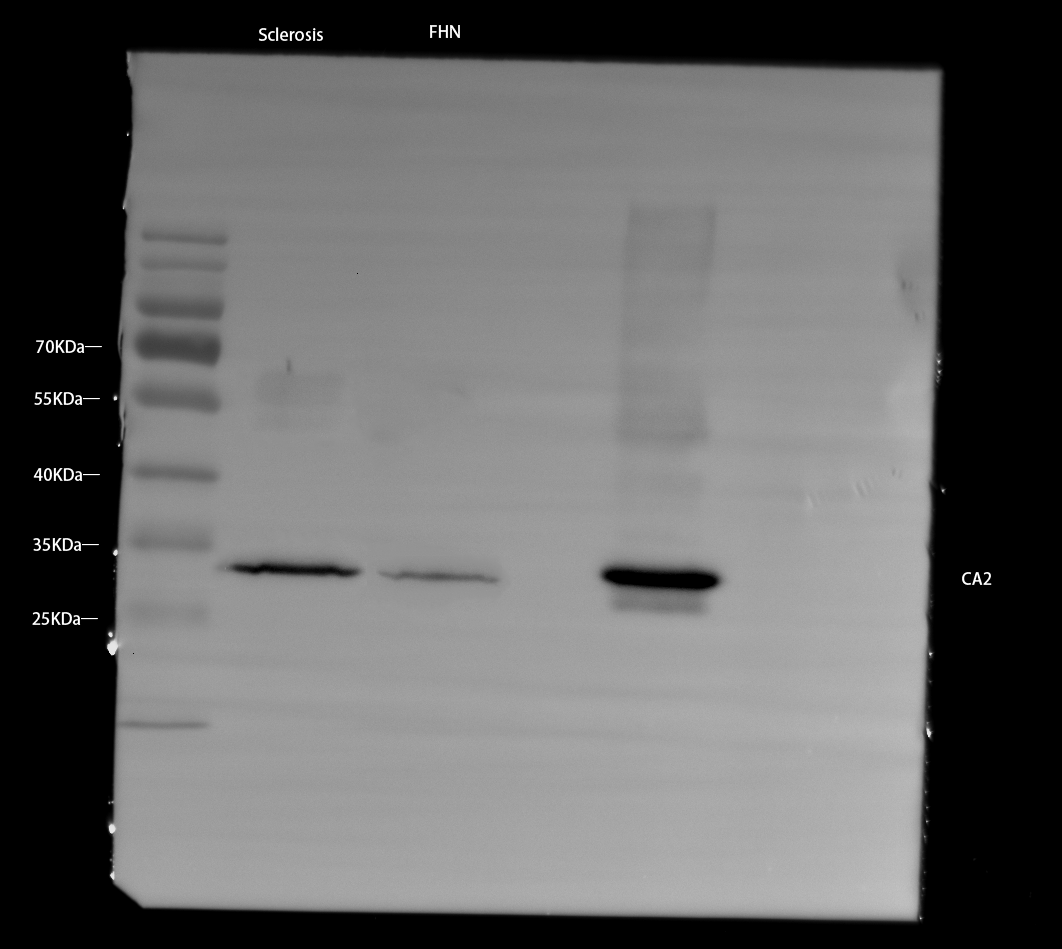

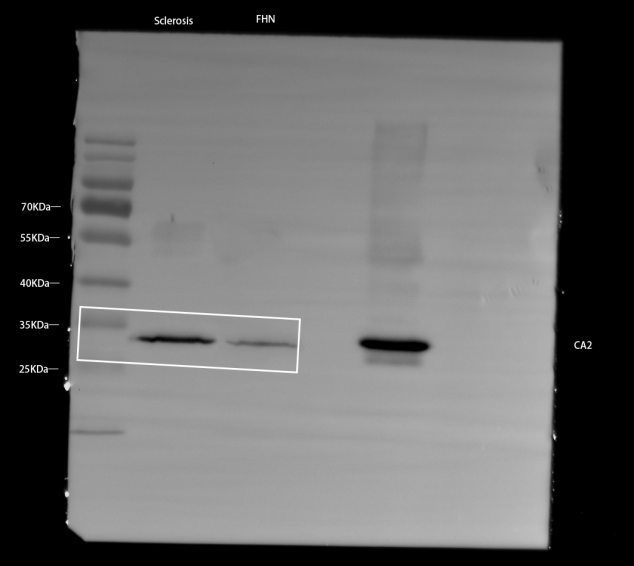


CA3


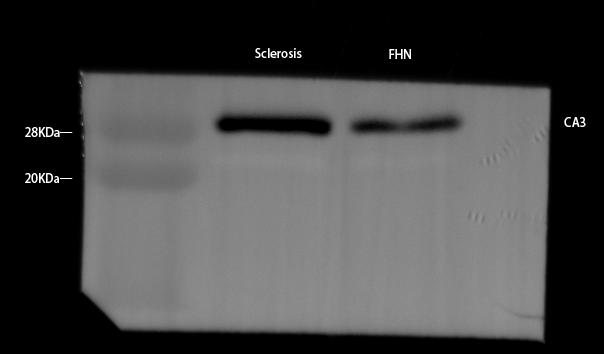

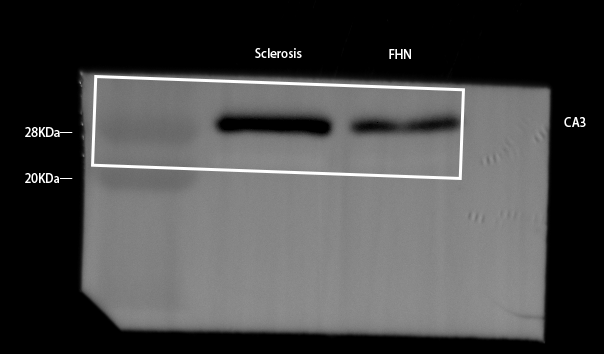


GAPDH


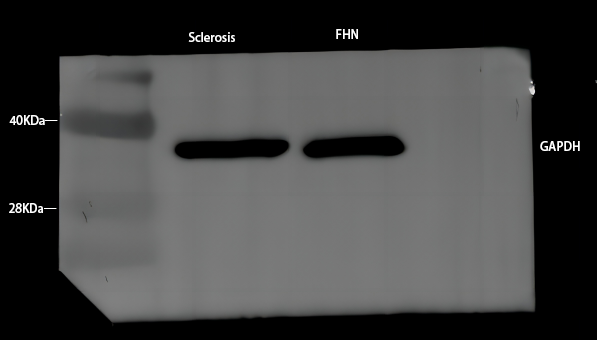

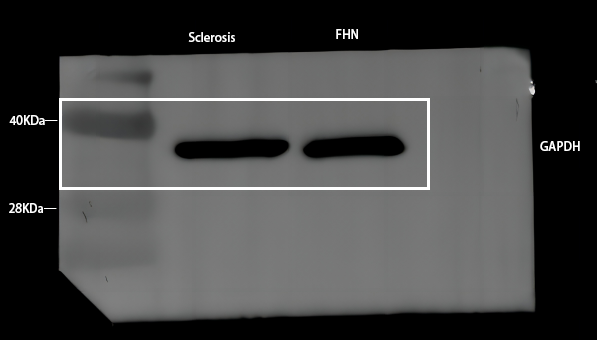


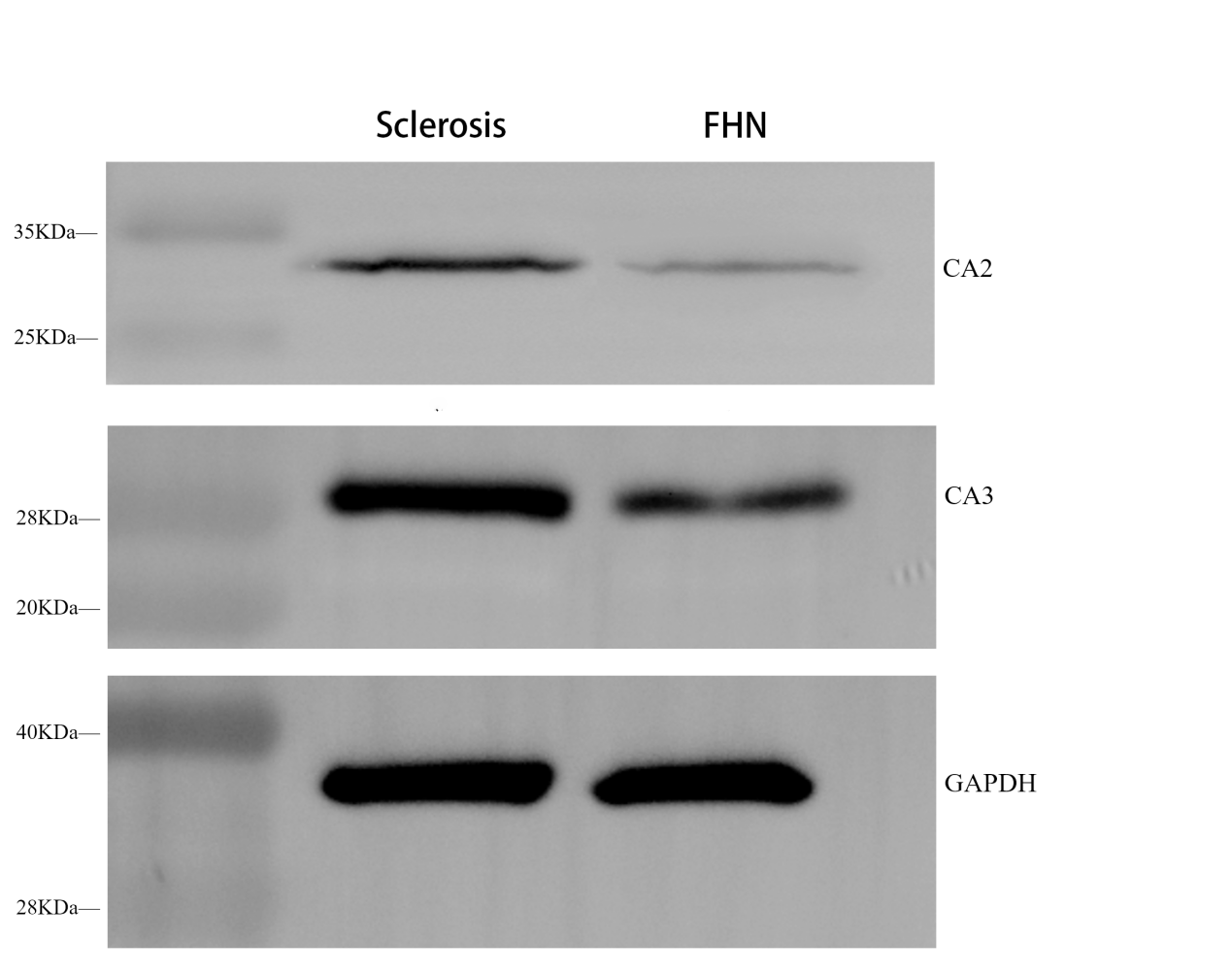

Supplement: Supplementary file 17 — Supplementary Information. [file 41598_2024_63837_MOESM17_ESM.docx]
