# Supplementary material for: Integrated proteomics and metabolomics analysis of sclerosis-related proteins and femoral head necrosis following internal fixation of femoral neck fractures
Source: Sci Rep. 2024 Jun 8;14:13207. doi: 10.1038/s41598-024-63837-8 (PMC11162501; doi:10.1038/s41598-024-63837-8)
Supplement: Supplementary file 16 — Supplementary Legends. [file 41598_2024_63837_MOESM16_ESM.docx]

Supplementary Figure 1. Clustered heat map showing the expression patterns of differentially expressed proteins in the three groups. The violin plots at the top represent the distribution of expression values, with wider violins indicating more concentrated data. The color fill represents different samples, and the "+" symbol in the middle of the violins represents the median expression value. The column annotation below the violins indicates the grouping of the samples. The clustered heat map below the violin plots shows the expression levels of each protein in different groups, with red indicating high expression and blue indicating low expression.

Supplementary Figure 2. Expression correlation analysis of differentially expressed proteins in the three groups. Red represents positive correlation, blue represents negative correlation, and the size of the circles represents the significance of the correlation test. The color depth represents the magnitude of the correlation coefficient.

Supplementary Figure 3. Chord diagram illustrating the relationship between the selected GO terms and the corresponding differentially expressed proteins. The left side shows protein-gene names, with red indicating upregulation and blue indicating downregulation. The right side shows the selected GO terms. Chord diagram differentially expressed proteins (DEPs) between the sclerosis group and the FNF group mainly associated with the biological process of the extracellular region, collagen-containing, and extracellular matrix regulation.

Supplementary Figure 4. Chord diagram illustrating the relationship between the selected GO terms and the corresponding differentially expressed proteins. The left side shows protein-gene names, with red indicating upregulation and blue indicating downregulation. The right side shows the selected GO terms. Chord diagram differentially expressed proteins (DEPs) between the FHN group and the FNF group mainly associated with the biological process of th eneutrophil degranulation, receptor-mediated endocytosis, hydrogen peroxide catabolic process, and collagen-containing extracellular matrix regulation.

Supplementary Figure 5. Chord diagram illustrating the relationship between the selected GO terms and corresponding differentially expressed proteins (DEPs). The left side shows protein-gene names, with red indicating upregulation and blue indicating downregulation. The right side shows the selected GO terms. Chord diagram DEPs between the FHN group and sclerosis group mainly associated with the biological process of neutrophil degranulation, receptor-mediated endocytosis, hydrogen peroxide catabolic process, and collagen-containing extracellular matrix regulation.

Supplementary Figure 6. Principal component analysis (PCA) among three groups. If the difference between two samples is significant, the coordinate points on the score plot will be relatively far apart and vice versa. The ellipse region represents a 95% confidence interval.

Supplementary Figure 7. Heat map of differentially expressed metabolites between the sclerosis group and FNF group. The horizontal axis represents sample names, and the vertical axis represents differentially expressed metabolites. The color gradient from blue to red indicates the increasing abundance of metabolite expression.

Supplementary Figure 8. Heat map of differentially expressed metabolites between the FHN group and FNF group. The horizontal axis represents sample names, and the vertical axis represents differentially expressed metabolites. The color gradient from blue to red indicates the increasing abundance of metabolite expression.

Supplementary Figure 9. Heat map of differentially expressed metabolites between the FHN group and sclerosis group. The horizontal axis represents sample names, and the vertical axis represents differentially expressed metabolites. The color gradient from blue to red indicates the increasing abundance of metabolite expression.

Supplementary Figure 10. Volcano plot for the selection of differentially expressed metabolites based on VIP and p-value. Red dots represent significantly upregulated metabolites in the experimental group, blue dots represent significantly downregulated metabolites, and gray dots represent non-significant differences. A represents the sclerosis group relative to the fracture group with, 28 upregulated and 16 downregulated metabolites identified. B represents the necrosis group relative to the fracture group with, 51 upregulated and 43 downregulated metabolites. C represents the necrotic group, with 14 upregulated and 17 downregulated metabolites identified relative to the sclerosis group around internal fixation.

Supplementary Figure 11. Correlation analysis of differentially expressed metabolites. Pearson correlation coefficient was used to measure the linear relationship between two metabolites. Red indicates positive correlation, and blue indicates negative correlation.

Supplementary Figure 12. Correlation between differentially expressed proteins and metabolites is meticulously delineated, where each row and column corresponds to unique metabolites and proteins, respectively. In the visualization, a red hue indicates a positive correlation, whereas blue signifies a negative correlation, with the intensity of the color denoting the strength of the correlation. Smaller circle diameters imply correlations nearing zero. *** highlight correlations with a p-value less than 0.001, ** denote a p-value less than 0.01, and * represents a p-value less than 0.05, signifying statistical significance.

Supplementary Figure 13. KGML network diagram between sclerosis and FNF groups illustrates differential proteins and metabolites. Squares indicate pathways, triangles represent proteins, and circles denote metabolites. Red is used to indicate upregulated proteins or metabolites, whereas blue marks those that are downregulated. The “Degree” signifies the number of connections among them.

Supplementary Figure 14. The KGML network diagram showcasing differences between FHN and FNF groups employs squares to depict pathways, triangles for proteins, and circles for metabolites. Upregulation is represented in red, and downregulation in blue, with the “Degree” indicating the interconnectedness among these entities.

Supplementary Figure 15. The KGML network diagram illustrating differential proteins and metabolites between FHN (femoral head necrosis) and sclerosis groups serves as a prime example. Within this diagram, squares signify pathways, triangles denote proteins, and circles represent metabolites. Red is used to indicate upregulated proteins or metabolites, contrasting with blue, which signifies those that are downregulated. The “Degree” quantifies the extent of interconnectivity among these entities, providing a comprehensive overview of the intricate relationships within the network.
