# Supplementary material for: Integrated proteomics and metabolomics analysis of sclerosis-related proteins and femoral head necrosis following internal fixation of femoral neck fractures
Source: Sci Rep. 2024 Jun 8;14:13207. doi: 10.1038/s41598-024-63837-8 (PMC11162501; doi:10.1038/s41598-024-63837-8)
Supplement: Supplementary file 18 — Supplementary Tables. [file 41598_2024_63837_MOESM18_ESM.docx]

Supplementary table 1. Principal component analysis (PCA) covariance between groups.

|  | A1 | A2 | A3 | C1 | C2 | C3 | B1 | B2 | B3 |
| --- | --- | --- | --- | --- | --- | --- | --- | --- | --- |
| A1 | 1 | 0.882 | 0.830 | 0.727 | 0.638 | 0.637 | 0.682 | 0.746 | 0.747 |
| A2 | 0.882 | 1 | 0.790 | 0.694 | 0.613 | 0.590 | 0.672 | 0.754 | 0.743 |
| A3 | 0.830 | 0.790 | 1 | 0.680 | 0.632 | 0.653 | 0.703 | 0.714 | 0.697 |
| C1 | 0.727 | 0.694 | 0.680 | 1 | 0.870 | 0.893 | 0.837 | 0.788 | 0.768 |
| C2 | 0.638 | 0.613 | 0.632 | 0.870 | 1 | 0.905 | 0.825 | 0.689 | 0.642 |
| C3 | 0.637 | 0.590 | 0.653 | 0.893 | 0.905 | 1 | 0.838 | 0.688 | 0.641 |
| B1 | 0.682 | 0.672 | 0.703 | 0.837 | 0.825 | 0.838 | 1 | 0.742 | 0.698 |
| B2 | 0.746 | 0.754 | 0.714 | 0.788 | 0.689 | 0.688 | 0.742 | 1 | 0.902 |
| B3 | 0.747 | 0.743 | 0.697 | 0.768 | 0.642 | 0.641 | 0.698 | 0.902 | 1 |

A: FNF group; B: Sclerotic group; C: FHN group.

Supplementary table 2. Differential expression of proteins in sclerosis samples compared to FNF samples

| Gene Name | Protein IDs | Peptides | Unique peptides | Sequence coverage [%] | Mol. weight [kDa] | P-value | FC |
| --- | --- | --- | --- | --- | --- | --- | --- |
| IGHV5-51 | A0A0C4DH38 | 3 | 1 | 34.2 | 12.674 | 0.0001 | inf |
| QSOX1 | O00391 | 7 | 7 | 10.7 | 82.577 | 0.0000 | inf |
| ISLR | O14498 | 5 | 5 | 15 | 45.997 | 0.0023 | inf |
| P4HA2 | O15460 | 9 | 9 | 27.5 | 60.901 | 0.0003 | inf |
| GLS | O94925 | 4 | 4 | 10.2 | 73.46 | 0.0000 | inf |
| IGHV2-5 | P01817 | 2 | 2 | 31.9 | 13.231 | 0.0001 | inf |
| SERPINA5 | P05154 | 2 | 2 | 5.7 | 45.674 | 0.0012 | inf |
| COL11A1 | P12107 | 3 | 3 | 2.2 | 181.06 | 0.0006 | inf |
| ACP5 | P13686 | 4 | 4 | 20.3 | 36.598 | 0.0001 | inf |
| FOLR2 | P14207;P41439 | 4 | 4 | 21.6 | 29.279 | 0.0000 | inf |
| GLB1 | P16278 | 6 | 6 | 13.6 | 76.074 | 0.0022 | inf |
| ITGB5 | P18084 | 3 | 3 | 4.3 | 88.053 | 0.0000 | inf |
| TNXB | P22105;Q16473 | 18 | 18 | 11.8 | 458.38 | 0.0002 | inf |
| IGFBP4 | P22692 | 4 | 4 | 20.9 | 27.934 | 0.0002 | inf |
| PROZ | P22891 | 3 | 3 | 7.2 | 44.743 | 0.0000 | inf |
| IGHV1-2 | P23083 | 4 | 3 | 35 | 13.085 | 0.0001 | inf |
| MST1 | P26927;Q2TV78 | 5 | 5 | 9.8 | 80.319 | 0.0000 | inf |
| CRABP2 | P29373 | 2 | 2 | 17.4 | 15.693 | 0.0000 | inf |
| GBP1 | P32455;Q9H0R5 | 12 | 10 | 24 | 67.93 | 0.0101 | inf |
| THBS2 | P35442 | 25 | 22 | 29.4 | 129.99 | 0.0083 | inf |
| CFHR2 | P36980 | 8 | 4 | 38.9 | 30.65 | 0.0000 | inf |
| CTSK | P43235 | 6 | 6 | 35 | 36.966 | 0.0026 | inf |
| MMP13 | P45452 | 5 | 5 | 15.3 | 53.819 | 0.0016 | inf |
| ME1 | P48163 | 8 | 8 | 26.9 | 64.149 | 0.0002 | inf |
| PITPNB | P48739 | 5 | 3 | 27.7 | 31.54 | 0.0000 | inf |
| BCAT1 | P54687 | 4 | 4 | 14.5 | 42.966 | 0.0004 | inf |
| SEC13 | P55735 | 8 | 8 | 39.4 | 35.54 | 0.0000 | inf |
| CCL18 | P55774 | 2 | 2 | 24.7 | 9.8487 | 0.0021 | inf |
| HLA-DRB3 | P79483 | 5 | 2 | 24.1 | 29.962 | 0.0000 | inf |
| DAB2 | P98082 | 3 | 3 | 4.7 | 82.447 | 0.0000 | inf |
| FBLN2 | P98095 | 6 | 6 | 6.2 | 126.57 | 0.0003 | inf |
| HDLBP | Q00341 | 21 | 21 | 24 | 141.45 | 0.0000 | inf |
| CDK17 | Q00537 | 2 | 1 | 3.3 | 59.582 | 0.0000 | inf |
| KHDRBS1 | Q07666 | 6 | 5 | 16 | 48.227 | 0.0011 | inf |
| PPP3CA | Q08209 | 3 | 3 | 8.6 | 58.687 | 0.0000 | inf |
| PAK2 | Q13177 | 4 | 4 | 15.6 | 58.042 | 0.0001 | inf |
| CTTN | Q14247 | 6 | 6 | 17.8 | 61.585 | 0.0000 | inf |
| EEA1 | Q15075 | 15 | 15 | 15 | 162.46 | 0.0001 | inf |
| CD276 | Q5ZPR3 | 2 | 2 | 6.7 | 57.235 | 0.0000 | inf |
| CD109 | Q6YHK3 | 6 | 6 | 6.6 | 161.69 | 0.0007 | inf |
| GPX8 | Q8TED1 | 3 | 3 | 11 | 23.881 | 0.0000 | inf |
| PALLD | Q8WX93 | 5 | 5 | 4 | 150.56 | 0.0000 | inf |
| LPP | Q93052 | 6 | 6 | 15.2 | 65.746 | 0.0000 | inf |
| FKBP10 | Q96AY3 | 17 | 16 | 35.4 | 64.244 | 0.0000 | inf |
| GPX7 | Q96SL4 | 2 | 2 | 19.3 | 20.996 | 0.0001 | inf |
| TM9SF2 | Q99805 | 4 | 4 | 6.2 | 75.775 | 0.0001 | inf |
| LMF2 | Q9BU23 | 3 | 3 | 7.2 | 79.697 | 0.0000 | inf |
| BDH2 | Q9BUT1 | 2 | 2 | 9 | 26.724 | 0.0000 | inf |
| TPPP3 | Q9BW30 | 4 | 4 | 21.6 | 18.985 | 0.0008 | inf |
| CRTAC1 | Q9NQ79 | 9 | 9 | 20.9 | 71.42 | 0.0014 | inf |
| MRC2 | Q9UBG0 | 24 | 24 | 25.8 | 166.67 | 0.0004 | inf |
| SWAP70 | Q9UH65 | 2 | 2 | 3.2 | 68.997 | 0.0000 | inf |
| RTRAF | Q9Y224 | 2 | 2 | 10.2 | 28.068 | 0.0003 | inf |
| CLEC11A | Q9Y240 | 3 | 3 | 11.8 | 35.694 | 0.0006 | inf |
| NOP58 | Q9Y2X3 | 3 | 3 | 8.9 | 59.578 | 0.0000 | inf |
| SNX9 | Q9Y5X1 | 4 | 4 | 8.9 | 66.591 | 0.0000 | inf |
| PCOLCE | Q15113 | 16 | 16 | 51.2 | 47.972 | 0.0072 | 27.7958 |
| AIFM1 | O95831 | 9 | 9 | 20.6 | 66.9 | 0.0034 | 15.6763 |
| MAP4 | P27816 | 23 | 23 | 28.2 | 121 | 0.0002 | 13.9299 |
| COL3A1 | P02461 | 11 | 11 | 9.2 | 138.56 | 0.0108 | 11.2845 |
| F12 | P00748 | 10 | 10 | 29.4 | 67.791 | 0.0147 | 10.4910 |
| MYOF | Q9NZM1 | 29 | 29 | 20.3 | 234.71 | 0.0162 | 9.1612 |
| COL6A2 | P12110 | 29 | 29 | 35.4 | 108.58 | 0.0113 | 7.3311 |
| P4HA1 | P13674 | 11 | 11 | 24.5 | 61.049 | 0.0038 | 7.0245 |
| COL6A1 | P12109 | 44 | 43 | 51.9 | 108.53 | 0.0190 | 6.4188 |
| TGFBI | Q15582 | 35 | 35 | 69.3 | 74.68 | 0.0219 | 5.9498 |
| COL6A3 | P12111 | 160 | 160 | 58.9 | 343.67 | 0.0085 | 5.7898 |
| PLS3 | P13797;Q14651 | 34 | 27 | 67 | 70.81 | 0.0120 | 5.7328 |
| GPNMB | Q14956 | 4 | 4 | 7.7 | 63.922 | 0.0283 | 5.5458 |
| FBLN1 | P23142 | 19 | 19 | 38.7 | 77.213 | 0.0173 | 5.3832 |
| IGHV3-49 | A0A0A0MS15 | 3 | 2 | 26.1 | 13.056 | 0.0023 | 5.1685 |
| KLKB1 | P03952 | 21 | 20 | 38.4 | 71.369 | 0.0044 | 5.0494 |
| DBN1 | Q16643 | 5 | 5 | 13.9 | 71.428 | 0.0063 | 4.8168 |
| CORO1B | Q9BR76 | 9 | 9 | 29 | 54.234 | 0.0094 | 4.6330 |
| C8B | P07358 | 22 | 22 | 54.1 | 67.046 | 0.0319 | 4.6075 |
| CPB2 | Q96IY4 | 11 | 11 | 38.3 | 48.424 | 0.0224 | 4.4754 |
| IGHV4-34 | P06331 | 3 | 2 | 37.4 | 13.815 | 0.0055 | 4.3411 |
| FETUB | Q9UGM5 | 5 | 5 | 23 | 42.054 | 0.0097 | 4.1486 |
| CFH | P08603 | 59 | 50 | 61.3 | 139.09 | 0.0188 | 4.0346 |
| CD5L | O43866 | 15 | 15 | 55.9 | 38.087 | 0.0454 | 3.9495 |
| APOB | P04114 | 196 | 196 | 51.8 | 515.6 | 0.0265 | 3.9060 |
| COPE | O14579 | 9 | 9 | 52.3 | 34.482 | 0.0075 | 3.6293 |
| CRIP2 | P52943 | 5 | 5 | 49.5 | 22.492 | 0.0018 | 3.5679 |
| AFM | P43652 | 17 | 17 | 37.1 | 69.068 | 0.0157 | 3.5484 |
| SERPINA4 | P29622 | 14 | 14 | 45.7 | 48.541 | 0.0390 | 3.4848 |
| LRRC59 | Q96AG4 | 7 | 7 | 30 | 34.93 | 0.0304 | 3.3886 |
| C8A | P07357 | 21 | 21 | 54.8 | 65.163 | 0.0163 | 3.3791 |
| MVP | Q14764 | 29 | 29 | 49.4 | 99.326 | 0.0419 | 3.3647 |
| THY1 | P04216 | 4 | 4 | 24.8 | 17.935 | 0.0153 | 3.3010 |
| IGLV3-25 | P01717 | 5 | 2 | 36.6 | 12.011 | 0.0322 | 3.2934 |
| RTCB | Q9Y3I0 | 10 | 10 | 28.7 | 55.21 | 0.0499 | 3.1683 |
| ARCN1 | P48444 | 15 | 15 | 38.2 | 57.21 | 0.0296 | 3.1319 |
| EFEMP1 | Q12805 | 15 | 15 | 44.2 | 54.64 | 0.0435 | 3.1167 |
| TF | P02787 | 59 | 58 | 70.8 | 77.063 | 0.0010 | 3.0068 |
| IGHV3-72 | A0A0B4J1Y9 | 4 | 3 | 40.3 | 13.203 | 0.0000 | 2.9395 |
| KNG1 | P01042 | 26 | 26 | 33.9 | 71.957 | 0.0008 | 2.8668 |
| COL5A1 | P20908 | 11 | 11 | 9.2 | 183.56 | 0.0134 | 2.8154 |
| ITIH1 | P19827 | 14 | 14 | 26 | 101.39 | 0.0259 | 2.6167 |
| SERPINA7 | P05543 | 9 | 9 | 33.3 | 46.324 | 0.0110 | 2.6019 |
| A2M | P01023 | 75 | 64 | 66.1 | 163.29 | 0.0254 | 2.5534 |
| DSTN | P60981 | 6 | 6 | 39.4 | 18.506 | 0.0045 | 2.4855 |
| ADIPOQ | Q15848 | 2 | 2 | 15.6 | 26.413 | 0.0076 | 2.4760 |
| RBP4 | P02753 | 8 | 8 | 45.8 | 23.01 | 0.0334 | 2.4733 |
| PICALM | Q13492 | 6 | 6 | 12.1 | 70.754 | 0.0118 | 2.4643 |
| SNX6 | Q9UNH7 | 5 | 4 | 14.3 | 46.648 | 0.0073 | 2.4557 |
| CLEC3B | P05452 | 9 | 9 | 47 | 22.537 | 0.0425 | 2.4321 |
| APOD | P05090 | 7 | 7 | 34.9 | 21.275 | 0.0073 | 2.4244 |
| HNRNPR | O43390 | 9 | 6 | 17.2 | 70.942 | 0.0262 | 2.4109 |
| CAPN2 | P17655 | 14 | 14 | 28.3 | 79.994 | 0.0204 | 2.4074 |
| APOH | P02749 | 17 | 17 | 55.4 | 38.298 | 0.0159 | 2.3861 |
| HPX | P02790 | 27 | 27 | 66.2 | 51.676 | 0.0049 | 2.3418 |
|  | P0DOX2 | 17 | 3 | 53.4 | 48.934 | 0.0085 | 2.3364 |
| RPL27 | P61353 | 5 | 5 | 43.4 | 15.798 | 0.0395 | 2.3285 |
| GC | P02774 | 38 | 38 | 77.6 | 52.917 | 0.0003 | 2.3126 |
| AKR1B1 | P15121 | 12 | 12 | 57.3 | 35.853 | 0.0054 | 2.2945 |
| PDLIM5 | Q96HC4 | 11 | 11 | 27.5 | 63.944 | 0.0306 | 2.2656 |
| APOL1 | O14791 | 8 | 8 | 27.4 | 43.974 | 0.0250 | 2.2599 |
| CAPG | P40121 | 13 | 13 | 47.4 | 38.498 | 0.0493 | 2.2414 |
| HCLS1 | P14317 | 10 | 10 | 25.9 | 54.013 | 0.0213 | 2.2377 |
| PFKP | Q01813 | 7 | 5 | 14.8 | 85.595 | 0.0251 | 2.2375 |
| RPS11 | P62280 | 12 | 12 | 57.6 | 18.431 | 0.0361 | 2.1606 |
| ERAP1 | Q9NZ08 | 20 | 20 | 28.5 | 107.23 | 0.0421 | 2.1436 |
| VAPB | O95292 | 4 | 3 | 24.3 | 27.228 | 0.0433 | 2.1269 |
| IGHG1 | P0DOX5 | 25 | 13 | 56.3 | 49.328 | 0.0181 | 2.1196 |
| PKM | P14618 | 39 | 38 | 75.3 | 57.936 | 0.0076 | 2.0897 |
| A1BG | P04217 | 17 | 17 | 58.2 | 54.253 | 0.0142 | 2.0634 |
| HK3 | P52790 | 18 | 16 | 27.8 | 99.024 | 0.0148 | 2.0560 |
| EPB41L2 | O43491 | 15 | 13 | 20.9 | 112.59 | 0.0317 | 2.0470 |
| IGHV3-43D | P0DP04 | 3 | 2 | 34.7 | 13.017 | 0.0404 | 2.0248 |
| NUCB1 | Q02818 | 10 | 10 | 28 | 53.879 | 0.0499 | 2.0174 |
| GART | P22102 | 7 | 7 | 10.7 | 107.77 | 0.0200 | 1.9929 |
| C3 | P01024 | 111 | 111 | 73.5 | 187.15 | 0.0411 | 1.9464 |
| TXNDC5 | Q8NBS9 | 17 | 17 | 54.2 | 47.628 | 0.0255 | 1.8998 |
| ANPEP | P15144 | 30 | 30 | 37.8 | 109.54 | 0.0037 | 1.8246 |
| LMAN1 | P49257 | 15 | 15 | 35.7 | 57.548 | 0.0402 | 1.8187 |
| ANXA4 | P09525 | 15 | 15 | 54.5 | 35.882 | 0.0114 | 1.8121 |
| PCBP2 | Q15366 | 7 | 5 | 29 | 38.58 | 0.0368 | 1.8013 |
| PLOD3 | O60568 | 6 | 6 | 11.5 | 84.784 | 0.0211 | 1.7800 |
| API5 | Q9BZZ5 | 4 | 4 | 10.5 | 59.004 | 0.0315 | 1.7569 |
| AGT | P01019 | 13 | 13 | 39.4 | 53.154 | 0.0144 | 1.7301 |
| SRPRB | Q9Y5M8 | 5 | 5 | 25.8 | 29.702 | 0.0185 | 1.7162 |
| CFI | P05156;CON__Q32PI4 | 18 | 18 | 37.2 | 65.75 | 0.0088 | 1.7110 |
| AP2M1 | Q96CW1 | 8 | 8 | 22.8 | 49.654 | 0.0346 | 1.6443 |
| GSN | P06396 | 36 | 17 | 53.6 | 85.696 | 0.0069 | 1.5940 |
| ALDH7A1 | P49419 | 7 | 7 | 17.1 | 58.486 | 0.0388 | 1.5899 |
| SEPTIN11 | Q9NVA2;Q9P0V9 | 12 | 7 | 37.5 | 49.398 | 0.0446 | 1.4334 |
| PPP1CB | P62140 | 12 | 5 | 54.7 | 37.186 | 0.0337 | 1.3711 |
| VAMP3 | Q15836;P63027 | 3 | 3 | 45 | 11.309 | 0.0416 | 1.3432 |
| AP2S1 | P53680 | 3 | 3 | 19 | 17.018 | 0.0323 | 1.2322 |
| PSMA1 | P25786 | 12 | 12 | 53.2 | 29.555 | 0.0468 | 0.7422 |
| GAPDH | P04406;O14556 | 20 | 20 | 78.2 | 36.053 | 0.0417 | 0.7093 |
| PSMA7 | O14818;Q8TAA3 | 10 | 10 | 52.8 | 27.887 | 0.0479 | 0.7086 |
| ATP5F1D | P30049 | 3 | 3 | 37.5 | 17.49 | 0.0141 | 0.7035 |
| YWHAE | P62258 | 21 | 19 | 72.5 | 29.174 | 0.0330 | 0.6893 |
| FH | P07954 | 12 | 12 | 44.1 | 54.636 | 0.0222 | 0.6734 |
| NME1 | P15531 | 10 | 5 | 70.4 | 17.149 | 0.0182 | 0.6693 |
| LANCL1 | O43813 | 4 | 4 | 11 | 45.283 | 0.0113 | 0.6563 |
| MLEC | Q14165 | 4 | 4 | 17.1 | 32.233 | 0.0242 | 0.6518 |
| GSTM2 | P28161 | 7 | 3 | 40.4 | 25.744 | 0.0238 | 0.5650 |
| LDHB | P07195 | 20 | 19 | 61.4 | 36.638 | 0.0366 | 0.5236 |
| RALB | P11234 | 4 | 2 | 24.3 | 23.408 | 0.0225 | 0.5154 |
| CPNE3 | O75131 | 11 | 11 | 27.7 | 60.13 | 0.0284 | 0.4993 |
| UROD | P06132 | 9 | 9 | 51 | 40.786 | 0.0118 | 0.4842 |
| RAP1B | P61224;A6NIZ1;O95716 | 12 | 5 | 62 | 20.825 | 0.0294 | 0.4835 |
| OXSR1 | O95747 | 8 | 8 | 21.4 | 58.022 | 0.0037 | 0.4764 |
| USP15 | Q9Y4E8 | 8 | 8 | 10.5 | 112.42 | 0.0478 | 0.4633 |
| RAB8B | Q92930 | 3 | 2 | 17.9 | 23.584 | 0.0372 | 0.4557 |
| HSPA1A | P0DMV8;P0DMV9 | 26 | 13 | 47.1 | 70.051 | 0.0008 | 0.4437 |
| BOLA2 | Q9H3K6 | 4 | 4 | 61.6 | 10.116 | 0.0111 | 0.4410 |
| PKLR | P30613 | 10 | 9 | 26.1 | 61.829 | 0.0248 | 0.3955 |
| EHD1 | Q9H4M9 | 20 | 13 | 52.4 | 60.626 | 0.0151 | 0.2943 |
| SERPINB1 | P30740;O75830 | 23 | 22 | 60.2 | 42.741 | 0.0233 | 0.2859 |
| CAVIN2 | O95810 | 7 | 7 | 20.2 | 47.173 | 0.0254 | 0.2664 |
| TUBA4A | P68366 | 23 | 4 | 60 | 49.924 | 0.0334 | 0.2566 |
| ITIH3 | Q06033 | 12 | 12 | 22 | 99.848 | 0.0082 | 0.2330 |
| PF4 | P02776;P10720 | 4 | 4 | 35.6 | 10.845 | 0.0061 | 0.2066 |
| S100A9 | P06702 | 13 | 13 | 86 | 13.242 | 0.0224 | 0.1809 |
| RPIA | P49247 | 7 | 7 | 28.9 | 33.269 | 0.0016 | 0.1727 |
| LCN2 | P80188 | 9 | 9 | 58.1 | 22.588 | 0.0140 | 0.1700 |
| SPP2 | Q13103 | 6 | 6 | 27 | 24.337 | 0.0045 | 0.1578 |
| MPO | P05164 | 47 | 42 | 61.5 | 83.868 | 0.0458 | 0.1571 |
| NAP1L1 | P55209 | 5 | 4 | 17.4 | 45.374 | 0.0008 | 0.1435 |
| NCL | P19338 | 13 | 13 | 22.5 | 76.613 | 0.0240 | 0.0990 |
| ITGA2B | P08514 | 28 | 28 | 37.7 | 113.38 | 0.0110 | 0.0832 |
| RAB27B | O00194 | 3 | 2 | 14.7 | 24.608 | 0.0000 | 0.0000 |
| FCN1 | O00602 | 3 | 3 | 11.3 | 35.078 | 0.0000 | 0.0000 |
| PFAS | O15067 | 3 | 3 | 3.7 | 144.73 | 0.0001 | 0.0000 |
| PGLYRP1 | O75594 | 4 | 4 | 33.2 | 21.731 | 0.0002 | 0.0000 |
| PLPBP | O94903 | 3 | 3 | 15.3 | 30.344 | 0.0008 | 0.0000 |
| ENDOD1 | O94919 | 2 | 2 | 5.4 | 55.016 | 0.0002 | 0.0000 |
| LSM8 | O95777 | 2 | 2 | 27.1 | 10.403 | 0.0009 | 0.0000 |
| KRAS | P01116;P01112;P01111 | 4 | 4 | 24.9 | 21.656 | 0.0000 | 0.0000 |
| ARG1 | P05089 | 4 | 4 | 18.9 | 34.735 | 0.0000 | 0.0000 |
| RNASE2 | P10153 | 3 | 3 | 19.9 | 18.354 | 0.0000 | 0.0000 |
| LAMP1 | P11279 | 2 | 2 | 6.5 | 44.882 | 0.0011 | 0.0000 |
| MYL4 | P12829 | 4 | 3 | 27.9 | 21.564 | 0.0005 | 0.0000 |
| H1-2 | P16403 | 9 | 2 | 27.2 | 21.364 | 0.0004 | 0.0000 |
| IBSP | P21815 | 1 | 1 | 2.8 | 35.147 | 0.0000 | 0.0000 |
| S100P | P25815 | 2 | 2 | 24.2 | 10.4 | 0.0002 | 0.0000 |
| HMGB2 | P26583 | 6 | 4 | 30.1 | 24.033 | 0.0023 | 0.0000 |
| CEACAM8 | P31997 | 2 | 2 | 9.7 | 38.153 | 0.0001 | 0.0000 |
| CDA | P32320 | 4 | 4 | 46.6 | 16.185 | 0.0000 | 0.0000 |
| FEN1 | P39748 | 3 | 3 | 10 | 42.592 | 0.0001 | 0.0000 |
| GNAQ | P50148 | 4 | 2 | 17.3 | 42.142 | 0.0000 | 0.0000 |
| RPS6KA3 | P51812;Q15418 | 2 | 1 | 3.9 | 83.735 | 0.0001 | 0.0000 |
| UBE2M | P61081 | 2 | 2 | 10.4 | 20.9 | 0.0000 | 0.0000 |
| RBX1 | P62877 | 2 | 2 | 24.1 | 12.274 | 0.0000 | 0.0000 |
| EML5 | Q05BV3 | 2 | 2 | 1.4 | 219.42 | 0.0000 | 0.0000 |
| BST1 | Q10588 | 5 | 5 | 21.1 | 35.724 | 0.0015 | 0.0000 |
| CTBP1 | Q13363;P56545 | 4 | 4 | 13.4 | 47.535 | 0.0003 | 0.0000 |
| LGALSL | Q3ZCW2 | 2 | 2 | 14.5 | 18.986 | 0.0004 | 0.0000 |
| CD177 | Q8N6Q3 | 3 | 3 | 8 | 46.363 | 0.0003 | 0.0000 |
| RFESD | Q8TAC1 | 2 | 2 | 18.5 | 17.762 | 0.0001 | 0.0000 |
| CMBL | Q96DG6 | 2 | 2 | 8.2 | 28.048 | 0.0001 | 0.0000 |
| DCUN1D1 | Q96GG9 | 4 | 4 | 18.9 | 30.124 | 0.0001 | 0.0000 |
| UBAC1 | Q9BSL1 | 6 | 6 | 20 | 45.338 | 0.0001 | 0.0000 |
| SH3GLB2 | Q9NR46 | 3 | 2 | 7.3 | 43.973 | 0.0000 | 0.0000 |
| PRG3 | Q9Y2Y8 | 2 | 2 | 12.4 | 25.405 | 0.0000 | 0.0000 |

Supplementary table 3.Differential expression of proteins in FHN samples compared to FNF samples

| **Gene Name** | **Protein IDs** | **Peptides** | **Unique peptides** | **Sequence coverage [%]** | **Mol. weight [kDa]** | **P-value** | **FC** |
| --- | --- | --- | --- | --- | --- | --- | --- |
| IGKV2D-40 | P01614;A0A087WW87 | 3 | 1 | 28.1 | 13.31 | 0.0000 | inf |
| IGKV6D-21 | A0A0A0MT36; | 2 | 2 | 28.9 | 12.34 | 0.0000 | inf |
| IGHV2-26 | A0A0B4J1V2 | 2 | 2 | 17.6 | 13.182 | 0.0001 | inf |
| IGHV3-73 | A0A0B4J1V6 | 4 | 2 | 35.3 | 12.858 | 0.0000 | inf |
| IGHV3-43 | A0A0B4J1X8 | 3 | 2 | 34.7 | 13.077 | 0.0000 | inf |
| IGHV3-38 | A0A0C4DH36 | 2 | 2 | 19 | 12.758 | 0.0000 | inf |
| IGHV5-51 | A0A0C4DH38 | 3 | 1 | 34.2 | 12.674 | 0.0000 | inf |
| UBA6 | A0AVT1 | 3 | 3 | 4.1 | 117.97 | 0.0011 | inf |
| QSOX1 | O00391 | 7 | 7 | 10.7 | 82.577 | 0.0001 | inf |
| VWA5A | O00534 | 5 | 5 | 10.7 | 86.488 | 0.0000 | inf |
| NOP56 | O00567 | 4 | 4 | 10.4 | 66.049 | 0.0000 | inf |
| ISLR | O14498 | 5 | 5 | 15 | 45.997 | 0.0017 | inf |
| PLSCR1 | O15162 | 4 | 4 | 15.7 | 35.049 | 0.0000 | inf |
| PMM2 | O15305 | 4 | 4 | 23.6 | 28.082 | 0.0000 | inf |
| P4HA2 | O15460 | 9 | 9 | 27.5 | 60.901 | 0.0001 | inf |
| DYNC1LI2 | O43237 | 5 | 5 | 18.3 | 54.099 | 0.0001 | inf |
| PAPSS1 | O43252 | 3 | 3 | 7.1 | 70.832 | 0.0000 | inf |
| AHCYL2 | Q96HN2;O43865 | 3 | 3 | 5.9 | 66.72 | 0.0000 | inf |
| SPAG9 | O60271 | 3 | 3 | 3.5 | 146.2 | 0.0000 | inf |
| ACSL4 | O60488;O95573 | 3 | 3 | 6.9 | 79.187 | 0.0000 | inf |
| KPNA6 | O60684;O15131 | 2 | 2 | 6.9 | 60.029 | 0.0000 | inf |
| CTNND1 | O60716 | 4 | 4 | 6.4 | 108.17 | 0.0001 | inf |
| DNAJC13 | O75165 | 3 | 3 | 2 | 254.41 | 0.0001 | inf |
| NDUFS2 | O75306 | 3 | 3 | 9.1 | 52.545 | 0.0000 | inf |
| SNRNP200 | O75643 | 7 | 7 | 5.5 | 244.5 | 0.0001 | inf |
| ZMPSTE24 | O75844 | 3 | 3 | 8.4 | 54.812 | 0.0000 | inf |
| DYSF | O75923 | 4 | 4 | 3 | 237.29 | 0.0004 | inf |
| TOMM70 | O94826 | 6 | 6 | 17.3 | 67.454 | 0.0004 | inf |
| SEC24D | O94855 | 4 | 4 | 6.1 | 113.01 | 0.0000 | inf |
| UFL1 | O94874 | 5 | 5 | 9.7 | 89.594 | 0.0000 | inf |
| GLS | O94925 | 4 | 4 | 10.2 | 73.46 | 0.0001 | inf |
| LUC7L3 | O95232 | 3 | 3 | 9 | 51.466 | 0.0000 | inf |
| PAPSS2 | O95340 | 5 | 5 | 11.9 | 69.5 | 0.0001 | inf |
| LYPLA2 | O95372 | 4 | 4 | 33.3 | 24.737 | 0.0000 | inf |
| PGM3 | O95394 | 4 | 4 | 12 | 59.851 | 0.0001 | inf |
| SGPL1 | O95470 | 3 | 3 | 8.6 | 63.523 | 0.0000 | inf |
| EFEMP2 | O95967 | 2 | 2 | 5.9 | 49.405 | 0.0011 | inf |
| ACTL6A | O96019 | 3 | 3 | 13.5 | 47.46 | 0.0001 | inf |
| OAS1 | P00973 | 8 | 8 | 28 | 46.028 | 0.0004 | inf |
| IGKV1-33 | P01594;P01593 | 2 | 2 | 34.2 | 12.848 | 0.0000 | inf |
| IGKV1-5 | P01602 | 3 | 2 | 24.8 | 12.781 | 0.0001 | inf |
| IGLV1-40 | P01703 | 3 | 2 | 39 | 12.301 | 0.0000 | inf |
| IGLV3-27 | P01718 | 4 | 2 | 32.7 | 12.165 | 0.0000 | inf |
| IGHV2-5 | P01817 | 2 | 2 | 31.9 | 13.231 | 0.0000 | inf |
| FUCA1 | P04066 | 7 | 7 | 23 | 53.688 | 0.0003 | inf |
| GLA | P06280 | 4 | 4 | 13.5 | 48.766 | 0.0000 | inf |
| SERPINE2 | P07093 | 3 | 3 | 12.3 | 44.002 | 0.0001 | inf |
| GUSB | P08236 | 6 | 6 | 16.1 | 74.731 | 0.0000 | inf |
| PDHA1 | P08559 | 4 | 4 | 11 | 43.295 | 0.0000 | inf |
| PTPRC | P08575 | 4 | 4 | 4.2 | 147.48 | 0.0002 | inf |
| HCK | P08631 | 4 | 2 | 8.6 | 59.599 | 0.0000 | inf |
| ACAA1 | P09110 | 7 | 7 | 31.6 | 44.292 | 0.0001 | inf |
| CTSH | P09668 | 6 | 6 | 29.3 | 37.393 | 0.0001 | inf |
| IFIT1 | P09914 | 12 | 12 | 37.2 | 55.36 | 0.0000 | inf |
| UCHL1 | P09936 | 7 | 7 | 54.7 | 24.824 | 0.0015 | inf |
| SULT1A3 | P0DMM9;P0DMN0 | 9 | 4 | 46.1 | 34.196 | 0.0001 | inf |
| IGF2R | P11717 | 4 | 4 | 2.6 | 274.37 | 0.0000 | inf |
| HARS1 | P12081 | 7 | 6 | 21 | 57.41 | 0.0012 | inf |
| COL11A1 | P12107 | 3 | 3 | 2.2 | 181.06 | 0.0010 | inf |
| ACP5 | P13686 | 4 | 4 | 20.3 | 36.598 | 0.0001 | inf |
| GYS1 | P13807 | 2 | 2 | 5.6 | 83.785 | 0.0000 | inf |
| FOLR2 | P14207;P41439 | 4 | 4 | 21.6 | 29.279 | 0.0000 | inf |
| GLB1 | P16278 | 6 | 6 | 13.6 | 76.074 | 0.0001 | inf |
| GJA1 | P17302 | 5 | 5 | 20.9 | 43.008 | 0.0001 | inf |
| ITGB5 | P18084 | 3 | 3 | 4.3 | 88.053 | 0.0000 | inf |
| SRM | P19623 | 7 | 7 | 33.4 | 33.824 | 0.0000 | inf |
| NFKB1 | P19838 | 2 | 2 | 4 | 105.35 | 0.0000 | inf |
| MX2 | P20592 | 8 | 5 | 14.7 | 82.088 | 0.0000 | inf |
| ATP6V1C1 | P21283 | 4 | 4 | 13.4 | 43.941 | 0.0003 | inf |
| TNXB | P22105;Q16473 | 18 | 18 | 11.8 | 458.38 | 0.0014 | inf |
| IGFBP4 | P22692 | 4 | 4 | 20.9 | 27.934 | 0.0123 | inf |
| MRC1 | P22897 | 14 | 14 | 13.1 | 166.01 | 0.0000 | inf |
| IGHV1-2 | P23083 | 4 | 3 | 35 | 13.085 | 0.0000 | inf |
| DDX6 | P26196 | 3 | 3 | 9.5 | 54.416 | 0.0004 | inf |
| IVD | P26440 | 2 | 2 | 8.7 | 46.65 | 0.0012 | inf |
| MST1 | P26927;Q2TV78 | 5 | 5 | 9.8 | 80.319 | 0.0120 | inf |
| MAPK3 | P27361 | 9 | 5 | 33.8 | 43.135 | 0.0000 | inf |
| RPA1 | P27694 | 3 | 3 | 7.5 | 68.137 | 0.0013 | inf |
| CRABP2 | P29373 | 2 | 2 | 17.4 | 15.693 | 0.0001 | inf |
| CASP1 | P29466 | 7 | 7 | 23 | 45.158 | 0.0000 | inf |
| PML | P29590 | 5 | 5 | 6.7 | 97.55 | 0.0008 | inf |
| OAS2 | P29728 | 12 | 12 | 22.4 | 82.43 | 0.0006 | inf |
| LRPAP1 | P30533 | 3 | 3 | 12.3 | 41.465 | 0.0000 | inf |
| ALDH1B1 | P30837 | 6 | 5 | 13.3 | 57.206 | 0.0002 | inf |
| GBP1 | P32455;Q9H0R5 | 12 | 10 | 24 | 67.93 | 0.0023 | inf |
| GBP2 | P32456 | 4 | 2 | 10.5 | 67.208 | 0.0000 | inf |
| MPI | P34949 | 4 | 4 | 17.5 | 46.655 | 0.0009 | inf |
| CTNNB1 | P35222 | 5 | 5 | 12.2 | 85.496 | 0.0000 | inf |
| THBS2 | P35442 | 25 | 22 | 29.4 | 129.99 | 0.0001 | inf |
| CFHR2 | P36980 | 8 | 4 | 38.9 | 30.65 | 0.0001 | inf |
| COL15A1 | P39059 | 6 | 6 | 5.5 | 141.72 | 0.0000 | inf |
| MMP12 | P39900 | 11 | 11 | 28.3 | 54.001 | 0.0013 | inf |
| PTGDS | P41222 | 3 | 3 | 21.1 | 21.029 | 0.0001 | inf |
| CSK | P41240 | 5 | 5 | 14.2 | 50.704 | 0.0000 | inf |
| IARS1 | P41252 | 7 | 7 | 7.6 | 144.5 | 0.0000 | inf |
| STAT5A | P42229;P51692 | 2 | 2 | 3 | 90.646 | 0.0000 | inf |
| PRCP | P42785 | 6 | 6 | 17.7 | 55.799 | 0.0001 | inf |
| CTSK | P43235 | 6 | 6 | 35 | 36.966 | 0.0007 | inf |
| MMP13 | P45452 | 5 | 5 | 15.3 | 53.819 | 0.0000 | inf |
| RECQL | P46063 | 7 | 7 | 16.2 | 73.457 | 0.0000 | inf |
| PREP | P48147 | 5 | 5 | 13.4 | 80.699 | 0.0001 | inf |
| ME1 | P48163 | 8 | 8 | 26.9 | 64.149 | 0.0000 | inf |
| PITPNB | P48739 | 5 | 3 | 27.7 | 31.54 | 0.0000 | inf |
| ARRB1 | P49407 | 3 | 3 | 11.5 | 47.065 | 0.0000 | inf |
| CARS1 | P49589 | 5 | 5 | 8.2 | 85.472 | 0.0009 | inf |
| GZMK | P49863 | 2 | 2 | 12.5 | 28.882 | 0.0000 | inf |
| NT5C2 | P49902 | 3 | 3 | 10.2 | 64.969 | 0.0000 | inf |
| ENTPD1 | P49961 | 2 | 2 | 6.5 | 57.964 | 0.0000 | inf |
| SERPINB9 | P50453 | 10 | 8 | 38.8 | 42.403 | 0.0017 | inf |
| PPP5C | P53041 | 3 | 3 | 8.6 | 56.878 | 0.0000 | inf |
| SEC24C | P53992 | 5 | 5 | 6.8 | 118.32 | 0.0002 | inf |
| BCAT1 | P54687 | 4 | 4 | 14.5 | 42.966 | 0.0000 | inf |
| ALDH18A1 | P54886 | 6 | 6 | 11.2 | 87.301 | 0.0000 | inf |
| MFAP2 | P55001 | 2 | 2 | 10.4 | 20.825 | 0.0003 | inf |
| NCKAP1L | P55160 | 5 | 5 | 6.8 | 128.15 | 0.0000 | inf |
| ADAR | P55265 | 6 | 6 | 7.4 | 136.06 | 0.0005 | inf |
| SEC13 | P55735 | 8 | 8 | 39.4 | 35.54 | 0.0000 | inf |
| CCL18 | P55774 | 2 | 2 | 24.7 | 9.8487 | 0.0007 | inf |
| ATP5MF | P56134 | 3 | 3 | 39.4 | 10.918 | 0.0000 | inf |
| MARS1 | P56192 | 4 | 4 | 6.7 | 101.11 | 0.0000 | inf |
| SRP54 | P61011 | 2 | 2 | 6.5 | 55.704 | 0.0000 | inf |
| ABCE1 | P61221 | 6 | 6 | 18.5 | 67.314 | 0.0001 | inf |
| ATP6V0D1 | P61421 | 2 | 2 | 9.4 | 40.329 | 0.0000 | inf |
| CNBP | P62633 | 3 | 3 | 23.2 | 19.463 | 0.0005 | inf |
| UBE2I | P63279 | 3 | 3 | 27.8 | 18.007 | 0.0000 | inf |
| NOMO2 | Q5JPE7;Q15155;P69849 | 10 | 10 | 12.7 | 139.44 | 0.0000 | inf |
| LACTB | P83111 | 8 | 8 | 19.7 | 60.693 | 0.0002 | inf |
| DAB2 | P98082 | 3 | 3 | 4.7 | 82.447 | 0.0001 | inf |
| FBLN2 | P98095 | 6 | 6 | 6.2 | 126.57 | 0.0000 | inf |
| HDLBP | Q00341 | 21 | 21 | 24 | 141.45 | 0.0000 | inf |
| CDK17 | Q00537;Q14004;P06493;P50750;Q00536;Q00534;P24941;O94921;Q07002;Q00535;Q00526;P11802;Q96Q40 | 2 | 1 | 3.3 | 59.582 | 0.0000 | inf |
| CYP27A1 | Q02318 | 8 | 8 | 22 | 60.234 | 0.0027 | inf |
| TAP2 | Q03519;Q9NUT2;Q9NRK6 | 5 | 5 | 10.8 | 75.663 | 0.0007 | inf |
| PTPN12 | Q05209 | 3 | 3 | 6.3 | 88.105 | 0.0002 | inf |
| KHDRBS1 | Q07666 | 6 | 5 | 16 | 48.227 | 0.0000 | inf |
| KLC1 | Q07866;Q9H0B6;Q9NSK0 | 3 | 3 | 7 | 65.309 | 0.0000 | inf |
| PPP3CA | Q08209;P16298;P48454 | 3 | 3 | 8.6 | 58.687 | 0.0000 | inf |
| GALNT1 | Q10472;Q8IUC8 | 5 | 5 | 14.7 | 64.218 | 0.0000 | inf |
| TWF1 | Q12792 | 3 | 2 | 11.1 | 40.282 | 0.0000 | inf |
| SF3A3 | Q12874 | 2 | 2 | 6.6 | 58.848 | 0.0000 | inf |
| FLII | Q13045 | 5 | 5 | 5.8 | 144.75 | 0.0000 | inf |
| AIMP2 | Q13155 | 3 | 3 | 14.7 | 35.348 | 0.0000 | inf |
| PAK2 | Q13177;Q13153;O75914 | 4 | 4 | 15.6 | 58.042 | 0.0000 | inf |
| G3BP1 | Q13283 | 3 | 3 | 9 | 52.164 | 0.0000 | inf |
| KCNAB2 | Q13303 | 5 | 5 | 26.2 | 41 | 0.0000 | inf |
| FKBP5 | Q13451 | 4 | 4 | 13.1 | 51.212 | 0.0000 | inf |
| TCIRG1 | Q13488 | 10 | 10 | 17 | 92.967 | 0.0001 | inf |
| SQSTM1 | Q13501 | 3 | 3 | 11.6 | 47.687 | 0.0000 | inf |
| TUBB3 | Q13509 | 17 | 3 | 41.8 | 50.432 | 0.0006 | inf |
| CUL3 | Q13618 | 2 | 2 | 3.6 | 88.929 | 0.0002 | inf |
| CTTN | Q14247 | 6 | 6 | 17.8 | 61.585 | 0.0000 | inf |
| PDIA5 | Q14554 | 6 | 6 | 12.7 | 59.594 | 0.0000 | inf |
| MESD | Q14696 | 2 | 2 | 11.5 | 26.076 | 0.0000 | inf |
| KARS1 | Q15046 | 9 | 9 | 22.6 | 68.047 | 0.0001 | inf |
| EEA1 | Q15075 | 15 | 15 | 15 | 162.46 | 0.0000 | inf |
| SEC23B | Q15437 | 12 | 9 | 22.6 | 86.478 | 0.0133 | inf |
| SF3A1 | Q15459 | 2 | 2 | 3.4 | 88.885 | 0.0000 | inf |
| CSRP2 | Q16527 | 4 | 4 | 23.8 | 20.954 | 0.0003 | inf |
| MAPK14 | Q16539 | 5 | 5 | 21.7 | 41.293 | 0.0001 | inf |
| KYNU | Q16719 | 6 | 6 | 20.2 | 52.351 | 0.0003 | inf |
| PCK2 | Q16822;P35558 | 6 | 6 | 13.1 | 70.698 | 0.0005 | inf |
| WASHC4 | Q2M389 | 4 | 4 | 4.7 | 136.4 | 0.0000 | inf |
| ALDH1L2 | Q3SY69 | 6 | 6 | 11.7 | 101.74 | 0.0000 | inf |
| CMPK2 | Q5EBM0 | 8 | 8 | 26.3 | 49.447 | 0.0002 | inf |
| PITRM1 | Q5JRX3 | 5 | 5 | 7 | 117.41 | 0.0018 | inf |
| THEMIS2 | Q5TEJ8 | 3 | 3 | 8.2 | 72.048 | 0.0000 | inf |
| RNF213 | Q63HN8 | 6 | 6 | 1.9 | 591.4 | 0.0000 | inf |
| MAP1S | Q66K74 | 3 | 3 | 5.6 | 112.21 | 0.0000 | inf |
| STEAP4 | Q687X5 | 4 | 4 | 15 | 51.981 | 0.0000 | inf |
| PRPF8 | Q6P2Q9 | 8 | 8 | 5.4 | 273.6 | 0.0000 | inf |
| BZW1 | Q7L1Q6;Q9Y6E2 | 4 | 4 | 11.9 | 48.043 | 0.0000 | inf |
| ZC3HAV1 | Q7Z2W4 | 3 | 3 | 5.1 | 101.43 | 0.0000 | inf |
| POGLUT3 | Q7Z4H8 | 4 | 4 | 11.8 | 58.572 | 0.0000 | inf |
| APBB1IP | Q7Z5R6 | 3 | 3 | 7.1 | 73.182 | 0.0000 | inf |
| MTDH | Q86UE4 | 4 | 4 | 10.3 | 63.836 | 0.0000 | inf |
| STX12 | Q86Y82 | 2 | 2 | 12.3 | 31.642 | 0.0001 | inf |
| AEBP1 | Q8IUX7 | 6 | 6 | 7.5 | 130.93 | 0.0001 | inf |
| WDFY1 | Q8IWB7 | 3 | 3 | 10.5 | 46.323 | 0.0000 | inf |
| CCAR2 | Q8N163 | 5 | 5 | 10.7 | 102.9 | 0.0000 | inf |
| LRRC47 | Q8N1G4 | 3 | 3 | 8.4 | 63.472 | 0.0001 | inf |
| NPLOC4 | Q8TAT6 | 2 | 2 | 5.3 | 68.119 | 0.0002 | inf |
| MICAL1 | Q8TDZ2 | 4 | 4 | 6.7 | 117.87 | 0.0000 | inf |
| SCFD1 | Q8WVM8 | 2 | 2 | 5.1 | 72.379 | 0.0000 | inf |
| GIMAP1 | Q8WWP7 | 3 | 3 | 16 | 34.369 | 0.0000 | inf |
| PALLD | Q8WX93 | 5 | 5 | 4 | 150.56 | 0.0003 | inf |
| TM9SF4 | Q92544 | 4 | 4 | 9.5 | 74.518 | 0.0002 | inf |
| ELMO1 | Q92556 | 4 | 3 | 7.4 | 83.829 | 0.0000 | inf |
| DOCK2 | Q92608 | 5 | 5 | 4 | 211.95 | 0.0000 | inf |
| GCN1 | Q92616 | 9 | 9 | 6.1 | 292.75 | 0.0000 | inf |
| TFG | Q92734 | 4 | 4 | 17 | 43.447 | 0.0000 | inf |
| GLG1 | Q92896 | 7 | 7 | 8.2 | 134.55 | 0.0000 | inf |
| ATP6V0A1 | Q93050 | 2 | 2 | 3.1 | 96.412 | 0.0000 | inf |
| LPP | Q93052 | 6 | 6 | 15.2 | 65.746 | 0.0001 | inf |
| NCLN | Q969V3 | 5 | 5 | 14.2 | 62.974 | 0.0000 | inf |
| FKBP10 | Q96AY3 | 17 | 16 | 35.4 | 64.244 | 0.0001 | inf |
| GALM | Q96C23 | 6 | 6 | 28.7 | 37.765 | 0.0000 | inf |
| DCPS | Q96C86 | 4 | 4 | 19 | 38.608 | 0.0008 | inf |
| CTHRC1 | Q96CG8 | 3 | 3 | 11.5 | 26.224 | 0.0000 | inf |
| DDRGK1 | Q96HY6 | 2 | 2 | 11.1 | 35.61 | 0.0000 | inf |
| TM9SF2 | Q99805 | 4 | 4 | 6.2 | 75.775 | 0.0000 | inf |
| PRMT1 | Q99873;Q9NR22 | 6 | 6 | 18.1 | 42.461 | 0.0000 | inf |
| AKAP9 | Q99996 | 3 | 3 | 0.9 | 452.98 | 0.0009 | inf |
| LMF2 | Q9BU23 | 3 | 3 | 7.2 | 79.697 | 0.0000 | inf |
| HNRNPUL1 | Q9BUJ2 | 4 | 4 | 6.3 | 95.737 | 0.0000 | inf |
| BDH2 | Q9BUT1 | 2 | 2 | 9 | 26.724 | 0.0000 | inf |
| TPPP3 | Q9BW30 | 4 | 4 | 21.6 | 18.985 | 0.0001 | inf |
| AP1M1 | Q9BXS5;Q9Y6Q5 | 4 | 4 | 11.6 | 48.586 | 0.0000 | inf |
| EMILIN2 | Q9BXX0 | 9 | 9 | 11.7 | 115.69 | 0.0000 | inf |
| SEC11C | Q9BY50 | 4 | 4 | 20.3 | 21.542 | 0.0003 | inf |
| SIGLEC1 | Q9BZZ2 | 7 | 7 | 7.4 | 182.62 | 0.0001 | inf |
| TNKS1BP1 | Q9C0C2 | 3 | 3 | 2.9 | 181.79 | 0.0001 | inf |
| TOLLIP | Q9H0E2 | 2 | 2 | 9.9 | 30.281 | 0.0000 | inf |
| ACBD3 | Q9H3P7 | 4 | 4 | 11 | 60.593 | 0.0000 | inf |
| DNAJC5 | Q9H3Z4 | 2 | 2 | 14.6 | 22.149 | 0.0001 | inf |
| GNB4 | Q9HAV0 | 8 | 2 | 25.6 | 37.567 | 0.0002 | inf |
| ATP13A1 | Q9HD20 | 3 | 3 | 3.7 | 132.95 | 0.0003 | inf |
| TM9SF3 | Q9HD45 | 5 | 5 | 10.5 | 67.887 | 0.0000 | inf |
| CD209 | Q9NNX6;Q9H2X3 | 5 | 5 | 28 | 45.774 | 0.0004 | inf |
| CRTAC1 | Q9NQ79 | 9 | 9 | 20.9 | 71.42 | 0.0005 | inf |
| PDLIM7 | Q9NR12 | 4 | 4 | 10.7 | 49.844 | 0.0001 | inf |
| MXRA5 | Q9NR99 | 17 | 17 | 8.1 | 312.15 | 0.0001 | inf |
| FKBP11 | Q9NYL4 | 2 | 2 | 13.9 | 22.18 | 0.0002 | inf |
| CISD1 | Q9NZ45 | 1 | 1 | 12 | 12.199 | 0.0000 | inf |
| LMCD1 | Q9NZU5 | 7 | 7 | 24.1 | 40.832 | 0.0000 | inf |
| MRC2 | Q9UBG0 | 24 | 24 | 25.8 | 166.67 | 0.0000 | inf |
| FBLN5 | Q9UBX5 | 2 | 2 | 4.5 | 50.18 | 0.0001 | inf |
| CGGBP1 | Q9UFW8 | 1 | 1 | 6.6 | 18.82 | 0.0000 | inf |
| TES | Q9UGI8 | 5 | 5 | 14.3 | 47.996 | 0.0000 | inf |
| SWAP70 | Q9UH65 | 2 | 2 | 3.2 | 68.997 | 0.0000 | inf |
| LIMA1 | Q9UHB6 | 3 | 3 | 5.9 | 85.225 | 0.0000 | inf |
| SRP68 | Q9UHB9 | 3 | 3 | 8.5 | 70.729 | 0.0000 | inf |
| ENOPH1 | Q9UHY7 | 2 | 2 | 13 | 28.932 | 0.0004 | inf |
| SLC25A13 | Q9UJS0 | 6 | 4 | 15.4 | 74.175 | 0.0000 | inf |
| APPL1 | Q9UKG1 | 3 | 3 | 6.8 | 79.663 | 0.0001 | inf |
| CLEC11A | Q9Y240 | 3 | 3 | 11.8 | 35.694 | 0.0000 | inf |
| PLAA | Q9Y263 | 3 | 3 | 5.4 | 87.156 | 0.0000 | inf |
| EPB41L3 | Q9Y2J2 | 7 | 3 | 7.7 | 120.68 | 0.0000 | inf |
| NOP58 | Q9Y2X3 | 3 | 3 | 8.9 | 59.578 | 0.0000 | inf |
| ACOT9 | Q9Y305 | 7 | 7 | 20.5 | 49.901 | 0.0000 | inf |
| TMED5 | Q9Y3A6 | 4 | 4 | 25.8 | 26.005 | 0.0000 | inf |
| REXO2 | Q9Y3B8 | 2 | 2 | 11.8 | 26.832 | 0.0000 | inf |
| GMPPB | Q9Y5P6 | 6 | 6 | 20.8 | 39.834 | 0.0001 | inf |
| SNX9 | Q9Y5X1 | 4 | 4 | 8.9 | 66.591 | 0.0035 | inf |
| NUBP2 | Q9Y5Y2 | 2 | 2 | 19.6 | 28.825 | 0.0000 | inf |
| OAS3 | Q9Y6K5 | 13 | 13 | 20.1 | 121.17 | 0.0001 | inf |
| MYOF | Q9NZM1 | 29 | 29 | 20.3 | 234.71 | 0.0000 | 83.6113 |
| PCOLCE | Q15113 | 16 | 16 | 51.2 | 47.972 | 0.0084 | 29.1537 |
| RNPEP | Q9H4A4 | 19 | 19 | 40.6 | 72.595 | 0.0032 | 23.3825 |
| FASN | P49327 | 77 | 77 | 45.7 | 273.42 | 0.0002 | 21.0368 |
| RRBP1 | Q9P2E9 | 37 | 36 | 35.7 | 152.45 | 0.0180 | 20.4485 |
| EPRS1 | P07814 | 24 | 24 | 24 | 170.59 | 0.0107 | 19.6081 |
| COPA | P53621 | 39 | 39 | 44.7 | 138.34 | 0.0012 | 16.5505 |
| ERAP1 | Q9NZ08 | 20 | 20 | 28.5 | 107.23 | 0.0256 | 13.3430 |
| OGDH | Q02218;Q9ULD0 | 20 | 20 | 28.5 | 115.93 | 0.0048 | 13.2117 |
| SEC23A | Q15436 | 20 | 17 | 35.3 | 86.16 | 0.0061 | 11.7561 |
| COL14A1 | Q05707 | 57 | 57 | 45.9 | 193.51 | 0.0035 | 11.6701 |
| COPG1 | Q9Y678;Q9UBF2 | 26 | 26 | 44.3 | 97.717 | 0.0046 | 11.0879 |
| HEXB | P07686 | 12 | 12 | 32.6 | 63.111 | 0.0456 | 10.9141 |
| COL12A1 | Q99715 | 113 | 113 | 48.2 | 333.14 | 0.0496 | 10.7271 |
| COL3A1 | P02461;CON__P04258 | 11 | 11 | 9.2 | 138.56 | 0.0003 | 10.6960 |
| CORO7 | P57737 | 13 | 13 | 22.7 | 100.6 | 0.0014 | 10.4897 |
| DPYSL3 | Q14195 | 24 | 19 | 67.4 | 61.963 | 0.0127 | 9.6481 |
| GPNMB | Q14956 | 4 | 4 | 7.7 | 63.922 | 0.0166 | 9.4276 |
| COL6A2 | P12110 | 29 | 29 | 35.4 | 108.58 | 0.0035 | 9.4236 |
| MAP4 | P27816 | 23 | 23 | 28.2 | 121 | 0.0400 | 8.7621 |
| STAT1 | P42224 | 29 | 29 | 46.7 | 87.334 | 0.0322 | 8.5333 |
| CTSZ | Q9UBR2 | 6 | 6 | 29 | 33.868 | 0.0145 | 8.4699 |
| IGHV4-34 | P06331;P0DP08;P0DP06;A0A0C4DH41;P01825;P01824;P0DP07;A0A087WSY4 | 3 | 2 | 37.4 | 13.815 | 0.0005 | 8.4592 |
| FBLN1 | P23142;CON__ENSEMBL:ENSBTAP00000016046 | 19 | 19 | 38.7 | 77.213 | 0.0078 | 8.2193 |
| VARS1 | P26640 | 19 | 19 | 25.4 | 140.47 | 0.0102 | 8.1112 |
| MZB1 | Q8WU39 | 8 | 8 | 66.1 | 20.694 | 0.0495 | 8.0426 |
| PLS3 | P13797;Q14651 | 34 | 27 | 67 | 70.81 | 0.0048 | 7.9595 |
| CORO1B | Q9BR76;A9Z1Z3 | 9 | 9 | 29 | 54.234 | 0.0006 | 7.6921 |
| COL6A1 | P12109 | 44 | 43 | 51.9 | 108.53 | 0.0041 | 7.6304 |
| COL6A3 | P12111 | 160 | 160 | 58.9 | 343.67 | 0.0026 | 7.4665 |
| CD14 | P08571 | 11 | 11 | 37.1 | 40.076 | 0.0161 | 7.3471 |
| CKB | P12277;P06732 | 21 | 21 | 71.9 | 42.644 | 0.0012 | 7.3383 |
| DYNC1H1 | Q14204 | 95 | 95 | 28.3 | 532.4 | 0.0072 | 7.2418 |
| VPS35 | Q96QK1 | 17 | 17 | 25.9 | 91.706 | 0.0340 | 6.8265 |
| NAGK | Q9UJ70 | 16 | 16 | 62.8 | 37.375 | 0.0270 | 6.6318 |
| IGHV3-43D | P0DP04;P01782 | 3 | 2 | 34.7 | 13.017 | 0.0376 | 6.5910 |
|  | P0DOX2 | 17 | 3 | 53.4 | 48.934 | 0.0001 | 6.5870 |
| SAMHD1 | Q9Y3Z3 | 32 | 32 | 59.3 | 72.2 | 0.0064 | 6.3524 |
| IGFALS | P35858 | 20 | 20 | 43 | 66.034 | 0.0251 | 6.3145 |
| PLEC | Q15149;P58107 | 191 | 190 | 47.4 | 531.78 | 0.0002 | 6.3106 |
| MAT2A | P31153 | 3 | 3 | 10.4 | 43.66 | 0.0006 | 6.2732 |
| F12 | P00748 | 10 | 10 | 29.4 | 67.791 | 0.0126 | 6.2300 |
| IGHV6-1 | A0A0B4J1U7 | 2 | 2 | 13.2 | 13.481 | 0.0008 | 6.0447 |
| RTCB | Q9Y3I0 | 10 | 10 | 28.7 | 55.21 | 0.0094 | 6.0391 |
| CD163 | Q86VB7 | 26 | 26 | 34.5 | 125.45 | 0.0036 | 6.0014 |
| TYMP | P19971 | 19 | 19 | 56.2 | 49.955 | 0.0430 | 5.9172 |
| COPB1 | P53618 | 19 | 19 | 27.1 | 107.14 | 0.0018 | 5.8673 |
| CTSD | P07339 | 20 | 20 | 60.2 | 44.552 | 0.0314 | 5.7370 |
| MYH11 | P35749 | 44 | 30 | 29.4 | 227.34 | 0.0109 | 5.6978 |
| IGLV3-25 | P01717 | 5 | 2 | 36.6 | 12.011 | 0.0072 | 5.6686 |
| LTBP1 | Q14766 | 7 | 7 | 5.8 | 186.79 | 0.0350 | 5.5756 |
| AKR1B1 | P15121;C9JRZ8 | 12 | 12 | 57.3 | 35.853 | 0.0012 | 5.4717 |
| TIMP3 | P35625 | 6 | 6 | 34.1 | 24.145 | 0.0075 | 5.4408 |
| IGHV3-49 | A0A0A0MS15 | 3 | 2 | 26.1 | 13.056 | 0.0064 | 5.3619 |
| CTSB | P07858 | 14 | 14 | 50.7 | 37.821 | 0.0256 | 5.2508 |
| TGFBI | Q15582 | 35 | 35 | 69.3 | 74.68 | 0.0081 | 5.2357 |
| MVP | Q14764 | 29 | 29 | 49.4 | 99.326 | 0.0041 | 5.2318 |
| TNC | P24821 | 67 | 67 | 41.2 | 240.85 | 0.0227 | 5.2106 |
| HK3 | P52790 | 18 | 16 | 27.8 | 99.024 | 0.0007 | 5.1403 |
| COPE | O14579 | 9 | 9 | 52.3 | 34.482 | 0.0088 | 5.1360 |
| IGHV3-72 | A0A0B4J1Y9 | 4 | 3 | 40.3 | 13.203 | 0.0002 | 5.0772 |
| ATP6V1B2 | P21281;P15313 | 22 | 22 | 60.5 | 56.5 | 0.0021 | 5.0346 |
| CLTC | Q00610;P53675 | 76 | 76 | 55.5 | 191.61 | 0.0230 | 5.0083 |
| LAP3 | P28838 | 26 | 26 | 67.4 | 56.166 | 0.0249 | 4.9625 |
| ME2 | P23368;Q16798 | 17 | 17 | 51 | 65.443 | 0.0193 | 4.8858 |
| DHX9 | Q08211 | 25 | 25 | 25 | 140.96 | 0.0192 | 4.8555 |
| COPB2 | P35606 | 24 | 23 | 37 | 102.49 | 0.0058 | 4.8208 |
| SUCLG1 | P53597 | 6 | 6 | 26.6 | 36.249 | 0.0031 | 4.6482 |
| CAPG | P40121 | 13 | 13 | 47.4 | 38.498 | 0.0040 | 4.6380 |
| TXNDC5 | Q8NBS9 | 17 | 17 | 54.2 | 47.628 | 0.0179 | 4.5503 |
| IGHG3 | P01860 | 20 | 8 | 66.6 | 41.287 | 0.0114 | 4.4460 |
| C7 | P10643;CON__Q29RQ1 | 27 | 27 | 45.2 | 93.517 | 0.0415 | 4.4083 |
| NDUFV1 | P49821 | 7 | 7 | 25.4 | 50.817 | 0.0400 | 4.3790 |
| ASAH1 | Q13510 | 12 | 12 | 35.4 | 44.659 | 0.0231 | 4.3524 |
| RARS1 | P54136 | 11 | 11 | 22.7 | 75.378 | 0.0050 | 4.3098 |
| SND1 | Q7KZF4 | 24 | 24 | 35.3 | 102 | 0.0030 | 4.2356 |
| P4HA1 | P13674 | 11 | 11 | 24.5 | 61.049 | 0.0078 | 4.2007 |
| PYGB | P11216 | 22 | 16 | 34.8 | 96.695 | 0.0115 | 4.1800 |
| NARS1 | O43776 | 10 | 10 | 24.5 | 62.942 | 0.0039 | 4.1791 |
| THY1 | P04216 | 4 | 4 | 24.8 | 17.935 | 0.0041 | 4.1322 |
| TPP1 | O14773 | 8 | 8 | 25.2 | 61.247 | 0.0381 | 4.1088 |
| ERO1A | Q96HE7 | 10 | 9 | 34.2 | 54.392 | 0.0042 | 4.0810 |
| MBOAT7 | Q96N66 | 3 | 3 | 8.7 | 52.764 | 0.0048 | 4.0622 |
| EFEMP1 | Q12805 | 15 | 15 | 44.2 | 54.64 | 0.0118 | 4.0600 |
| EIF3B | P55884 | 14 | 13 | 23.8 | 92.48 | 0.0182 | 3.9979 |
| CFH | P08603 | 59 | 50 | 61.3 | 139.09 | 0.0356 | 3.9739 |
| PGLYRP2 | Q96PD5;CON__ENSEMBL:ENSBTAP00000016285 | 18 | 18 | 50.3 | 62.216 | 0.0113 | 3.9380 |
| IGHA1 | P01876 | 20 | 12 | 72 | 37.654 | 0.0022 | 3.9103 |
| IFI16 | Q16666;Q6K0P9 | 8 | 8 | 14.6 | 88.255 | 0.0061 | 3.8712 |
| CAPN2 | P17655 | 14 | 14 | 28.3 | 79.994 | 0.0018 | 3.8434 |
| LRRC59 | Q96AG4 | 7 | 7 | 30 | 34.93 | 0.0195 | 3.8194 |
| ARCN1 | P48444 | 15 | 15 | 38.2 | 57.21 | 0.0088 | 3.8095 |
| LAMB1 | P07942 | 16 | 16 | 12.8 | 198.04 | 0.0024 | 3.7775 |
| IGKV3D-11 | A0A0A0MRZ8;P04433 | 3 | 3 | 26.1 | 12.625 | 0.0496 | 3.7629 |
| HLA-DRA | P01903;P01906 | 7 | 7 | 32.7 | 28.621 | 0.0159 | 3.7390 |
| IGHG4 | P01861 | 14 | 6 | 55.4 | 35.94 | 0.0232 | 3.7265 |
| SNX6 | Q9UNH7 | 5 | 4 | 14.3 | 46.648 | 0.0042 | 3.7118 |
| KIF5B | P33176;O60282;Q12840 | 16 | 16 | 22.6 | 109.68 | 0.0126 | 3.6178 |
| TF | P02787;CON__Q2HJF0 | 59 | 58 | 70.8 | 77.063 | 0.0014 | 3.5816 |
| DDX39B | Q13838;O00148 | 12 | 12 | 37.4 | 48.991 | 0.0033 | 3.5501 |
| AP2A2 | O94973 | 20 | 14 | 35.3 | 103.96 | 0.0034 | 3.5288 |
| AIFM1 | O95831 | 9 | 9 | 20.6 | 66.9 | 0.0151 | 3.5216 |
| DDX5 | P17844 | 17 | 12 | 36.8 | 69.147 | 0.0214 | 3.5032 |
| NID2 | Q14112 | 19 | 18 | 19.4 | 151.25 | 0.0273 | 3.4746 |
| DDX3X | O00571;O15523;Q9NQI0 | 12 | 11 | 26.6 | 73.243 | 0.0016 | 3.4740 |
| PPT1 | P50897 | 6 | 6 | 37.3 | 34.193 | 0.0047 | 3.4522 |
| DPYSL2 | Q16555 | 24 | 19 | 63.6 | 62.293 | 0.0008 | 3.4381 |
| PKM | P14618 | 39 | 38 | 75.3 | 57.936 | 0.0003 | 3.4283 |
| RCN3 | Q96D15 | 11 | 11 | 56.7 | 37.493 | 0.0102 | 3.4114 |
| SOD2 | P04179 | 9 | 9 | 77 | 24.75 | 0.0202 | 3.3961 |
| CPA3 | P15088 | 9 | 9 | 26.9 | 48.669 | 0.0414 | 3.3825 |
| IGHV3-7 | P01780;A0A0B4J1V1 | 4 | 2 | 32.5 | 12.943 | 0.0042 | 3.3823 |
| MYO1F | O00160;Q12965 | 10 | 10 | 11.7 | 124.84 | 0.0102 | 3.3360 |
| AKR1A1 | P14550 | 17 | 17 | 68.9 | 36.573 | 0.0011 | 3.3266 |
| PTPN6 | P29350 | 17 | 17 | 41.5 | 67.56 | 0.0114 | 3.2460 |
| HK1 | P19367;Q2TB90 | 19 | 17 | 23.2 | 102.48 | 0.0176 | 3.2383 |
| MANF | P55145 | 6 | 6 | 34.6 | 20.7 | 0.0220 | 3.2380 |
| CMA1 | P23946 | 9 | 9 | 52.6 | 27.324 | 0.0496 | 3.2329 |
| AARS1 | P49588 | 18 | 18 | 30.4 | 106.81 | 0.0026 | 3.2228 |
| SARS1 | P49591 | 9 | 9 | 23.9 | 58.777 | 0.0067 | 3.2153 |
| EMILIN1 | Q9Y6C2 | 26 | 26 | 37.3 | 106.69 | 0.0149 | 3.1746 |
| IQGAP1 | P46940;Q86VI3 | 70 | 67 | 55.2 | 189.25 | 0.0011 | 3.1528 |
| ATP1A1 | P05023;P13637;P50993;Q13733;P54707;P20648 | 22 | 21 | 27.6 | 112.89 | 0.0353 | 3.1372 |
| IGLV3-21 | P80748;A0A075B6K5;A0A075B6K2 | 5 | 5 | 48.7 | 12.446 | 0.0346 | 3.1363 |
| LMAN1 | P49257 | 15 | 15 | 35.7 | 57.548 | 0.0206 | 3.0945 |
| HADHA | P40939 | 25 | 25 | 47.2 | 82.999 | 0.0084 | 3.0792 |
| CORO1C | Q9ULV4;Q6QEF8 | 15 | 15 | 42.4 | 53.248 | 0.0255 | 3.0617 |
| EHD4 | Q9H223 | 15 | 12 | 41.4 | 61.174 | 0.0156 | 3.0581 |
| IGHG1 | P0DOX5;P01857 | 25 | 13 | 56.3 | 49.328 | 0.0012 | 3.0412 |
| MOGS | Q13724 | 8 | 8 | 13.9 | 91.916 | 0.0308 | 3.0409 |
| SEPTIN6 | Q14141 | 8 | 4 | 28.6 | 49.716 | 0.0208 | 3.0123 |
| FLNB | O75369 | 93 | 83 | 51.3 | 278.16 | 0.0035 | 2.9599 |
| LUM | P51884;CON__Q05443 | 12 | 12 | 37.9 | 38.429 | 0.0436 | 2.9102 |
| XRCC5 | P13010 | 22 | 22 | 41.5 | 82.704 | 0.0115 | 2.9016 |
| LONP1 | P36776 | 6 | 6 | 9.3 | 106.49 | 0.0238 | 2.8887 |
| SEPTIN9 | Q9UHD8 | 11 | 11 | 32.1 | 65.401 | 0.0001 | 2.8668 |
| HSD17B4 | P51659 | 18 | 18 | 41.7 | 79.685 | 0.0111 | 2.8573 |
| EEF2 | P13639 | 44 | 43 | 59.3 | 95.337 | 0.0013 | 2.8527 |
| GLUD1 | P00367;P49448 | 23 | 23 | 56.8 | 61.397 | 0.0014 | 2.8514 |
| AHNAK | Q09666 | 157 | 157 | 53.5 | 629.09 | 0.0235 | 2.8513 |
| AFM | P43652;CON__REFSEQ:XP_585019 | 17 | 17 | 37.1 | 69.068 | 0.0342 | 2.8311 |
| TIMP1 | P01033 | 5 | 5 | 32.4 | 23.171 | 0.0075 | 2.8194 |
| CKAP4 | Q07065 | 22 | 22 | 47.8 | 66.022 | 0.0226 | 2.8087 |
| ARL8A | Q96BM9 | 6 | 3 | 43.5 | 21.416 | 0.0035 | 2.7830 |
| ANXA4 | P09525 | 15 | 15 | 54.5 | 35.882 | 0.0009 | 2.7747 |
| HCLS1 | P14317 | 10 | 10 | 25.9 | 54.013 | 0.0480 | 2.7537 |
| ITGB2 | P05107 | 24 | 24 | 37.6 | 84.781 | 0.0276 | 2.7536 |
| DNPEP | Q9ULA0 | 9 | 9 | 29.5 | 53.41 | 0.0484 | 2.7351 |
| VAPB | O95292 | 4 | 3 | 24.3 | 27.228 | 0.0162 | 2.7086 |
| IGKC | P01834 | 10 | 3 | 86.9 | 11.765 | 0.0072 | 2.6952 |
| PPP1CB | P62140 | 12 | 5 | 54.7 | 37.186 | 0.0004 | 2.6904 |
| CNDP2 | Q96KP4 | 22 | 22 | 66.7 | 52.878 | 0.0102 | 2.6763 |
| DSTN | P60981 | 6 | 6 | 39.4 | 18.506 | 0.0011 | 2.6719 |
| PTGR1 | Q14914 | 3 | 3 | 14.9 | 35.869 | 0.0207 | 2.6628 |
| KCTD12 | Q96CX2;Q6ZWB6 | 11 | 11 | 38.5 | 35.7 | 0.0014 | 2.6526 |
| EIF3A | Q14152 | 23 | 23 | 19.1 | 166.57 | 0.0134 | 2.6518 |
| ACO2 | Q99798 | 28 | 28 | 49.6 | 85.424 | 0.0156 | 2.6458 |
| FSCN1 | Q16658 | 22 | 22 | 49.7 | 54.529 | 0.0116 | 2.6445 |
| GART | P22102 | 7 | 7 | 10.7 | 107.77 | 0.0148 | 2.6413 |
| HNRNPR | O43390 | 9 | 6 | 17.2 | 70.942 | 0.0157 | 2.6330 |
| SERPINB6 | P35237 | 12 | 11 | 46.5 | 42.621 | 0.0039 | 2.6229 |
| MYLK | Q15746 | 6 | 6 | 3.5 | 210.71 | 0.0078 | 2.5378 |
| COL4A1 | P02462;P29400 | 7 | 6 | 6.4 | 160.61 | 0.0411 | 2.5068 |
| NNMT | P40261 | 10 | 10 | 53.4 | 29.574 | 0.0263 | 2.5022 |
| NANS | Q9NR45 | 9 | 9 | 38.2 | 40.307 | 0.0036 | 2.4887 |
| CAMK2D | Q13557;Q13554;Q13555;Q9UQM7 | 9 | 9 | 25.5 | 56.369 | 0.0060 | 2.4843 |
| AP2M1 | Q96CW1 | 8 | 8 | 22.8 | 49.654 | 0.0025 | 2.4840 |
| IQGAP2 | Q13576 | 13 | 11 | 13.1 | 180.58 | 0.0168 | 2.4774 |
| GC | P02774;CON__Q3MHN5;CON__ENSEMBL:ENSBTAP00000018229 | 38 | 38 | 77.6 | 52.917 | 0.0284 | 2.4673 |
| STAT3 | P40763 | 13 | 13 | 27.4 | 88.067 | 0.0058 | 2.4654 |
| APEX1 | P27695 | 10 | 10 | 43.1 | 35.554 | 0.0244 | 2.4611 |
| IGHG2 | P01859 | 18 | 9 | 69 | 35.9 | 0.0140 | 2.4514 |
| ATP2A2 | P16615;O14983 | 19 | 16 | 25.5 | 114.76 | 0.0117 | 2.4509 |
| TARDBP | Q13148 | 6 | 6 | 23.9 | 44.739 | 0.0065 | 2.4379 |
| ATP6V1A | P38606 | 25 | 25 | 54.9 | 68.303 | 0.0013 | 2.4339 |
| EIF3L | Q9Y262 | 11 | 11 | 23.8 | 66.726 | 0.0303 | 2.4187 |
| SUCLG2 | Q96I99 | 7 | 7 | 23.6 | 46.51 | 0.0475 | 2.3920 |
| PRKAR2A | P13861 | 7 | 5 | 27.7 | 45.518 | 0.0060 | 2.3875 |
| ANKFY1 | Q9P2R3 | 7 | 7 | 7.6 | 128.4 | 0.0137 | 2.3640 |
| GNAI2 | P04899;P09471;P19087;A8MTJ3;P11488 | 18 | 12 | 59.2 | 40.45 | 0.0139 | 2.3620 |
| SDHA | P31040 | 17 | 17 | 40.5 | 72.691 | 0.0289 | 2.3305 |
| GALK1 | P51570 | 6 | 6 | 29.6 | 42.272 | 0.0175 | 2.3260 |
| CNPY2 | Q9Y2B0 | 5 | 5 | 33.5 | 20.652 | 0.0186 | 2.3196 |
| ACADM | P11310 | 6 | 6 | 20.9 | 46.588 | 0.0044 | 2.3167 |
| SEPTIN7 | Q16181;Q6ZU15 | 15 | 15 | 42.3 | 50.679 | 0.0018 | 2.3147 |
| HM13 | Q8TCT9 | 4 | 4 | 14.6 | 41.488 | 0.0408 | 2.2953 |
| DYNC1I2 | Q13409 | 8 | 8 | 19.3 | 71.456 | 0.0107 | 2.2826 |
| AP2B1 | P63010 | 22 | 12 | 34 | 104.55 | 0.0008 | 2.2801 |
| SDHB | P21912 | 4 | 4 | 16.1 | 31.629 | 0.0116 | 2.2795 |
| ACTR3 | P61158;Q9P1U1;Q9C0K3 | 23 | 23 | 77 | 47.371 | 0.0060 | 2.2763 |
| IGLC3 | P0DOY3;P0DOY2;P0CF74;A0M8Q6 | 9 | 3 | 93.4 | 11.265 | 0.0243 | 2.2759 |
| ECHDC1 | Q9NTX5 | 10 | 10 | 46.6 | 33.698 | 0.0229 | 2.2688 |
| ARPC1B | O15143 | 16 | 15 | 53 | 40.949 | 0.0034 | 2.2637 |
| STT3A | P46977 | 13 | 12 | 20.6 | 80.529 | 0.0039 | 2.2627 |
| PRKAR1A | P10644;P31321 | 11 | 11 | 37.3 | 42.981 | 0.0022 | 2.2574 |
| COL5A1 | P20908 | 11 | 11 | 9.2 | 183.56 | 0.0299 | 2.2555 |
| PPM1F | P49593 | 4 | 4 | 14.8 | 49.83 | 0.0087 | 2.2511 |
| CPNE1 | Q99829 | 7 | 7 | 21.6 | 59.058 | 0.0385 | 2.2439 |
| ACTC1 | P68032;P68133 | 29 | 1 | 79 | 42.019 | 0.0219 | 2.1796 |
| GMPPA | Q96IJ6 | 3 | 3 | 11.2 | 46.291 | 0.0323 | 2.1766 |
| NPEPPS | P55786;A6NEC2 | 16 | 16 | 25.2 | 103.28 | 0.0125 | 2.1682 |
| CORO1A | P31146 | 20 | 20 | 47.9 | 51.026 | 0.0244 | 2.1541 |
| SCAMP2 | O15127 | 2 | 2 | 9.7 | 36.648 | 0.0239 | 2.1516 |
|  | P0DOX7 | 10 | 2 | 57 | 23.379 | 0.0148 | 2.1465 |
| NDRG1 | Q92597 | 7 | 7 | 28.2 | 42.835 | 0.0476 | 2.1400 |
| C1QA | P02745 | 6 | 6 | 29 | 26.016 | 0.0342 | 2.1383 |
| PDXK | O00764 | 9 | 9 | 42.6 | 35.102 | 0.0159 | 2.1276 |
| SYNCRIP | O60506 | 12 | 9 | 26.3 | 69.602 | 0.0109 | 2.1240 |
| VIM | P08670;P17661;P41219;P07197;P14136;Q16352;P07196 | 58 | 54 | 87.1 | 53.651 | 0.0060 | 2.1188 |
| EIF4A3 | P38919 | 12 | 8 | 39.7 | 46.871 | 0.0040 | 2.1143 |
| EIF3E | P60228 | 9 | 9 | 23.4 | 52.22 | 0.0085 | 2.1142 |
| SNX1 | Q13596 | 8 | 7 | 19.7 | 59.069 | 0.0369 | 2.1079 |
| U2AF1 | Q01081;P0DN76;Q8WU68 | 4 | 4 | 23.8 | 27.872 | 0.0388 | 2.1051 |
| HNMT | P50135 | 9 | 9 | 41.8 | 33.295 | 0.0326 | 2.1023 |
| LSP1 | P33241 | 8 | 8 | 52.8 | 37.191 | 0.0264 | 2.0866 |
| PCBP2 | Q15366;P57721 | 7 | 5 | 29 | 38.58 | 0.0216 | 2.0801 |
| GNPDA1 | P46926;Q8TDQ7 | 10 | 10 | 49.5 | 32.668 | 0.0391 | 2.0747 |
| FLNA | P21333 | 120 | 109 | 63.8 | 280.74 | 0.0486 | 2.0550 |
| ACOT2 | P49753;Q86TX2 | 5 | 5 | 19.3 | 53.218 | 0.0383 | 2.0546 |
| API5 | Q9BZZ5 | 4 | 4 | 10.5 | 59.004 | 0.0130 | 2.0532 |
| GNAI3 | P08754 | 9 | 4 | 33.9 | 40.532 | 0.0116 | 2.0350 |
| COMT | P21964 | 7 | 7 | 40.2 | 30.037 | 0.0145 | 2.0344 |
| IGKV4-1 | P06312 | 4 | 4 | 29.8 | 13.38 | 0.0378 | 2.0336 |
| EEF1G | P26641 | 19 | 19 | 59.5 | 50.118 | 0.0118 | 2.0308 |
| LMNA | P02545 | 50 | 48 | 70.5 | 74.139 | 0.0092 | 2.0268 |
| COL4A2 | P08572 | 9 | 7 | 8.5 | 167.55 | 0.0481 | 2.0179 |
| EIF2S3 | P41091;Q2VIR3 | 12 | 12 | 39.2 | 51.109 | 0.0150 | 2.0119 |
| XRCC6 | P12956 | 21 | 21 | 41.4 | 69.842 | 0.0057 | 2.0057 |
| TMED7 | Q9Y3B3 | 3 | 3 | 15.6 | 25.171 | 0.0108 | 1.9979 |
| CBR1 | P16152 | 14 | 11 | 63.5 | 30.375 | 0.0293 | 1.9976 |
| PPP1CA | P62136 | 10 | 2 | 36.7 | 37.512 | 0.0140 | 1.9927 |
| ABCF1 | Q8NE71 | 6 | 6 | 9.8 | 95.925 | 0.0037 | 1.9864 |
| EPB41L2 | O43491 | 15 | 13 | 20.9 | 112.59 | 0.0192 | 1.9800 |
| FBL | P22087;A6NHQ2 | 5 | 5 | 21.8 | 33.784 | 0.0071 | 1.9661 |
| PDIA3 | P30101 | 35 | 35 | 65.1 | 56.782 | 0.0213 | 1.9652 |
| ALDH7A1 | P49419 | 7 | 7 | 17.1 | 58.486 | 0.0200 | 1.9392 |
| TUFM | P49411 | 14 | 14 | 42.3 | 49.541 | 0.0099 | 1.9367 |
| CLIC4 | Q9Y696 | 13 | 12 | 73.1 | 28.772 | 0.0031 | 1.9303 |
| TOM1 | O60784 | 5 | 5 | 20.1 | 53.818 | 0.0041 | 1.9237 |
| CAPZA2 | P47755 | 9 | 8 | 49 | 32.949 | 0.0006 | 1.9163 |
| RPL30 | P62888 | 3 | 3 | 40.9 | 12.784 | 0.0037 | 1.9064 |
| TMOD3 | Q9NYL9 | 7 | 7 | 27.6 | 39.594 | 0.0099 | 1.8999 |
| EZR | P15311 | 22 | 11 | 36.9 | 69.412 | 0.0107 | 1.8757 |
| SEPTIN11 | Q9NVA2;Q9P0V9 | 12 | 7 | 37.5 | 49.398 | 0.0006 | 1.8721 |
| PGM1 | P36871 | 19 | 19 | 48.6 | 61.448 | 0.0158 | 1.8707 |
| RPN1 | P04843 | 24 | 24 | 47.1 | 68.569 | 0.0214 | 1.8612 |
| UBA1 | P22314 | 37 | 37 | 54.3 | 117.85 | 0.0245 | 1.8587 |
| MAPK1 | P28482;P31152 | 13 | 9 | 38.9 | 41.389 | 0.0007 | 1.8557 |
| IDH1 | O75874 | 20 | 19 | 56.8 | 46.659 | 0.0482 | 1.8552 |
| GANAB | Q14697 | 37 | 37 | 49.3 | 106.87 | 0.0161 | 1.8535 |
| CDC37 | Q16543 | 8 | 8 | 27.2 | 44.468 | 0.0016 | 1.8331 |
| HNRNPL | P14866 | 18 | 18 | 46.3 | 64.132 | 0.0298 | 1.8317 |
| RPL10A | P62906 | 8 | 8 | 33.6 | 24.831 | 0.0482 | 1.8240 |
| VPS4B | O75351;Q9UN37;Q6PIW4 | 6 | 6 | 21.8 | 49.301 | 0.0423 | 1.7962 |
| DNAJB11 | Q9UBS4 | 6 | 6 | 24.9 | 40.513 | 0.0231 | 1.7933 |
| HSP90AB1 | P08238;Q58FF7 | 39 | 20 | 49.3 | 83.263 | 0.0016 | 1.7849 |
| ACO1 | P21399 | 21 | 21 | 32.4 | 98.398 | 0.0467 | 1.7517 |
| ANXA6 | P08133 | 45 | 45 | 70.3 | 75.872 | 0.0283 | 1.7422 |
| STRAP | Q9Y3F4 | 7 | 7 | 29.1 | 38.438 | 0.0497 | 1.7353 |
| RHOA | P61586 | 11 | 3 | 72 | 21.768 | 0.0022 | 1.7279 |
| CAPZB | P47756 | 13 | 13 | 56.3 | 31.35 | 0.0240 | 1.7138 |
| PRPF19 | Q9UMS4 | 4 | 3 | 14.1 | 55.18 | 0.0049 | 1.7060 |
| QARS1 | P47897 | 9 | 9 | 17.9 | 87.798 | 0.0420 | 1.6930 |
| RPS3 | P23396 | 16 | 16 | 73.3 | 26.688 | 0.0182 | 1.6878 |
| PHGDH | O43175 | 6 | 6 | 16.5 | 56.65 | 0.0132 | 1.6776 |
| DNM2 | P50570;Q05193;Q9UQ16 | 13 | 13 | 21.3 | 98.063 | 0.0047 | 1.6665 |
| ANPEP | P15144 | 30 | 30 | 37.8 | 109.54 | 0.0057 | 1.6592 |
| EEF1A1 | P68104;Q5VTE0;Q05639 | 23 | 23 | 65.2 | 50.14 | 0.0015 | 1.6517 |
| VPS26A | O75436;Q4G0F5 | 7 | 7 | 27.8 | 38.169 | 0.0132 | 1.6347 |
| SEPTIN2 | Q15019 | 11 | 11 | 41.3 | 41.487 | 0.0396 | 1.6343 |
| TMED10 | P49755 | 5 | 5 | 22.4 | 24.976 | 0.0118 | 1.6295 |
| SLC25A24 | Q6NUK1 | 15 | 15 | 35.6 | 53.354 | 0.0285 | 1.6241 |
| ANXA11 | P50995;P27216 | 12 | 12 | 24.4 | 54.389 | 0.0037 | 1.6079 |
| GSN | P06396 | 36 | 17 | 53.6 | 85.696 | 0.0115 | 1.6060 |
| CYB5R1 | Q9UHQ9 | 3 | 3 | 12.1 | 34.094 | 0.0389 | 1.5960 |
| SRPRB | Q9Y5M8 | 5 | 5 | 25.8 | 29.702 | 0.0301 | 1.5836 |
| RACK1 | P63244 | 14 | 14 | 68.8 | 35.076 | 0.0091 | 1.5822 |
| KPNB1 | Q14974 | 24 | 24 | 37.9 | 97.169 | 0.0049 | 1.5687 |
| ACADVL | P49748 | 25 | 25 | 47.9 | 70.389 | 0.0336 | 1.5512 |
| ACTR2 | P61160 | 15 | 15 | 51.5 | 44.76 | 0.0118 | 1.5463 |
| YWHAQ | P27348 | 17 | 12 | 56.7 | 27.764 | 0.0066 | 1.5373 |
| CYC1 | P08574 | 5 | 5 | 31.4 | 35.422 | 0.0102 | 1.5105 |
| SHMT2 | P34897;P34896 | 6 | 6 | 19.6 | 55.992 | 0.0308 | 1.5090 |
| WDR1 | O75083 | 25 | 25 | 59.4 | 66.193 | 0.0160 | 1.4990 |
| UQCRC1 | P31930;O75439 | 15 | 15 | 47.5 | 52.645 | 0.0445 | 1.4941 |
| FKBP4 | Q02790 | 6 | 6 | 20.5 | 51.804 | 0.0053 | 1.4907 |
| IMMT | Q16891 | 13 | 13 | 24.1 | 83.677 | 0.0124 | 1.4764 |
| CLIC1 | O00299 | 15 | 15 | 82.6 | 26.922 | 0.0063 | 1.4558 |
| ARPC2 | O15144 | 17 | 16 | 63 | 34.333 | 0.0188 | 1.4543 |
| CANX | P27824 | 25 | 25 | 43.6 | 67.567 | 0.0215 | 1.4306 |
| DARS1 | P14868 | 14 | 14 | 35.1 | 57.136 | 0.0287 | 1.4303 |
| ENO1 | P06733;P13929 | 26 | 25 | 64.1 | 47.168 | 0.0218 | 1.4281 |
| PGD | P52209 | 24 | 24 | 66.3 | 53.139 | 0.0065 | 1.4138 |
| RPL12 | P30050 | 6 | 6 | 54.5 | 17.818 | 0.0447 | 1.4061 |
| LDHA | P00338;P07864;Q6ZMR3 | 24 | 23 | 75 | 36.688 | 0.0156 | 1.4060 |
| ETF1 | P62495 | 5 | 5 | 14.2 | 49.03 | 0.0108 | 1.4031 |
| MSN | P26038 | 44 | 32 | 64.1 | 67.819 | 0.0077 | 1.3429 |
| AP2S1 | P53680 | 3 | 3 | 19 | 17.018 | 0.0038 | 1.2928 |
| MAP2K1 | Q02750 | 7 | 5 | 27.5 | 43.439 | 0.0255 | 1.2725 |
| RAB10 | P61026 | 5 | 4 | 34 | 22.541 | 0.0000 | 0.8312 |
| NME2 | P22392;O60361 | 12 | 7 | 79.6 | 17.298 | 0.0435 | 0.7499 |
| RTN4 | Q9NQC3 | 7 | 7 | 11.3 | 129.93 | 0.0117 | 0.7422 |
| CAST | P20810 | 17 | 17 | 35 | 76.572 | 0.0109 | 0.7181 |
| VDAC3 | Q9Y277 | 5 | 5 | 19.8 | 30.658 | 0.0436 | 0.6963 |
| MLEC | Q14165 | 4 | 4 | 17.1 | 32.233 | 0.0478 | 0.6899 |
| RAB11B | Q15907;P62491 | 9 | 9 | 49.5 | 24.488 | 0.0146 | 0.6891 |
| YWHAE | P62258 | 21 | 19 | 72.5 | 29.174 | 0.0097 | 0.6233 |
| PAICS | P22234 | 9 | 9 | 27.5 | 47.079 | 0.0208 | 0.5796 |
| RAB6A | P20340;Q53S08;Q14964 | 6 | 3 | 30.3 | 23.593 | 0.0096 | 0.5721 |
| PSMB3 | P49720 | 6 | 6 | 40.5 | 22.949 | 0.0243 | 0.5593 |
| TXN | P10599 | 6 | 6 | 51.4 | 11.737 | 0.0149 | 0.5569 |
| RANBP1 | P43487 | 5 | 5 | 43.8 | 23.31 | 0.0332 | 0.5460 |
| TXNL1 | O43396 | 9 | 9 | 44.3 | 32.251 | 0.0469 | 0.5320 |
| LDHB | P07195 | 20 | 19 | 61.4 | 36.638 | 0.0022 | 0.5229 |
| HSPB1 | P04792 | 16 | 16 | 82 | 22.782 | 0.0377 | 0.5068 |
| NAPRT | Q6XQN6 | 12 | 12 | 34.6 | 57.578 | 0.0037 | 0.5065 |
| NME1 | P15531 | 10 | 5 | 70.4 | 17.149 | 0.0041 | 0.4942 |
| SKP1 | P63208 | 5 | 5 | 43.6 | 18.658 | 0.0082 | 0.4865 |
| PTPA | Q15257 | 7 | 7 | 29.3 | 40.667 | 0.0191 | 0.4856 |
| GSTM2 | P28161 | 7 | 3 | 40.4 | 25.744 | 0.0021 | 0.4834 |
| BSG | P35613 | 6 | 6 | 23.1 | 42.2 | 0.0487 | 0.4752 |
| CRKL | P46109 | 2 | 2 | 10.9 | 33.777 | 0.0167 | 0.4635 |
| RAN | P62826 | 12 | 12 | 49.1 | 24.423 | 0.0256 | 0.4460 |
| MGST3 | O14880 | 6 | 6 | 59.9 | 16.516 | 0.0098 | 0.4368 |
| IBSP | P21815 | 1 | 1 | 2.8 | 35.147 | 0.0252 | 0.4330 |
| MAOA | P21397 | 11 | 10 | 31.3 | 59.681 | 0.0209 | 0.4306 |
| OXSR1 | O95747 | 8 | 8 | 21.4 | 58.022 | 0.0104 | 0.4193 |
| RALB | P11234 | 4 | 2 | 24.3 | 23.408 | 0.0065 | 0.4181 |
| PCMT1 | P22061 | 11 | 11 | 64.8 | 24.636 | 0.0096 | 0.4140 |
| PRDX6 | P30041 | 17 | 17 | 71.4 | 25.035 | 0.0259 | 0.4087 |
| TBCA | O75347 | 8 | 8 | 53.7 | 12.855 | 0.0225 | 0.4046 |
| XPO7 | Q9UIA9 | 5 | 5 | 4.8 | 123.91 | 0.0269 | 0.4031 |
| SOD1 | P00441 | 5 | 5 | 63 | 15.936 | 0.0022 | 0.3934 |
| UCHL3 | P15374 | 7 | 7 | 46.1 | 26.182 | 0.0164 | 0.3825 |
| ADD1 | P35611 | 16 | 15 | 34.1 | 80.954 | 0.0348 | 0.3519 |
| APEH | P13798 | 16 | 16 | 27.5 | 81.224 | 0.0365 | 0.3478 |
| ACP1 | P24666 | 7 | 7 | 46.2 | 18.042 | 0.0262 | 0.3472 |
| SELENBP1 | Q13228 | 25 | 25 | 66.3 | 52.39 | 0.0425 | 0.3467 |
| MGST1 | P10620 | 4 | 4 | 27.1 | 17.598 | 0.0182 | 0.3375 |
| CARHSP1 | Q9Y2V2 | 5 | 5 | 66.7 | 15.892 | 0.0355 | 0.3363 |
| UBE2V1 | Q13404 | 7 | 3 | 49 | 16.495 | 0.0038 | 0.3343 |
| LXN | Q9BS40 | 3 | 3 | 16.7 | 25.75 | 0.0003 | 0.3148 |
| GOT1 | P17174 | 11 | 11 | 43.6 | 46.247 | 0.0020 | 0.3112 |
| HPRT1 | P00492 | 9 | 9 | 57.8 | 24.579 | 0.0042 | 0.3096 |
| APOC2 | P02655 | 5 | 5 | 52.5 | 11.284 | 0.0138 | 0.3034 |
| MPST | P25325 | 8 | 8 | 40.1 | 33.178 | 0.0032 | 0.2942 |
| NQO2 | P16083 | 4 | 4 | 21.6 | 25.918 | 0.0180 | 0.2876 |
| FLOT2 | Q14254 | 12 | 12 | 32.7 | 47.064 | 0.0410 | 0.2874 |
| PSMB6 | P28072 | 3 | 3 | 13 | 25.357 | 0.0008 | 0.2600 |
| AK1 | P00568;Q9Y6K8 | 12 | 11 | 66 | 21.635 | 0.0332 | 0.2577 |
| STOM | P27105 | 14 | 14 | 60.8 | 31.73 | 0.0215 | 0.2577 |
| ADH1B | P00325;P07327;P00326;P08319 | 20 | 20 | 74.9 | 39.854 | 0.0311 | 0.2517 |
| NSFL1C | Q9UNZ2 | 8 | 8 | 30.3 | 40.572 | 0.0016 | 0.2466 |
| ITIH3 | Q06033 | 12 | 12 | 22 | 99.848 | 0.0284 | 0.2355 |

Supplementary table 4. Differential expression of proteins in FHN samples compared to sclerotic samples

| **Gene Name** | **Protein IDs** | **Peptides** | **Unique peptides** | **Sequence coverage [%]** | **Mol. weight [kDa]** | **P-value** | **FC** |
| --- | --- | --- | --- | --- | --- | --- | --- |
| IGKV2D-40 | P01614;A0A087WW87 | 3 | 1 | 28.1 | 13.31 | 0.0001 | inf |
| IGKV6D-21 | A0A0A0MT36;A0A0C4DH24;A0A0C4DH26 | 2 | 2 | 28.9 | 12.34 | 0.0000 | inf |
| IGHV2-26 | A0A0B4J1V2 | 2 | 2 | 17.6 | 13.182 | 0.0003 | inf |
| IGHV3-73 | A0A0B4J1V6 | 4 | 2 | 35.3 | 12.858 | 0.0000 | inf |
| IGHV3-38 | A0A0C4DH36 | 2 | 2 | 19 | 12.758 | 0.0000 | inf |
| IFIT3 | O14879 | 9 | 9 | 29.6 | 55.984 | 0.0004 | inf |
| PLSCR1 | O15162 | 4 | 4 | 15.7 | 35.049 | 0.0000 | inf |
| DNAJC13 | O75165 | 3 | 3 | 2 | 254.41 | 0.0003 | inf |
| PGLYRP1 | O75594 | 4 | 4 | 33.2 | 21.731 | 0.0002 | inf |
| DYSF | O75923 | 4 | 4 | 3 | 237.29 | 0.0008 | inf |
| PLPBP | O94903 | 3 | 3 | 15.3 | 30.344 | 0.0001 | inf |
| LUC7L3 | O95232 | 3 | 3 | 9 | 51.466 | 0.0000 | inf |
| FMNL1 | O95466 | 6 | 6 | 8.4 | 121.85 | 0.0000 | inf |
| OAS1 | P00973 | 8 | 8 | 28 | 46.028 | 0.0007 | inf |
| IGLV1-40 | P01703 | 3 | 2 | 39 | 12.301 | 0.0000 | inf |
| IGHV1-69 | P01742;A0A0B4J2H0 | 3 | 2 | 33.3 | 12.659 | 0.0000 | inf |
| GLA | P06280 | 4 | 4 | 13.5 | 48.766 | 0.0001 | inf |
| SERPINE2 | P07093 | 3 | 3 | 12.3 | 44.002 | 0.0002 | inf |
| PTPRC | P08575 | 4 | 4 | 4.2 | 147.48 | 0.0003 | inf |
| HCK | P08631 | 4 | 2 | 8.6 | 59.599 | 0.0000 | inf |
| IFIT1 | P09914 | 12 | 12 | 37.2 | 55.36 | 0.0001 | inf |
| ALOX5 | P09917 | 4 | 4 | 9.6 | 77.982 | 0.0014 | inf |
| SULT1A3 | P0DMM9;P0DMN0 | 9 | 4 | 46.1 | 34.196 | 0.0004 | inf |
| GYS1 | P13807 | 2 | 2 | 5.6 | 83.785 | 0.0000 | inf |
| H1-2 | P16403 | 9 | 2 | 27.2 | 21.364 | 0.0002 | inf |
| NFKB1 | P19838 | 2 | 2 | 4 | 105.35 | 0.0000 | inf |
| MX2 | P20592 | 8 | 5 | 14.7 | 82.088 | 0.0000 | inf |
| IBSP | P21815 | 1 | 1 | 2.8 | 35.147 | 0.0001 | inf |
| IVD | P26440 | 2 | 2 | 8.7 | 46.65 | 0.0034 | inf |
| HMGB2 | P26583 | 6 | 4 | 30.1 | 24.033 | 0.0000 | inf |
| PML | P29590 | 5 | 5 | 6.7 | 97.55 | 0.0018 | inf |
| CDA | P32320 | 4 | 4 | 46.6 | 16.185 | 0.0001 | inf |
| MPI | P34949 | 4 | 4 | 17.5 | 46.655 | 0.0024 | inf |
| COL15A1 | P39059 | 6 | 6 | 5.5 | 141.72 | 0.0000 | inf |
| PTGDS | P41222 | 3 | 3 | 21.1 | 21.029 | 0.0002 | inf |
| GZMK | P49863 | 2 | 2 | 12.5 | 28.882 | 0.0000 | inf |
| RPS6KA3 | P51812;Q15418 | 2 | 1 | 3.9 | 83.735 | 0.0001 | inf |
| SEC24C | P53992 | 5 | 5 | 6.8 | 118.32 | 0.0004 | inf |
| TAP2 | Q03519;Q9NUT2;Q9NRK6 | 5 | 5 | 10.8 | 75.663 | 0.0015 | inf |
| BST1 | Q10588 | 5 | 5 | 21.1 | 35.724 | 0.0000 | inf |
| G3BP1 | Q13283 | 3 | 3 | 9 | 52.164 | 0.0000 | inf |
| SQSTM1 | Q13501 | 3 | 3 | 11.6 | 47.687 | 0.0000 | inf |
| SF3A1 | Q15459 | 2 | 2 | 3.4 | 88.885 | 0.0000 | inf |
| MAPK14 | Q16539 | 5 | 5 | 21.7 | 41.293 | 0.0003 | inf |
| WASHC4 | Q2M389 | 4 | 4 | 4.7 | 136.4 | 0.0001 | inf |
| THEMIS2 | Q5TEJ8 | 3 | 3 | 8.2 | 72.048 | 0.0000 | inf |
| ECPAS | Q5VYK3 | 2 | 2 | 1.6 | 204.29 | 0.0000 | inf |
| STEAP4 | Q687X5 | 4 | 4 | 15 | 51.981 | 0.0000 | inf |
| CRLF3 | Q8IUI8 | 6 | 6 | 19.2 | 49.765 | 0.0010 | inf |
| DOCK2 | Q92608 | 5 | 5 | 4 | 211.95 | 0.0000 | inf |
| ATP6V0A1 | Q93050 | 2 | 2 | 3.1 | 96.412 | 0.0000 | inf |
| ATP2A3 | Q93084 | 12 | 9 | 17 | 109.25 | 0.0001 | inf |
| CTHRC1 | Q96CG8 | 3 | 3 | 11.5 | 26.224 | 0.0000 | inf |
| AKAP9 | Q99996 | 3 | 3 | 0.9 | 452.98 | 0.0013 | inf |
| EMILIN2 | Q9BXX0 | 9 | 9 | 11.7 | 115.69 | 0.0000 | inf |
| SEC11C | Q9BY50 | 4 | 4 | 20.3 | 21.542 | 0.0005 | inf |
| TNKS1BP1 | Q9C0C2 | 3 | 3 | 2.9 | 181.79 | 0.0003 | inf |
| DNAJC5 | Q9H3Z4 | 2 | 2 | 14.6 | 22.149 | 0.0002 | inf |
| ATP13A1 | Q9HD20 | 3 | 3 | 3.7 | 132.95 | 0.0007 | inf |
| CD209 | Q9NNX6;Q9H2X3 | 5 | 5 | 28 | 45.774 | 0.0009 | inf |
| RPRD1B | Q9NQG5 | 2 | 2 | 9.5 | 36.899 | 0.0003 | inf |
| CGGBP1 | Q9UFW8 | 1 | 1 | 6.6 | 18.82 | 0.0000 | inf |
| FASN | P49327 | 77 | 77 | 45.7 | 273.42 | 0.0138 | 19.6672 |
| MZB1 | Q8WU39 | 8 | 8 | 66.1 | 20.694 | 0.0151 | 16.1812 |
| FBN1 | P35555 | 93 | 87 | 44.2 | 312.3 | 0.0165 | 12.6374 |
| MYOF | Q9NZM1 | 29 | 29 | 20.3 | 234.71 | 0.0135 | 9.1267 |
| RNPEP | Q9H4A4 | 19 | 19 | 40.6 | 72.595 | 0.0341 | 7.8801 |
| PTPN6 | P29350 | 17 | 17 | 41.5 | 67.56 | 0.0254 | 6.9761 |
| IGHV2-5 | P01817 | 2 | 2 | 31.9 | 13.231 | 0.0007 | 6.3156 |
| ME1 | P48163 | 8 | 8 | 26.9 | 64.149 | 0.0018 | 6.0764 |
| TPSAB1 | Q15661;P20231;Q9BZJ3;P05981;A6NIE9 | 10 | 10 | 35.6 | 30.515 | 0.0272 | 5.4123 |
| CTSS | P25774 | 10 | 10 | 40.2 | 37.495 | 0.0287 | 5.1415 |
| NCF2 | P19878 | 13 | 13 | 32.5 | 59.761 | 0.0174 | 5.0538 |
| VPS35 | Q96QK1 | 17 | 17 | 25.9 | 91.706 | 0.0334 | 4.5311 |
| STAT1 | P42224 | 29 | 29 | 46.7 | 87.334 | 0.0485 | 4.2576 |
| FOLR2 | P14207;P41439 | 4 | 4 | 21.6 | 29.279 | 0.0011 | 3.7035 |
| MYO1F | O00160;Q12965 | 10 | 10 | 11.7 | 124.84 | 0.0122 | 3.6821 |
| EMILIN1 | Q9Y6C2 | 26 | 26 | 37.3 | 106.69 | 0.0020 | 3.6126 |
| GLB1 | P16278 | 6 | 6 | 13.6 | 76.074 | 0.0471 | 3.5465 |
| GRN | P28799 | 8 | 8 | 21.4 | 63.544 | 0.0393 | 3.3578 |
| PLEC | Q15149;P58107 | 191 | 190 | 47.4 | 531.78 | 0.0095 | 3.2557 |
| IGKV1-16 | P04430 | 2 | 2 | 29.1 | 12.618 | 0.0164 | 3.1881 |
| HLA-B | P01889 | 13 | 5 | 39 | 40.46 | 0.0098 | 3.1070 |
| TUBA4A | P68366 | 23 | 4 | 60 | 49.924 | 0.0100 | 3.0629 |
| ASAH1 | Q13510 | 12 | 12 | 35.4 | 44.659 | 0.0374 | 2.9545 |
| CTSA | P10619 | 8 | 8 | 20 | 54.465 | 0.0288 | 2.9219 |
| SOD2 | P04179 | 9 | 9 | 77 | 24.75 | 0.0102 | 2.9137 |
| TAPBP | O15533 | 5 | 5 | 11.4 | 47.625 | 0.0402 | 2.8764 |
|  | P0DOX2 | 17 | 3 | 53.4 | 48.934 | 0.0042 | 2.8193 |
| IFI16 | Q16666;Q6K0P9 | 8 | 8 | 14.6 | 88.255 | 0.0119 | 2.7812 |
| IGHV6-1 | A0A0B4J1U7 | 2 | 2 | 13.2 | 13.481 | 0.0183 | 2.7687 |
| MYH10 | P35580 | 46 | 29 | 30.1 | 229 | 0.0115 | 2.7273 |
| ECHDC1 | Q9NTX5 | 10 | 10 | 46.6 | 33.698 | 0.0238 | 2.7213 |
| CTSG | P08311 | 12 | 12 | 51 | 28.837 | 0.0275 | 2.7135 |
| ALDH9A1 | P49189 | 15 | 15 | 35.4 | 53.801 | 0.0288 | 2.6767 |
| CPNE3 | O75131;Q86YQ8;Q96A23;Q9UBL6;Q8IYJ1;Q96FN4;Q9HCH3;O95741 | 11 | 11 | 27.7 | 60.13 | 0.0050 | 2.6286 |
| ARL8A | Q96BM9 | 6 | 3 | 43.5 | 21.416 | 0.0222 | 2.5904 |
| RAB8B | Q92930 | 3 | 2 | 17.9 | 23.584 | 0.0164 | 2.5611 |
| MYH9 | P35579;A7E2Y1;REV__Q9UKV3 | 130 | 108 | 59 | 226.53 | 0.0286 | 2.5549 |
| GMFG | O60234 | 6 | 4 | 65.5 | 16.801 | 0.0475 | 2.5478 |
| STAT3 | P40763 | 13 | 13 | 27.4 | 88.067 | 0.0036 | 2.5355 |
| HNMT | P50135 | 9 | 9 | 41.8 | 33.295 | 0.0393 | 2.5326 |
| LTA4H | P09960 | 22 | 22 | 48.8 | 69.284 | 0.0286 | 2.5168 |
| HK3 | P52790 | 18 | 16 | 27.8 | 99.024 | 0.0103 | 2.5002 |
| NID2 | Q14112 | 19 | 18 | 19.4 | 151.25 | 0.0183 | 2.4978 |
| GM2A | P17900 | 2 | 2 | 15.5 | 20.838 | 0.0366 | 2.4938 |
| TPP1 | O14773 | 8 | 8 | 25.2 | 61.247 | 0.0066 | 2.4891 |
| AKR1C3 | P42330;P17516 | 8 | 4 | 39.6 | 36.853 | 0.0405 | 2.4620 |
| NIBAN2 | Q96TA1 | 14 | 14 | 28.8 | 84.137 | 0.0256 | 2.4164 |
| TYMP | P19971 | 19 | 19 | 56.2 | 49.955 | 0.0233 | 2.3953 |
| HSPG2 | P98160 | 59 | 59 | 21.5 | 468.83 | 0.0393 | 2.3947 |
| PRKAR1A | P10644;P31321 | 11 | 11 | 37.3 | 42.981 | 0.0269 | 2.3903 |
| AKR1B1 | P15121;C9JRZ8 | 12 | 12 | 57.3 | 35.853 | 0.0267 | 2.3847 |
| PDXK | O00764 | 9 | 9 | 42.6 | 35.102 | 0.0177 | 2.3744 |
| AKAP12 | Q02952 | 12 | 12 | 11.3 | 191.48 | 0.0404 | 2.3429 |
| TNS1 | Q9HBL0;Q63HR2 | 11 | 10 | 9.4 | 185.7 | 0.0251 | 2.3303 |
| SEC31A | O94979 | 19 | 19 | 23.1 | 133.01 | 0.0430 | 2.3288 |
| CLTC | Q00610;P53675 | 76 | 76 | 55.5 | 191.61 | 0.0450 | 2.3255 |
| PPM1F | P49593 | 4 | 4 | 14.8 | 49.83 | 0.0065 | 2.3157 |
| GALK1 | P51570 | 6 | 6 | 29.6 | 42.272 | 0.0030 | 2.2598 |
| RHOG | P84095 | 8 | 7 | 57.1 | 21.308 | 0.0101 | 2.2077 |
| ANXA6 | P08133 | 45 | 45 | 70.3 | 75.872 | 0.0238 | 2.1941 |
| ESYT1 | Q9BSJ8 | 21 | 21 | 28.4 | 122.85 | 0.0292 | 2.1876 |
| PSAP | P07602 | 11 | 11 | 21 | 58.112 | 0.0074 | 2.1786 |
| IGHA1 | P01876 | 20 | 12 | 72 | 37.654 | 0.0166 | 2.1724 |
| CTSD | P07339 | 20 | 20 | 60.2 | 44.552 | 0.0448 | 2.1449 |
| NANS | Q9NR45 | 9 | 9 | 38.2 | 40.307 | 0.0228 | 2.1317 |
| NDRG1 | Q92597 | 7 | 7 | 28.2 | 42.835 | 0.0005 | 2.1228 |
| TGM2 | P21980;P49221 | 29 | 29 | 53.4 | 77.328 | 0.0100 | 2.1168 |
| PYCARD | Q9ULZ3 | 6 | 6 | 36.9 | 21.627 | 0.0021 | 2.1151 |
| GARS1 | P41250 | 14 | 14 | 27.5 | 83.165 | 0.0273 | 2.1094 |
| FLNA | P21333 | 120 | 109 | 63.8 | 280.74 | 0.0392 | 2.0681 |
| CTSZ | Q9UBR2 | 6 | 6 | 29 | 33.868 | 0.0059 | 2.0449 |
| HSPA1A | P0DMV8;P0DMV9 | 26 | 13 | 47.1 | 70.051 | 0.0001 | 2.0146 |
| GNAI2 | P04899;P09471;P19087;A8MTJ3;P11488 | 18 | 12 | 59.2 | 40.45 | 0.0061 | 2.0097 |
| ERLIN2 | O94905 | 9 | 7 | 35.1 | 37.839 | 0.0323 | 2.0026 |
| FBL | P22087;A6NHQ2 | 5 | 5 | 21.8 | 33.784 | 0.0252 | 1.9924 |
| PPP1CB | P62140 | 12 | 5 | 54.7 | 37.186 | 0.0064 | 1.9622 |
| HNRNPC | P07910;O60812;B7ZW38;B2RXH8;P0DMR1 | 8 | 8 | 23.2 | 33.67 | 0.0091 | 1.9620 |
| CD14 | P08571 | 11 | 11 | 37.1 | 40.076 | 0.0004 | 1.9545 |
| OSTF1 | Q92882 | 5 | 5 | 29.9 | 23.787 | 0.0342 | 1.9437 |
| PPT1 | P50897 | 6 | 6 | 37.3 | 34.193 | 0.0037 | 1.9376 |
| ARPC1B | O15143 | 16 | 15 | 53 | 40.949 | 0.0416 | 1.9106 |
| GMPPA | Q96IJ6 | 3 | 3 | 11.2 | 46.291 | 0.0490 | 1.9078 |
| NOP58 | Q9Y2X3 | 3 | 3 | 8.9 | 59.578 | 0.0060 | 1.9027 |
| VWA1 | Q6PCB0 | 7 | 7 | 31.9 | 46.804 | 0.0011 | 1.8906 |
| PPP2CA | P67775;P62714;P60510 | 10 | 10 | 43.4 | 35.594 | 0.0070 | 1.8872 |
| ANXA11 | P50995;P27216 | 12 | 12 | 24.4 | 54.389 | 0.0165 | 1.8830 |
| PPP1CA | P62136 | 10 | 2 | 36.7 | 37.512 | 0.0071 | 1.8807 |
| SPTAN1 | Q13813 | 61 | 61 | 33.7 | 284.54 | 0.0344 | 1.8674 |
| IGLV1-47 | P01700 | 4 | 2 | 49.6 | 12.283 | 0.0402 | 1.8374 |
| CNPY2 | Q9Y2B0 | 5 | 5 | 33.5 | 20.652 | 0.0391 | 1.7889 |
| DNAJB11 | Q9UBS4 | 6 | 6 | 24.9 | 40.513 | 0.0154 | 1.7700 |
| RHOA | P61586 | 11 | 3 | 72 | 21.768 | 0.0059 | 1.7690 |
| RAB21 | Q9UL25 | 3 | 3 | 16.4 | 24.347 | 0.0123 | 1.7586 |
| PRKACA | P17612 | 7 | 3 | 18.8 | 40.589 | 0.0167 | 1.7348 |
| ACTA2 | P62736;P63267 | 28 | 1 | 79 | 42.009 | 0.0357 | 1.7286 |
| SERPINB1 | P30740;O75830 | 23 | 22 | 60.2 | 42.741 | 0.0070 | 1.7273 |
| IGHV3-72 | A0A0B4J1Y9 | 4 | 3 | 40.3 | 13.203 | 0.0108 | 1.7273 |
| B2M | P61769 | 4 | 4 | 37.8 | 13.714 | 0.0254 | 1.7026 |
| TBCB | Q99426 | 3 | 3 | 21.3 | 27.325 | 0.0405 | 1.6819 |
| UBA1 | P22314 | 37 | 37 | 54.3 | 117.85 | 0.0248 | 1.6566 |
| LMF2 | Q9BU23 | 3 | 3 | 7.2 | 79.697 | 0.0337 | 1.6562 |
| CPNE1 | Q99829 | 7 | 7 | 21.6 | 59.058 | 0.0120 | 1.6471 |
| PKM | P14618 | 39 | 38 | 75.3 | 57.936 | 0.0206 | 1.6406 |
| GNB2 | P62879 | 13 | 4 | 50.6 | 37.331 | 0.0198 | 1.6214 |
| PGD | P52209 | 24 | 24 | 66.3 | 53.139 | 0.0339 | 1.6188 |
| CBR1 | P16152 | 14 | 11 | 63.5 | 30.375 | 0.0170 | 1.6182 |
| CAPZB | P47756 | 13 | 13 | 56.3 | 31.35 | 0.0162 | 1.6131 |
| MAPK1 | P28482;P31152 | 13 | 9 | 38.9 | 41.389 | 0.0228 | 1.6086 |
| PPP3CA | Q08209;P16298;P48454 | 3 | 3 | 8.6 | 58.687 | 0.0228 | 1.5987 |
| ACLY | P53396 | 29 | 29 | 34.8 | 120.84 | 0.0303 | 1.5956 |
| ARHGDIB | P52566 | 10 | 10 | 59.2 | 22.988 | 0.0338 | 1.5892 |
| PRKAR2A | P13861 | 7 | 5 | 27.7 | 45.518 | 0.0362 | 1.5687 |
| ANXA4 | P09525 | 15 | 15 | 54.5 | 35.882 | 0.0148 | 1.5312 |
| LMNB2 | Q03252 | 18 | 15 | 32.4 | 69.948 | 0.0127 | 1.5222 |
| PFKL | P17858 | 15 | 12 | 27.9 | 85.018 | 0.0406 | 1.5144 |
| FCGRT | P55899 | 4 | 4 | 12.9 | 39.743 | 0.0174 | 1.5129 |
| VPS4B | O75351;Q9UN37;Q6PIW4 | 6 | 6 | 21.8 | 49.301 | 0.0311 | 1.5005 |
| GPX1 | P07203 | 14 | 14 | 84.7 | 22.088 | 0.0071 | 1.4961 |
| ATG3 | Q9NT62 | 5 | 5 | 22 | 35.864 | 0.0220 | 1.4707 |
| GNA11 | P29992 | 7 | 4 | 30.1 | 42.123 | 0.0370 | 1.4595 |
| ACTG1 | P63261 | 36 | 1 | 94.7 | 41.792 | 0.0162 | 1.4430 |
| ATP2B4 | P23634;P20020;Q16720;Q01814 | 8 | 8 | 7.8 | 137.92 | 0.0044 | 1.4425 |
| HDGF | P51858;O75475;Q9Y3E1 | 8 | 7 | 50.4 | 26.788 | 0.0259 | 1.4079 |
| OTUB1 | Q96FW1 | 9 | 9 | 47.6 | 31.284 | 0.0417 | 1.4033 |
| VPS29 | Q9UBQ0 | 6 | 6 | 44.5 | 20.505 | 0.0212 | 1.4000 |
| CDC42 | P60953 | 7 | 6 | 51.8 | 21.258 | 0.0273 | 1.3983 |
| PAFAH1B1 | P43034 | 8 | 8 | 25.4 | 46.637 | 0.0033 | 1.3911 |
| TMED7 | Q9Y3B3 | 3 | 3 | 15.6 | 25.171 | 0.0185 | 1.3632 |
| RNH1 | P13489 | 20 | 20 | 71.1 | 49.973 | 0.0200 | 1.2936 |
| YWHAH | Q04917 | 13 | 10 | 54.5 | 28.218 | 0.0367 | 1.2893 |
| NME2 | P22392;O60361 | 12 | 7 | 79.6 | 17.298 | 0.0397 | 0.8153 |
| TXN | P10599 | 6 | 6 | 51.4 | 11.737 | 0.0264 | 0.7577 |
| NME1 | P15531 | 10 | 5 | 70.4 | 17.149 | 0.0233 | 0.7384 |
| PDCD6 | O75340 | 4 | 4 | 22 | 21.868 | 0.0174 | 0.6933 |
| UBE2N | P61088;Q5JXB2 | 8 | 8 | 53.3 | 17.138 | 0.0266 | 0.6649 |
| TBCA | O75347 | 8 | 8 | 53.7 | 12.855 | 0.0140 | 0.6067 |
| A2M | P01023 | 75 | 64 | 66.1 | 163.29 | 0.0218 | 0.5568 |
| SOD1 | P00441 | 5 | 5 | 63 | 15.936 | 0.0457 | 0.5525 |
| SKP1 | P63208 | 5 | 5 | 43.6 | 18.658 | 0.0400 | 0.5203 |
| CST3 | P01034 | 5 | 5 | 44.5 | 15.799 | 0.0045 | 0.5136 |
| NQO2 | P16083 | 4 | 4 | 21.6 | 25.918 | 0.0426 | 0.4785 |
| SRI | P30626 | 7 | 7 | 40.9 | 21.676 | 0.0112 | 0.4529 |
| UCHL3 | P15374 | 7 | 7 | 46.1 | 26.182 | 0.0056 | 0.4498 |
| MPST | P25325 | 8 | 8 | 40.1 | 33.178 | 0.0291 | 0.4491 |
| GOT1 | P17174 | 11 | 11 | 43.6 | 46.247 | 0.0152 | 0.4098 |
| PSMB6 | P28072 | 3 | 3 | 13 | 25.357 | 0.0182 | 0.4029 |
| NSFL1C | Q9UNZ2 | 8 | 8 | 30.3 | 40.572 | 0.0141 | 0.4019 |
| ADIPOQ | Q15848 | 2 | 2 | 15.6 | 26.413 | 0.0057 | 0.3982 |
| UROD | P06132 | 9 | 9 | 51 | 40.786 | 0.0404 | 0.3936 |
| LXN | Q9BS40 | 3 | 3 | 16.7 | 25.75 | 0.0342 | 0.3839 |
| CRTAP | O75718 | 7 | 7 | 22.2 | 46.561 | 0.0113 | 0.3802 |
| P4HA2 | O15460 | 9 | 9 | 27.5 | 60.901 | 0.0499 | 0.3563 |
| GFUS | Q13630 | 7 | 7 | 29.6 | 35.892 | 0.0285 | 0.3502 |
| PSMD9 | O00233 | 8 | 8 | 36.8 | 24.682 | 0.0295 | 0.3128 |
| GCLC | P48506 | 12 | 12 | 22 | 72.765 | 0.0246 | 0.2990 |
| HMOX1 | P09601 | 13 | 13 | 51.7 | 32.818 | 0.0168 | 0.2704 |
| PSMB5 | P28074 | 8 | 8 | 36.1 | 28.48 | 0.0061 | 0.2556 |
| APOC3 | P02656 | 6 | 6 | 55.6 | 10.852 | 0.0404 | 0.2434 |
| COL2A1 | P02458 | 7 | 5 | 5.3 | 141.78 | 0.0136 | 0.2362 |
| AIFM1 | O95831 | 9 | 9 | 20.6 | 66.9 | 0.0143 | 0.2246 |
| PSMF1 | Q92530 | 4 | 4 | 16.6 | 29.816 | 0.0186 | 0.2025 |
| CA2 | P00918 | 21 | 21 | 84.6 | 29.246 | 0.0499 | 0.1409 |
| HMBS | P08397 | 10 | 10 | 33.5 | 39.33 | 0.0146 | 0.1213 |
| CA1 | P00915 | 24 | 24 | 95 | 28.87 | 0.0481 | 0.1091 |
| RAD23A | P54725 | 9 | 8 | 52.9 | 39.609 | 0.0174 | 0.0773 |
| TNXB | P22105;Q16473 | 18 | 18 | 11.8 | 458.38 | 0.0042 | 0.0727 |
| IGLV9-49 | A0A0B4J1Y8 | 2 | 2 | 16.3 | 13.024 | 0.0001 | 0.0000 |
| IGF2 | P01344 | 2 | 2 | 13.9 | 20.14 | 0.0005 | 0.0000 |
| PPBP | P02775 | 4 | 4 | 27.3 | 13.894 | 0.0033 | 0.0000 |
| SERPINA5 | P05154 | 2 | 2 | 5.7 | 45.674 | 0.0027 | 0.0000 |
| CA3 | P07451 | 9 | 9 | 53.8 | 29.557 | 0.0001 | 0.0000 |
| PLA2G2A | P14555 | 3 | 3 | 25 | 16.083 | 0.0000 | 0.0000 |
| PZP | P20742 | 21 | 13 | 20.8 | 163.86 | 0.0041 | 0.0000 |
| PROZ | P22891 | 3 | 3 | 7.2 | 44.743 | 0.0001 | 0.0000 |
| TMOD1 | P28289;Q9NZQ9 | 15 | 15 | 54.9 | 40.569 | 0.0001 | 0.0000 |
| PKLR | P30613 | 10 | 9 | 26.1 | 61.829 | 0.0002 | 0.0000 |
| GSTT1 | P30711 | 3 | 3 | 15 | 27.335 | 0.0003 | 0.0000 |
| CDH13 | P55290 | 2 | 2 | 3.6 | 78.286 | 0.0018 | 0.0000 |
| SNRPD3 | P62318 | 2 | 2 | 15.1 | 13.916 | 0.0001 | 0.0000 |
| HLA-DRB3 | P79483 | 5 | 2 | 24.1 | 29.962 | 0.0000 | 0.0000 |
| CD276 | Q5ZPR3 | 2 | 2 | 6.7 | 57.235 | 0.0000 | 0.0000 |
| COPS5 | Q92905 | 4 | 4 | 18.9 | 37.578 | 0.0004 | 0.0000 |
| COPS8 | Q99627 | 2 | 2 | 13.9 | 23.225 | 0.0000 | 0.0000 |
| FN3K | Q9H479 | 7 | 7 | 30.1 | 35.171 | 0.0002 | 0.0000 |
| PARVB | Q9HBI1 | 7 | 6 | 23.1 | 41.714 | 0.0000 | 0.0000 |

Supplementary table 5. Differential metabolites in the sclerosis and FNF groups.

| **Metabolites** | **Compound ID** | **Sub Class** | **level** | **kegg** | **Score** | **P-value** | **FC** |
| --- | --- | --- | --- | --- | --- | --- | --- |
| SM(d16:1/16:0) | LMSP03010035 | Phosphosphingolipids |  |  | 36.1 | 0.0253 | 8.8350 |
| Pentasine | HMDB0029803 | Pentacarboxylic acids and derivatives | * |  | 36.9 | 0.0200 | 3.9868 |
| LysoPC(20:3(5Z,8Z,11Z)) | 61703 | Glycerophosphocholines | * |  | 37.9 | 0.0219 | 3.8409 |
| LysoPC(20:1(11Z)) | HMDB0010391 | Glycerophosphocholines | ** | C04230 | 37.4 | 0.0349 | 3.6577 |
| GlcCer(d18:1/16:0) | LMSP0501AA03 | Glycosphingolipids | ** | C01190 | 36 | 0.0480 | 3.4565 |
| PC(16:1(9Z)/0:0) | 40288 | Glycerophosphocholines | ** | C04230 | 38.6 | 0.0214 | 3.4009 |
| Allodesmosine | 95178 | Pentacarboxylic acids and derivatives |  |  | 37.4 | 0.0356 | 3.3827 |
| PC(0:0/18:0) | LMGP01050076 | Glycerophosphocholines | * |  | 56.9 | 0.0404 | 3.2583 |
| LysoPC(18:0) | HMDB0010384 | Glycerophosphocholines | ** | C04230 | 58.5 | 0.0376 | 3.0117 |
| 1-(2-methoxy-eicosanyl)-sn-glycero-3-phosphoethanolamine | LMGP02060023 | Glycerophosphoethanolamines |  |  | 37.9 | 0.0091 | 2.9129 |
| PC(20:1(11Z)/20:1(11Z)) | 59754 | Glycerophosphocholines | ** | C00157 | 36.2 | 0.0151 | 2.6992 |
| PC(18:1(11Z)/0:0) | LMGP01050138 | Glycerophosphocholines | ** | C04230 | 58 | 0.0114 | 2.6235 |
| PC(18:1(9Z)/0:0) | LMGP01050032 | Glycerophosphocholines | * |  | 56.7 | 0.0213 | 2.4915 |
| PC(0:0/20:4(5Z,8Z,11Z,14Z)) | LMGP01050121 | Glycerophosphocholines | * |  | 51.6 | 0.0398 | 2.4713 |
| (4E,8E,10E-d18:3)sphingosine | LMSP01080013 | Sphingoid bases |  |  | 48 | 0.0436 | 2.2129 |
| 4E,14Z-Sphingadiene | LMSP01080002 | Sphingoid bases |  |  | 46.7 | 0.0321 | 2.0872 |
| 2-(5,8-Tetradecadienyl)cyclobutanone | HMDB0037519 | Carbonyl compounds |  |  | 51.1 | 0.0413 | 1.9838 |
| LysoPC(18:2(9Z,12Z)) | HMDB0010386 | Glycerophosphocholines | *** |  | 57.7 | 0.0297 | 1.9593 |
| PC(18:2(2E,4E)/0:0) | LMGP01050034 | Glycerophosphocholines |  |  | 56.4 | 0.0488 | 1.8961 |
| 13Z-Docosenamide | 64926 | Unclassified |  |  | 38 | 0.0008 | 1.7350 |
| Hydralazine | 3163 | Benzodiazines |  | C07040 | 42.4 | 0.0115 | 1.6414 |
| 2-Tetradecylcyclobutanone | HMDB0037517 | Carbonyl compounds |  |  | 42.1 | 0.0381 | 1.6033 |
| Palmitic amide | HMDB0012273 | Fatty amides | *** |  | 47.3 | 0.0351 | 1.5963 |
| Palmitoleoyl-EA | 46564 | Amines | * |  | 38.5 | 0.0148 | 1.5794 |
| Linoleyl hydroxamic acid | LMFA08020211 | Fatty amides |  |  | 38.9 | 0.0103 | 1.5422 |
| (±)-2-(2-Furanyl)pyrrolidine | HMDB0040059 | Amines |  |  | 51.2 | 0.0410 | 1.5098 |
| 5-(4-Chloro-3-hydroxy-1-butynyl)-2,2'-bithiophene | HMDB0033269 | Unclassified |  |  | 37.3 | 0.0451 | 1.2421 |
| Vinaginsenoside R1 | 90087 | Terpene glycosides | * |  | 36 | 0.0133 | 1.1034 |
| PE(18:0/19:1(9Z)) | LMGP02010631 | Glycerophosphoethanolamines |  |  | 55.9 | 0.0489 | 0.7671 |
| 3-Oxoglutaric acid | HMDB0013701 | Short-chain keto acids and derivatives | *** |  | 38.1 | 0.0027 | 0.6294 |
| PC(15:0/20:2(11Z,14Z)) | HMDB0007946 | Glycerophosphocholines | * |  | 36.1 | 0.0258 | 0.5610 |
| Cohibin C | 90691 | Fatty alcohols | * |  | 36.6 | 0.0069 | 0.5212 |
| Cohibin D | HMDB0035398 | Fatty alcohols | * |  | 36.2 | 0.0132 | 0.5054 |
| 9-Octadecenoic acid, 18-fluoro-, (Z)-; Oleic acid, 18-fluoro- | 74844 | Unclassified |  |  | 38.7 | 0.0334 | 0.3884 |
| 2R-aminohexadecanoic acid | LMFA01100016 | Fatty Acids and Conjugates |  |  | 44 | 0.0494 | 0.3623 |
| C16 Sphinganine | LMSP01040001 | Sphingoid bases |  |  | 44.2 | 0.0451 | 0.2848 |
| LysoPE(20:4(5Z,8Z,11Z,14Z)/0:0) | HMDB0011517 | Glycerophosphoethanolamines | * |  | 47.5 | 0.0067 | 0.2552 |
| Lenticin | HMDB0061115 | Amino acids, peptides, and analogues |  | C09213 | 49.1 | 0.0487 | 0.1764 |
| PC(15:0/18:1(9Z)) | HMDB0007939 | Glycerophosphocholines | ** | C00157 | 36.9 | 0.0231 | 0.1636 |
| Cer(d18:1/24:0) | LMSP02010012 | Ceramides | ** | C00195 | 36.5 | 0.0437 | 0.1565 |
| Cer(d18:0/24:1(15Z)) | 41569 | Ceramides | * |  | 36.2 | 0.0438 | 0.1460 |
| PE(16:1(9Z)/22:4(7Z,10Z,13Z,16Z)) | LMGP02010538 | Glycerophosphoethanolamines | ** | C00350 | 37.2 | 0.0431 | 0.0542 |
| PC(18:4(6Z,9Z,12Z,15Z)/15:0) | HMDB0008231 | Glycerophosphocholines | ** | C00157 | 36.2 | 0.0139 | 0.0069 |
| PC(22:5(4Z,7Z,10Z,13Z,16Z)/16:1(9Z)) | HMDB0008660 | Glycerophosphocholines | ** | C00157 | 36.9 | 0.0010 | 0.0043 |

Supplementary table 6. Differential metabolites in the FHN and FNF groups.

| **Metabolites** | **Compound ID** | **Sub Class** | **level** | **kegg** | **Score** | **P-value** | **FC** |
| --- | --- | --- | --- | --- | --- | --- | --- |
| Squamotacin | HMDB0030444 | Fatty alcohols | * |  | 36.8 | 0.0171 | 4100151197.4445 |
| TG(8:0/i-12:0/12:0) | HMDB0071313 | Triradylcglycerols | * |  | 37.5 | 0.0000 | 1112.6194 |
| 4-Deoxyannoreticuin | 89042 | Fatty alcohols | * |  | 37.3 | 0.0035 | 252.8514 |
| Asiminenin A | HMDB0029959 | Fatty alcohols | * |  | 37.5 | 0.0014 | 47.9532 |
| Squamocin L | 87303 | Fatty alcohols | * |  | 37.5 | 0.0328 | 30.1352 |
| PA(14:1(9Z)/22:2(13Z,16Z)) | 81300 | Glycerophosphates | * |  | 36.4 | 0.0271 | 23.0857 |
| Annonsilin A | HMDB0030453 | Fatty alcohols | * |  | 36.2 | 0.0228 | 14.7828 |
| SM(d16:1/16:0) | LMSP03010035 | Phosphosphingolipids |  |  | 36.1 | 0.0005 | 14.0800 |
| Annomutacin | 87917 | Fatty alcohols | * |  | 36 | 0.0287 | 13.7792 |
| Uvaricin | 67075 | Unclassified |  |  | 37.9 | 0.0411 | 11.9035 |
| 24-Hydroxycholesterol | HMDB0001419 | Bile acids, alcohols and derivatives | *** | C13550 | 38.2 | 0.0315 | 11.6595 |
| Fludrocortisone acetate | LMST02030122 | Hydroxysteroids | ** | C00762 | 36.6 | 0.0157 | 10.9651 |
| ENDECAPHYLLIN X | 44079 | Unclassified |  |  | 37.1 | 0.0047 | 7.7732 |
| TG(8:0/8:0/8:0) | 62029 | Triradylcglycerols | * | C13044 | 38.4 | 0.0050 | 6.6200 |
| Muricatenol | HMDB0035900 | Fatty alcohols | * |  | 45.8 | 0.0099 | 6.2693 |
| Inosine | 84 | Unclassified | ** | C00294 | 58.8 | 0.0165 | 4.8615 |
| allopurinol | 865 | Pyrazolo[3,4-d]pyrimidines |  |  | 41 | 0.0188 | 4.7707 |
| 7,8,4'-Trihydroxy-3',5'-dimethoxyflavanone 4'-O-glucoside | LMPK12140098 | Unclassified |  |  | 36.6 | 0.0310 | 4.2945 |
| 1,2-Glyceryl dinitrate glucuronide | 1583 | Unclassified |  |  | 39 | 0.0240 | 4.2707 |
| 1-Kestose | 44694 | Carbohydrates and carbohydrate conjugates | * | C03661 | 55.8 | 0.0368 | 4.2643 |
| 3-hydroxy-2-(3-methoxyphenyl)-5-sulfino-3,4-dihydro-2H-1-benzopyran-7-olate | HMDB0127797 | O-methylated flavonoids | * |  | 36.4 | 0.0242 | 4.2128 |
| Arabinosylhypoxanthine | HMDB0003040 | Unclassified | * |  | 54.6 | 0.0242 | 4.1752 |
| PG(O-16:0/20:5(5Z,8Z,11Z,14Z,17Z)) | 79885 | Glycerophosphoglycerols |  |  | 38.7 | 0.0087 | 4.1507 |
| 1-(2-methoxy-eicosanyl)-sn-glycero-3-phosphoethanolamine | LMGP02060023 | Glycerophosphoethanolamines |  |  | 37.9 | 0.0031 | 3.9934 |
| GlcCer(d18:1/16:0) | LMSP0501AA03 | Glycosphingolipids | ** | C01190 | 36 | 0.0245 | 3.9194 |
| 2-Oxosuccinamate | HMDB0060350 | Short-chain keto acids and derivatives | ** | C02362 | 37.9 | 0.0078 | 3.5829 |
| PC(O-16:0/0:0) | LMGP01060010 | Glycerophosphocholines |  |  | 56.6 | 0.0203 | 3.4260 |
| 25-hydroxycholesterol | HMDB0006247 | Cholestane steroids | *** | C15519 | 37.7 | 0.0047 | 3.4176 |
| Allodesmosine | 95178 | Pentacarboxylic acids and derivatives |  |  | 37.4 | 0.0007 | 3.2330 |
| PC(0:0/20:4(5Z,8Z,11Z,14Z)) | LMGP01050121 | Glycerophosphocholines | * |  | 51.6 | 0.0458 | 3.0550 |
| PS(O-18:0/18:1(9Z)) | LMGP03020027 | Glycerophosphoserines |  |  | 36.2 | 0.0100 | 2.9682 |
| Lyso-PAF C-16 | 24070 | Unclassified |  |  | 44.7 | 0.0223 | 2.9649 |
| PC(16:1(9Z)/0:0) | 40288 | Glycerophosphocholines | ** | C04230 | 38.6 | 0.0047 | 2.7888 |
| PC(0:0/18:0) | LMGP01050076 | Glycerophosphocholines | * |  | 56.9 | 0.0003 | 2.7460 |
| PC(P-18:0/0:0) | 40405 | Glycerophosphocholines | ** | C04230 | 38.3 | 0.0337 | 2.7098 |
| LysoPC(18:0) | HMDB0010384 | Glycerophosphocholines | ** | C04230 | 58.5 | 0.0004 | 2.6524 |
| LysoPC(0:0/16:0) | HMDB0240262 | Glycerophosphocholines | *** |  | 58.6 | 0.0006 | 2.5968 |
| OOB-PE | LMGP20020034 | Oxidized glycerophospholipids |  |  | 41.7 | 0.0017 | 2.5540 |
| PC(16:0/0:0) | LMGP01050018 | Glycerophosphocholines | ** | C04230 | 57.6 | 0.0021 | 2.3373 |
| PC(18:1(11Z)/0:0) | LMGP01050138 | Glycerophosphocholines | ** | C04230 | 58 | 0.0082 | 2.1357 |
| PC(18:1(9Z)/0:0) | LMGP01050032 | Glycerophosphocholines | * |  | 56.7 | 0.0151 | 2.0236 |
| LysoPC(18:2(9Z,12Z)) | HMDB0010386 | Glycerophosphocholines | *** |  | 57.7 | 0.0457 | 1.8478 |
| Cotinine | HMDB0001046 | Pyrrolidinylpyridines | * |  | 45.8 | 0.0119 | 1.6923 |
| D-Citronellol | HMDB0035093 | Monoterpenoids | * | C09849 | 45.9 | 0.0228 | 1.6900 |
| Hexamethylphosphoramide | 72970 | Unclassified |  |  | 38.7 | 0.0091 | 1.6895 |
| (1R,2R,4R,8R)-p-Menthane-2,8,9-triol | HMDB0033574 | Monoterpenoids | * |  | 48 | 0.0301 | 1.6359 |
| Cytosine | HMDB0000630 | Pyrimidines and pyrimidine derivatives | *** | C00380 | 44.6 | 0.0360 | 1.6356 |
| 6R,7S-Epoxy-3Z,9Z-eicosadiene | LMFA12000311 | Oxygenated hydrocarbons |  |  | 53.9 | 0.0098 | 1.6162 |
| 4-Acetylimidazo[4,5-c]pyridine | HMDB0034888 | Imidazo-[4,5-c]pyridines |  |  | 43.2 | 0.0378 | 1.5882 |
| Histidinal | HMDB0012234 | Amines | ** | C01929 | 47.4 | 0.0286 | 1.5667 |
| DL-Histidinol | 63491 | Unclassified |  |  | 39.6 | 0.0097 | 1.4374 |
| Azoprocarbazine | 2106 | Unclassified |  |  | 38.7 | 0.0102 | 0.8888 |
| N-stearoyl valine | 75504 | Fatty amides |  |  | 38.8 | 0.0132 | 0.8746 |
| Bismuth subsalicylate | 66756 | Unclassified |  | C07870 | 38.2 | 0.0063 | 0.8235 |
| Flupyrsulfuron-methyl sodium | 72617 | Unclassified |  |  | 36.1 | 0.0232 | 0.8199 |
| 4-Amino-5-hydroxymethyl-2-methylpyrimidine | 3265 | Unclassified |  |  | 38.2 | 0.0343 | 0.7795 |
| 6-Phosphogluconic acid | HMDB0001316 | Carbohydrates and carbohydrate conjugates | *** | C00345 | 37 | 0.0184 | 0.7756 |
| PQQH2 | 63124 | Unclassified |  |  | 36.2 | 0.0139 | 0.7643 |
| 1-Hexadecyl-2-arachidonoyl-glycerol | 43450 | Unclassified |  |  | 37.3 | 0.0445 | 0.7004 |
| SM(d18:2/24:0) | 83781 | Phosphosphingolipids |  |  | 36.8 | 0.0102 | 0.6814 |
| PC(20:0/18:2(9Z,12Z)) | 59716 | Glycerophosphocholines | ** | C00157 | 36.6 | 0.0007 | 0.6422 |
| PC(16:0/P-18:0) | HMDB0007995 | Glycerophosphocholines | * |  | 37.7 | 0.0345 | 0.5993 |
| PC(18:0/P-16:0) | 59507 | Glycerophosphocholines | * |  | 37.2 | 0.0198 | 0.5712 |
| 3-Methylpyrrolo[1,2-a]pyrazine | HMDB0033172 | Unclassified |  |  | 43.5 | 0.0223 | 0.5651 |
| PC(15:0/20:2(11Z,14Z)) | HMDB0007946 | Glycerophosphocholines | * |  | 36.1 | 0.0341 | 0.5620 |
| 2-Ethylpyrazine | 87969 | Pyrazines | * |  | 39.4 | 0.0284 | 0.5396 |
| Cohibin D | HMDB0035398 | Fatty alcohols | * |  | 36.2 | 0.0415 | 0.5321 |
| PC(O-16:0/18:3(9Z,12Z,15Z)) | LMGP01020042 | Glycerophosphocholines |  |  | 36.7 | 0.0262 | 0.4915 |
| 3-Oxoglutaric acid | HMDB0013701 | Short-chain keto acids and derivatives | *** |  | 38.1 | 0.0010 | 0.4298 |
| PC(14:0/18:1(9Z)) | LMGP01010492 | Glycerophosphocholines | * |  | 36.8 | 0.0138 | 0.3673 |
| Oxoglutaric acid | HMDB0000208 | Gamma-keto acids and derivatives | ** | C00026 | 39.7 | 0.0483 | 0.2632 |
| PC(16:0/O-16:0) | 76588 | Unclassified |  |  | 36.3 | 0.0008 | 0.2554 |
| PE(18:0/18:2(9Z,12Z)) | HMDB0008994 | Glycerophosphoethanolamines | * |  | 55 | 0.0426 | 0.2472 |
| PE(18:2(9Z,12Z)/18:0) | LMGP02011193 | Glycerophosphoethanolamines | * |  | 51.9 | 0.0202 | 0.2038 |
| S-Japonin | HMDB0035802 | Sesquiterpenoids | * |  | 40.7 | 0.0221 | 0.1898 |
| PC(18:0/22:4(7Z,10Z,13Z,16Z)) | LMGP01010813 | Glycerophosphocholines | * |  | 37.5 | 0.0074 | 0.1895 |
| PC(O-16:0/16:0)[U] | 40059 | Unclassified |  |  | 36.3 | 0.0000 | 0.1879 |
| S-Methyl-3-phospho-1-thio-D-glycerate | 66174 | Unclassified |  |  | 38.6 | 0.0271 | 0.1668 |
| PC(22:5(4Z,7Z,10Z,13Z,16Z)/16:1(9Z)) | HMDB0008660 | Glycerophosphocholines | ** | C00157 | 36.9 | 0.0042 | 0.1617 |
| PC(15:0/18:1(9Z)) | HMDB0007939 | Glycerophosphocholines | ** | C00157 | 36.9 | 0.0219 | 0.1432 |
| PE(18:0/20:4(5Z,8Z,11Z,14Z)) | HMDB0009003 | Glycerophosphoethanolamines | * |  | 51.3 | 0.0000 | 0.1194 |
| PC(22:0/18:4(6Z,9Z,12Z,15Z)) | HMDB0008536 | Glycerophosphocholines | ** | C00157 | 37.6 | 0.0359 | 0.1153 |
| PC(O-14:0/18:0) | LMGP01020015 | Glycerophosphocholines |  |  | 36.3 | 0.0011 | 0.1128 |
| O-methoxycatechol-O-sulphate | HMDB0060013 | Arylsulfates | * |  | 37.7 | 0.0390 | 0.0910 |
| PC(18:1(11Z)/20:4(5Z,8Z,11Z,14Z)) | HMDB0008081 | Glycerophosphocholines | * |  | 56.2 | 0.0429 | 0.0900 |
| PC(18:4(6Z,9Z,12Z,15Z)/15:0) | HMDB0008231 | Glycerophosphocholines | ** | C00157 | 36.2 | 0.0190 | 0.0792 |
| LysoPE(20:4(5Z,8Z,11Z,14Z)/0:0) | HMDB0011517 | Glycerophosphoethanolamines | * |  | 47.5 | 0.0028 | 0.0617 |
| N-(3-hydroxy-eicosanoyl)-homoserine lactone | LMFA08030008 | Fatty amides |  |  | 37.4 | 0.0248 | 0.0351 |
| 5'-Carboxy-gamma-chromanol | HMDB0012799 | 1-benzopyrans | * |  | 39 | 0.0167 | 0.0216 |
| PC(17:1(9Z)/15:0) | LMGP01011525 | Glycerophosphocholines |  |  | 53 | 0.0012 | 0.0171 |
| PC(15:0/22:6(4Z,7Z,10Z,13Z,16Z,19Z)) | HMDB0007958 | Glycerophosphocholines | ** | C00157 | 36.9 | 0.0343 | 0.0157 |
| PE(16:0/18:1(11Z)) | LMGP02010010 | Glycerophosphoethanolamines | * |  | 51.7 | 0.0003 | 0.0157 |
| Dihydroxyacetone Phosphate Acyl Ester | HMDB0011750 | Carbonyl compounds | ** | C03372 | 38.8 | 0.0264 | 0.0114 |
| PE(16:1(9Z)/22:4(7Z,10Z,13Z,16Z)) | LMGP02010538 | Glycerophosphoethanolamines | ** | C00350 | 37.2 | 0.0373 | 0.0097 |

Supplementary table 7. Differential metabolites in the FHN and sclerotic groups.

| **Metabolites** | **Compound ID** | **Sub Class** | **level** | **kegg** | **Score** | **P-value** | **FC** |
| --- | --- | --- | --- | --- | --- | --- | --- |
| Squamotacin | HMDB0030444 | Fatty alcohols | * |  | 36.8 | 0.0210 | 21.8951 |
| PG(O-16:0/20:5(5Z,8Z,11Z,14Z,17Z)) | 79885 | Glycerophosphoglycerols |  |  | 38.7 | 0.0001 | 13.1228 |
| Annomutacin | 87917 | Fatty alcohols | * |  | 36 | 0.0383 | 8.7500 |
| Muricatenol | HMDB0035900 | Fatty alcohols | * |  | 45.8 | 0.0063 | 8.4158 |
| PA(14:1(9Z)/22:2(13Z,16Z)) | 81300 | Glycerophosphates | * |  | 36.4 | 0.0458 | 7.8738 |
| Asiminenin A | HMDB0029959 | Fatty alcohols | * |  | 37.5 | 0.0140 | 6.0208 |
| TG(8:0/i-12:0/12:0) | HMDB0071313 | Triradylcglycerols | * |  | 37.5 | 0.0078 | 6.0199 |
| PC(20:1(11Z)/P-16:0) | 59770 | Glycerophosphocholines | ** | C00157 | 37.9 | 0.0417 | 2.6553 |
| PG(16:0/22:4(7Z,10Z,13Z,16Z)) | LMGP04010968 | Glycerophosphoglycerols | * |  | 38.1 | 0.0454 | 2.6505 |
| LacCer(d18:1/14:0) | 83816 | Neutral glycosphingolipids |  |  | 36.2 | 0.0059 | 2.4597 |
| PS(O-18:0/18:1(9Z)) | LMGP03020027 | Glycerophosphoserines |  |  | 36.2 | 0.0459 | 2.0999 |
| SM(d16:1/18:0) | LMSP03010042 | Phosphosphingolipids |  |  | 57.3 | 0.0335 | 2.0480 |
| Propionylcarnitine | HMDB0000824 | Fatty acid esters | *** | C03017 | 50.8 | 0.0332 | 1.9404 |
| 8,10-hexadecadiynoic acid | LMFA01030493 | Fatty Acids and Conjugates |  |  | 56.5 | 0.0363 | 1.2451 |
| Phosphohydroxypyruvic acid | HMDB0001024 | Carbonyl compounds | ** | C03232 | 38.6 | 0.0324 | 0.9455 |
| 2-(Acetamidomethylene)succinate | 63871 | Unclassified |  |  | 37.5 | 0.0089 | 0.9312 |
| Azoprocarbazine | 2106 | Unclassified |  |  | 38.7 | 0.0104 | 0.9024 |
| N-stearoyl valine | 75504 | Fatty amides |  |  | 38.8 | 0.0391 | 0.8904 |
| Flupyrsulfuron-methyl sodium | 72617 | Unclassified |  |  | 36.1 | 0.0156 | 0.8886 |
| Bismuth subsalicylate | 66756 | Unclassified |  | C07870 | 38.2 | 0.0165 | 0.8762 |
| Vinaginsenoside R1 | 90087 | Terpene glycosides | * |  | 36 | 0.0109 | 0.8255 |
| Compactin diol lactone | 69858 | Unclassified |  |  | 38 | 0.0161 | 0.8253 |
| N-(2,4-Eicosadienoyl)piperidine | HMDB0032001 | N-acylpiperidines | * |  | 36.5 | 0.0302 | 0.7244 |
| 3-Oxoglutaric acid | HMDB0013701 | Short-chain keto acids and derivatives | *** |  | 38.1 | 0.0094 | 0.6828 |
| 4-Amino-5-hydroxymethyl-2-methylpyrimidine | 3265 | Unclassified |  |  | 38.2 | 0.0341 | 0.6499 |
| Brunfelsamidine | 68140 | Unclassified |  |  | 42.8 | 0.0315 | 0.6282 |
| 3-Methylpyrrolo[1,2-a]pyrazine | HMDB0033172 | Unclassified |  |  | 43.5 | 0.0244 | 0.5037 |
| 1-(5-Hydroxy-2-pyrimidinyl)piperazine | 1453 | Unclassified |  |  | 38.3 | 0.0091 | 0.4899 |
| LysoPE(20:4(5Z,8Z,11Z,14Z)/0:0) | HMDB0011517 | Glycerophosphoethanolamines | * |  | 47.5 | 0.0043 | 0.2418 |
| PC(O-16:0/16:0)[U] | 40059 | Unclassified |  |  | 36.3 | 0.0426 | 0.1821 |
| PC(18:0/22:4(7Z,10Z,13Z,16Z)) | LMGP01010813 | Glycerophosphocholines | * |  | 37.5 | 0.0035 | 0.1369 |
